# Codon Usage Bias Analysis of Chloroplast Genomes in Six Cucurbitaceae Species

**DOI:** 10.3390/genes17091095

**Published:** 2026-09-11

**Authors:** Yongjie Xia, Zhuoran Huang

**Affiliations:** 1 College of Life Sciences, Huaibei Normal University, Huaibei 235000, China; 18805601148@163.com; 2 Anhui Province Key Laboratory of Pollutant Sensitive Materials and Environmental Remediation, Huaibei Normal University, Huaibei 235000, China

**Keywords:** codon usage bias, chloroplast genome, Cucurbitaceae, natural selection, Python

## Abstract

**Background and Objectives**: Codon usage bias (CUB), the non-uniform usage of synonymous codons, is prevalent in plant chloroplast genomes and plays important roles in gene expression and genome evolution. However, systematic comparisons of CUB patterns across genera in the Cucurbitaceae family remain limited. This study aimed to characterize CUB patterns and identify their driving forces in the chloroplast genomes of six Cucurbitaceae species. **Methods**: We analyzed the complete chloroplast genomes of six Cucurbitaceae species, watermelon (*Citrullus lanatus*), melon (*Cucumis melo*), cucumber (*Cucumis sativus*), pumpkin (*Cucurbita moschata*), wax gourd (*Benincasa hispida*), and bitter gourd (*Momordica charantia*). CUB patterns were assessed using ENC-plot, neutrality plot, and PR2-plot analyses, all implemented through a custom, reproducible Python-based workflow. **Results**: The overall codon usage bias was weak across all six species, with a clear preference for A/U-ending codons. Candidate preferred codons (hereafter referred to as optimal codons for brevity) identified independently within each species were largely shared across the six species, although species-specific codons were also detected. Combined ENC-plot, neutrality plot, and PR2-plot analyses suggest that natural selection, rather than mutation pressure, is the dominant force shaping CUB in these genomes, although these approaches provide indirect evidence. **Conclusions**: These findings provide a reference for codon optimization of exogenous genes in chloroplast genetic engineering of Cucurbitaceae crops. The Python-based workflow developed in this study also offers a transparent, reproducible alternative to conventional CUB analysis approaches.

## 1. Introduction

The degeneracy of the genetic code, 18 amino acids encoded by 2 to 6 synonymous codons, makes the non-random usage of synonymous codons a significant phenomenon in genome evolution. This codon usage bias (CUB) has been widely documented in bacteria, plants, and animals, and is shaped by the combined effects of mutation pressure, natural selection, and genetic drift [1].

CUB is shaped by multiple factors, among which natural selection and mutation pressure are considered the two most prominent forces. Extensive studies have shown that angiosperm chloroplast genomes generally exhibit A/T base enrichment and a preference for A/U-ending codons. To investigate the dominant factors of CUB, researchers typically employ three analytical approaches, namely the effective number of codons (ENC) plot, the neutrality plot, and the parity rule 2 (PR2) plot, which assess codon preference intensity, GC content correlation, and base usage parity, respectively. Based on these methods, natural selection has been identified as the primary factor shaping codon usage patterns in chloroplast genomes across multiple plant lineages, including *Chloranthus* [2], Ampelopsideae [3], Gnetales [4], and *Coffea* [5]. Natural selection shapes codon bias by favouring codons that pair with the most abundant tRNAs, thereby enhancing translational efficiency and accuracy [6,7]. In the literature, this force is recognized by conserved optimal codons across related taxa, a decoupling of GC12 from GC3, and genes falling below the expected ENC curve [2,3,4,5,8]. Mutation pressure, in contrast, acts genome-wide and uniformly across all codon positions through AT- or GC-biased mutation [9,10]; it is reported to produce strong positive GC12–GC3 correlations, with points lying on the expected ENC curve [9]. At the whole-plant level, this distinction is illustrated by highly expressed photosynthetic genes (e.g., psbA), which often show stronger codon adaptation than low-expression genes (selection) [7], whereas genome-wide GC shifts reflect mutation pressure. In this study, the two forces are parsed as follows: genes below the expected ENC curve and non-significant GC12–GC3 regression point to natural selection, whereas points on the expected curve and significant GC12–GC3 regression point to mutation pressure [10,11]; the PR2-plot further tests whether third-position base composition deviates from parity (selection) or conforms to it (mutation) [12,13]. Disentangling these two forces matters because it informs our understanding of chloroplast genome evolution and has implications for codon-optimization strategies in chloroplast transgenes [14].

Substantial evidence indicates that angiosperm chloroplasts generally prefer A/U-ending codons [8]. Still, interpretations of the underlying driving forces remain divided: one school argues that natural selection is the dominant factor [6,7,15], while another contends that context-dependent mutation dynamics are sufficient to explain most of the observed patterns [9]. This debate underscores the need for accumulating more comparative data across diverse lineages.

Cucurbitaceae includes important horticultural crops such as watermelon, melon, cucumber, and pumpkin. Chloroplast genomes of multiple species in this family have been sequenced, and codon usage studies have been conducted on *Gynostemma*, *Actinostemma tenerum*, *B. hispida*, and *Cucurbita* [16].

Substantial chloroplast codon usage studies have been conducted in Cucurbitaceae genera including *Gynostemma* [17,18], *Actinostemma* [19], *Benincasa* [20], and *Cucurbita* [21], all of which reported weak overall bias, a preference for A/U-ending codons, and natural selection as the dominant force. A broad comparison across 10 representative species also revealed highly conserved chloroplast genome structures and relatively consistent codon usage at the family level [16]. However, most existing studies have focused on single genera or single species, and a systematic, methodologically consistent cross-generic comparison integrating ENC-plot, neutrality plot, PR2-plot, correlation, and optimal-codon analyses under an identical pipeline remains limited.

The Cucurbitaceae family comprises some of the most economically important horticultural crops worldwide, including watermelon (*C. lanatus*), melon (*C. melo*), cucumber (*C. sativus*), pumpkin (*C. moschata*), wax gourd (*B. hispida*), and bitter gourd (*M. charantia*) [22]. These six species are cultivated across tropical, subtropical, and temperate regions and are valued not only as staple vegetables and fruits but also for their nutritional composition, minerals, vitamins, dietary fiber, and a range of bioactive compounds with health-promoting properties (e.g., antioxidant, antidiabetic, and hypolipidemic activities) [23,24]. Their rapid vegetative growth and fruit development are largely supported by photosynthesis in source leaves and by the transport and allocation of assimilates [25].

The chloroplast genome is directly involved in these processes. It encodes components of the photosynthetic electron-transport chain (photosystems I and II, the cytochrome b6/f complex), the ATP synthase, the NADH dehydrogenase complex, the large subunit of ribulose-1,5-bisphosphate carboxylase/oxygenase (Rubisco), and the genetic-system components required for their expression [14]. These gene products couple light-driven energy conversion (ATP and NADPH production) with CO2 fixation and ultimately with biomass accumulation. Genome-wide forces that shape chloroplast gene sequence and expression, including synonymous codon usage, can therefore, in principle, modulate photosynthetic efficiency and biomass production, which highlights the agronomic relevance of codon usage studies in these species [16]. Recent advances in chloroplast genome editing tools have expanded the potential for engineering photosynthetic efficiency and agronomic traits in crops [26]. For example, CRISPR/Cas9-mediated truncation of light-harvesting antenna genes in rice increased photosynthetic efficiency and biomass productivity under dense canopy conditions [27], demonstrating that modifications of chloroplast-targeted genes can directly translate into improved photosynthetic performance. As efficient transgene expression in chloroplasts depends on codon compatibility with the host translational machinery, a thorough understanding of endogenous codon usage patterns is a prerequisite for rational codon optimization in chloroplast engineering. The codon usage data generated in this study may therefore serve as a useful reference for future efforts in this direction.

Species-level codon usage information within Cucurbitaceae remains uneven. In addition to the plastome-level studies of wax gourd and *Cucurbita* summarized above, dedicated chloroplast codon usage analyses have been reported for melon, where Hu et al. [28] showed that all codons ending in A or U in nine melon chloroplast genomes exhibited RSCU values > 1, revealing a conserved A/U-ending preference. By contrast, for watermelon, cucumber, and bitter gourd, only reference or assembled chloroplast genomes are available [29,30,31], and no dedicated chloroplast codon usage bias analysis has been reported to our knowledge. At the nuclear-genome level, Niu et al. [32] comprehensively compared the codon usage patterns of pumpkin with nine other Cucurbitaceae species and identified 30 optimal codons in cucumber, most ending with U or A, under a weak overall bias. Nevertheless, a systematic and methodologically consistent comparison of chloroplast codon usage bias across representative tribes of Cucurbitaceae, integrating ENC-plot, neutrality plot, PR2-plot, correlation, and optimal-codon analyses under an identical pipeline, remains limited.

**Figure 1:**
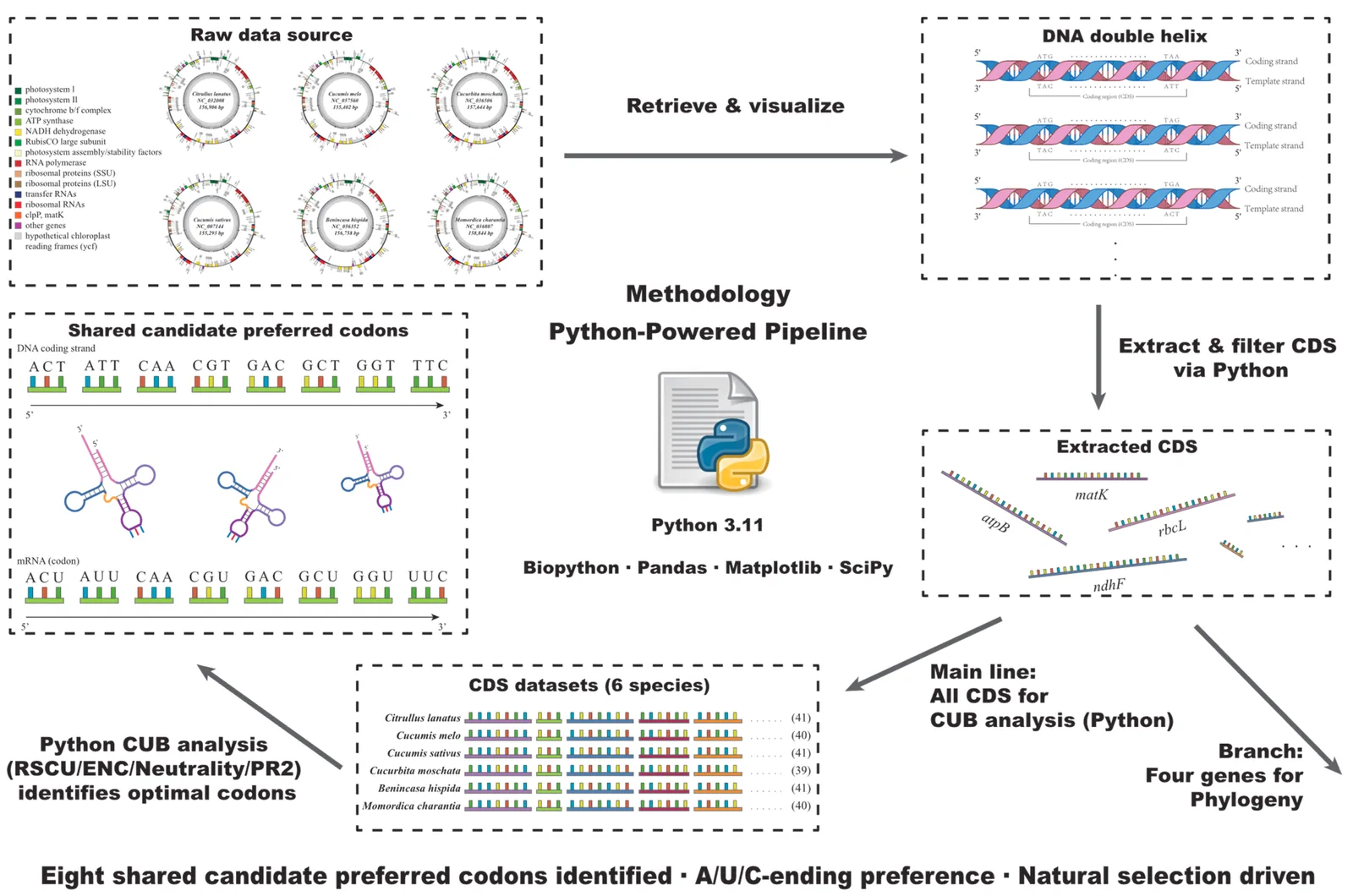
Overall workflow of this study. Note: From left to right, the five panels illustrate the main analytical pipeline: the chloroplast genome circle maps (drawn by OGDRAW) indicate the data source; the chloroplast genome sequences were annotated using the GeSeq online platform to generate GenBank (.gb) files, from which CDS sequences were extracted and filtered via custom Python scripts, yielding CDS datasets of six species (numbers in parentheses indicate the number of filtered CDSs retained for each species after applying the criteria of ATG initiation and ≥300 bp); these datasets were then subjected to Python-based codon usage bias analysis (RSCU, ENC-plot, neutrality plot, PR2-plot) and candidate preferred codon identification. Among these, four representative genes (*atpB*, *matK*, *ndhF*, *rbcL*) were selected for phylogenetic reconstruction. The Python icon denotes Python-based implementation throughout the workflow.

In this study, we selected six species, namely watermelon, melon, cucumber, pumpkin, wax gourd, and bitter gourd, belonging to the tribes Benincaseae, Cucurbiteae, and Momordiceae [33], to systematically compare their chloroplast genome codon usage patterns and quantify the relative contributions of natural selection and mutation pressure. This work may serve as a reference for the optimization of exogenous genes in chloroplast genetic engineering of Cucurbitaceae species. All analyses were primarily performed using Python 3.11.9. The research workflow is illustrated in Figure 1.

## 2. Materials and Methods

### 2.1. Chloroplast Genome Sequence Retrieval

The complete chloroplast genome sequences of six Cucurbitaceae species were downloaded from the NCBI RefSeq database (https://www.ncbi.nlm.nih.gov/refseq/, accessed on 7 July 2026) as fully assembled [34], namely *C. lanatus* (NC_032008), *C. melo* (NC_057560), *C. sativus* (NC_007144), *C. moschata* (NC_036506), *B. hispida* (NC_056352), and *M. charantia* (NC_036807). All sequences were obtained from public databases to ensure the reproducibility of the analysis. The assembled chloroplast genome sequences were then submitted to the GeSeq online platform (https://chlorobox.mpimp-golm.mpg.de, accessed on 7 July 2026) [35,36] for annotation, and the resulting GenBank (.gb) annotation files were downloaded for the subsequent extraction of protein-coding sequences. The OGDRAW module of the same platform was used to visualize each chloroplast genome and generate circular genome structure maps (Figure S1).

### 2.2. CDS Extraction and Filtering

Protein-coding sequences (CDSs) were extracted from the GenBank (.gb) annotation files obtained in Section 2.1 using custom Python scripts based on the Biopython library [37]. For each CDS feature, the coding sequence was extracted using Biopython’s location-aware extraction (‘feature.location.extract’), which automatically concatenates exons (excluding introns) and reverse-complements reverse-strand genes according to the annotated coordinates. Genes annotated as pseudogenes are not represented as CDS features and were therefore not extracted. To ensure the reliability of downstream analyses [19,38], CDSs were filtered according to the following criteria:

1. Sequences must start with the start codon ATG;
2. Sequences must be longer than 300 bp;
3. Redundant gene copies were removed, retaining only one copy per gene per species.

Because some plastid genes are initiated with non-ATG start codons (e.g., GTG) [39,40], and because the >300-bp criterion excludes short CDSs, the sensitivity of the results to both filters was examined explicitly by re-running the entire analysis under progressively relaxed criteria: (i) the manuscript filters (ATG initiation, ≥300 bp); (ii) relaxed initiation (ATG, GTG or TTG, ≥300 bp), which retained one to two additional genes per species; and (iii) all annotated CDSs, with no initiation or length filter (80 genes per species), under which the shared set was reduced to four codons. Relaxing the start-codon criterion left the optimal codons identified in every species, and the eight codons shared by all six species, completely unchanged. Varying the length threshold between 200 and 300 bp retained all eight shared codons (a ninth codon was additionally recovered at 200–250 bp); at 150 bp, the relaxed start-codon rule retained seven of the eight, whereas a 400-bp threshold reduced the shared set to seven. Removing the length filter altogether admitted very short CDSs (*psb30*, *psbT* and *rpl36*; 75–114 bp, i.e., 25–38 codons), for which per-gene RSCU and ENC cannot be estimated with meaningful precision, and the shared set collapsed to two codons. The 300-bp criterion was therefore retained because it lies within a broad range (200–300 bp) over which the results are stable, and because it excludes genes too short for reliable per-gene codon-usage estimation. We note, however, that the excluded CDSs are not a random sample of the annotation: they are markedly shorter (median 201 bp vs. 711 bp for the retained CDSs) and GC3-richer (mean GC3 0.340 vs. 0.281), so the filtering criteria do shape the composition of the gene sample, and the reported parameters should be interpreted as describing the retained set of protein-coding genes of substantial length rather than the complete plastid CDS repertoire (detailed results provided in the Supplementary Code).

### 2.3. Codon Usage Analysis

All subsequent codon usage analyses were performed using Python 3.11.9 with the Biopython (v1.87), pandas (v2.3.3), NumPy (v2.3.5), Matplotlib (v3.11.0), SciPy (v1.17.1), and Seaborn (v0.13.2) packages. The following parameters were calculated for each gene and each species: GC content at the first, second, and third codon positions (GC1, GC2, GC3), overall GC content (GCall), and the average GC content of the first two positions (GC12). Throughout the manuscript, “A/U-ending” is used to describe codon preferences at the translational level, corresponding to A/T at the genomic DNA sequence level; “A/T” is retained when describing genome-wide base composition. The relative synonymous codon usage (RSCU) [41] values were calculated for each codon according to the bacterial, archaeal, and plant plastid code (NCBI translation table 11), which is the translation table used in chloroplast genomes [42,43], and the results were exported as tabular files. RSCU data were then organized and formatted using Microsoft Excel for Microsoft 365 (Microsoft Corp., Redmond, WA, USA), and RSCU bar plots were generated using the online visualization platform BioDataTools (https://www.biodatatools.com, accessed on 8 July 2026) to illustrate the relative usage frequency of each codon, while RSCU heatmaps were generated using Python with the seaborn library to visualize the similarity of codon usage patterns across species. The effective number of codons (ENC) was calculated using CodonW (version 1.4.2) to evaluate the extent of codon usage bias for each gene.

### 2.4. Neutrality Plot, ENC-Plot, and PR2-Plot Analysis

Neutrality plot analysis was performed by plotting GC12 against GC3 for each gene to evaluate the relative contributions of mutation pressure and natural selection to codon usage bias [10]. The neutrality plot was generated using Microsoft Excel based on the calculated GC12 and GC3 values. Regression significance was evaluated using a two-tailed *t*-test (*df* = *n* − 2).

ENC-plot analysis was conducted by plotting ENC values against GC3 content, with the expected ENC curve calculated according to the following formula. ENC value lower than 35 is considered to indicate strong codon usage bias, whereas higher values suggest weak bias [11]:

(1) $$ENC=2+GC3+\frac{29}{\left[GC3^{2}+{\left(1-GC3\right)}^{2}\right]}$$

PR2-plot (parity rule 2 plot) analysis was carried out by plotting A3/(A3+T3) against G3/(G3+C3) for each gene to examine the parity of nucleotide usage at the third codon position [12,13]. Both the ENC-plot and PR2-plot were generated using the Matplotlib package (v3.11.0) in Python.

### 2.5. Identification of Optimal Codons

Optimal codons were identified by comparing high-codon-bias and low-codon-bias gene sets. For each species, all genes were ranked by their ENC values, and the top 10% (low ENC, strongest bias) and bottom 10% (high ENC, weakest bias) were selected to construct the high-codon-bias and low-codon-bias gene sets, respectively. Because ENC measures the strength of codon usage bias rather than transcript abundance, these groups are referred to as high- and low-codon-bias sets, and no inference of expression level is involved. The ΔRSCU value for each codon within each species was calculated as the difference in RSCU between the high-codon-bias and low-codon-bias gene sets. Optimal codons were identified independently within each species using two criteria: (i) the RSCU averaged across the genes of the high-codon-bias set exceeds 1.00, i.e., the codon is preferentially used among strongly biased genes; and (ii) the difference in RSCU between the high- and low-codon-bias sets (ΔRSCU) is no less than 0.08 [4,5,7].

### 2.6. Phylogenetic Analysis

Phylogenetic analyses were conducted using two distinct datasets. First, the complete chloroplast genome sequences of the six species were merged and aligned using the MAFFT online server (https://mafft.cbrc.jp/alignment/server/, accessed on 13 August 2026). Second, four representative chloroplast genes (*atpB*, *matK*, *ndhF*, and *rbcL*) were extracted from the filtered CDS of each species, concatenated in the order *atpB-matK-ndhF-rbcL*, and aligned using MEGA11 (v11.0) [44]. For both datasets, maximum likelihood (ML) [45] phylogenetic trees were reconstructed with 1000 bootstrap replicates, using the Tamura-Nei (TN93) nucleotide substitution model in MEGA11 (v11.0). Gaps and missing data were treated by complete deletion. The four concatenated genes were analyzed as a single partition without gene-wise partitioning.

To further verify the stability of the resulting topology, *Punica granatum* was introduced as an outgroup. The corresponding four genes were extracted and concatenated from the *P. granatum* chloroplast genome following the same procedure. The concatenated sequences of all seven species (six Cucurbitaceae species plus the outgroup) were then aligned and used to reconstruct an ML tree using the same methods and parameters, with *P. granatum* serving as the outgroup to confirm the tree’s root position.

### 2.7. Statistical Analysis

Pearson correlation coefficients and corresponding *p*-values among codon usage parameters (ENC, GC1, GC2, and GC3) were calculated using the scipy.stats module in (SciPy v1.17.1) Python 3.11.9 [1,46,47]. The results were exported as tabular files and organized using Microsoft Excel. A correlation heatmap was then generated using the seaborn library in Python to visualize the degree of correlation among the parameters. Significance levels were annotated with asterisks (* *p* < 0.05, ** *p* < 0.01, *** *p* < 0.001).

## 3. Results

### 3.1. Codon Usage Bias Analysis

Table 1 summarizes the codon usage parameters of the six species. The mean ENC values ranged from 47.0 to 47.6, well above the threshold of 35 that is commonly used to indicate a strong codon usage bias, suggesting an overall weak bias in these chloroplast genomes. The GC content differed markedly among codon positions: mean GC1 (46.7–46.9%) and GC2 (39.4–39.6%) were substantially higher than mean GC3 (27.8–28.4%), and the overall GC content ranged from 38.0% to 38.2%, consistent with the A/T-rich composition typical of chloroplast genomes. These observations set the basis for the analyses below. We first examined the relative synonymous codon usage (RSCU) to determine how the bias is distributed among synonymous codons. The ENC-plot was then used to evaluate the strength of the bias and its relationship with GC3, followed by correlation analysis of the codon usage parameters. Finally, the PR2-plot was used to test whether base composition at the third codon position deviates from parity.

#### 3.1.1. RSCU Analysis

**Figure 2:**
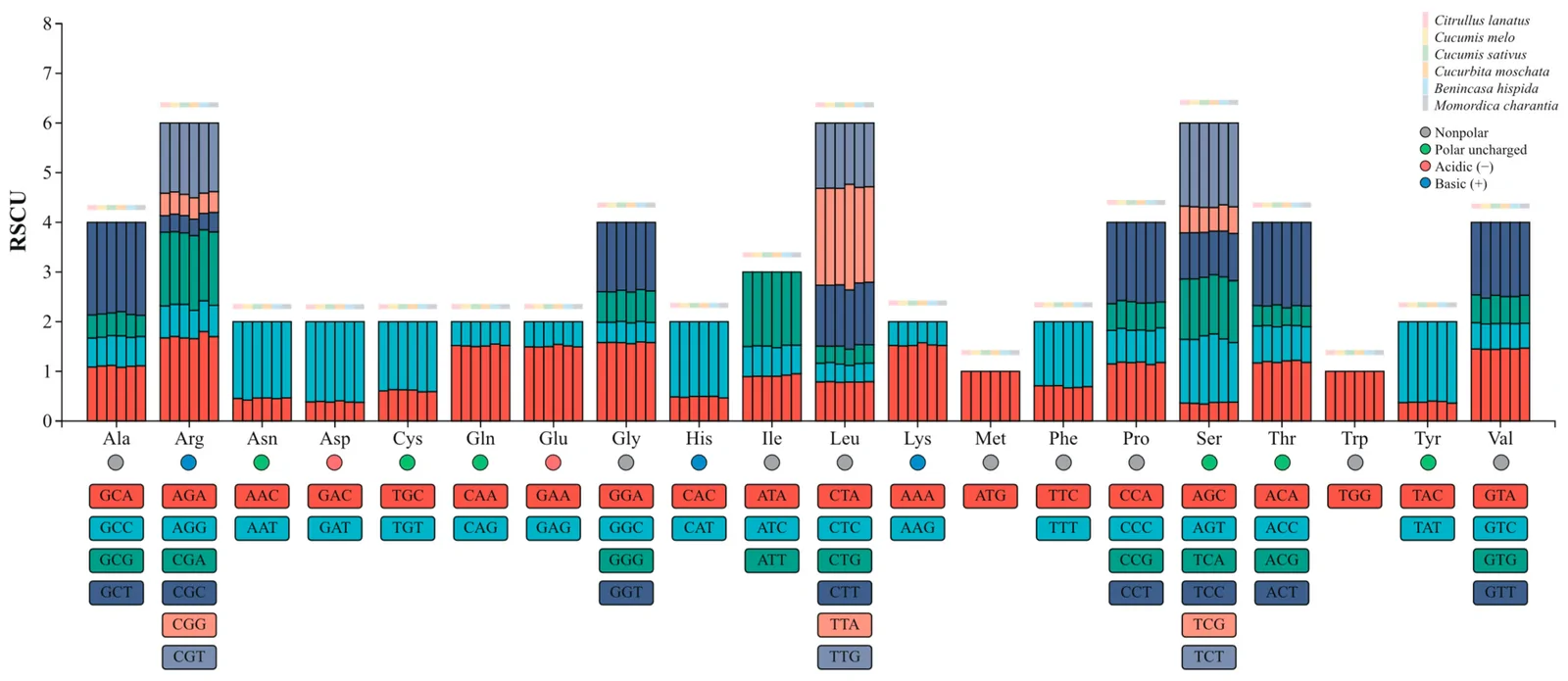
RSCU bar plot. Amino acids are grouped and color-coded by physicochemical property (nonpolar hydrophobic, polar uncharged, basic, acidic); within each amino acid, bars for the six species are shown side by side in six distinct colors.

The RSCU heatmap (Figure S2) and bar plot (Figure 2) together illustrate the codon usage patterns across the six species. In the heatmap, codon preferences varied along a continuous gradient, with GCT (Ala) and TTA (Leu) showing the most intense red coloration across all species, indicating the strongest usage bias. A clear distinction was observed between codons with higher RSCU values (RSCU > 1), most of which ended with A or U, and those with lower values (RSCU < 1), which predominantly ended with G or C. The bar plot (Figure 2) further confirmed that the RSCU rankings within each amino acid were largely consistent across the six species, and the preference for A/U-ending codons remained evident across all amino acid types. Leucine, arginine, and serine are each encoded by six synonymous codons, and the top-ranked codons by RSCU within each amino acid all ended with A or U (Table S1).

#### 3.1.2. ENC-Plot Analysis

**Figure 3:**
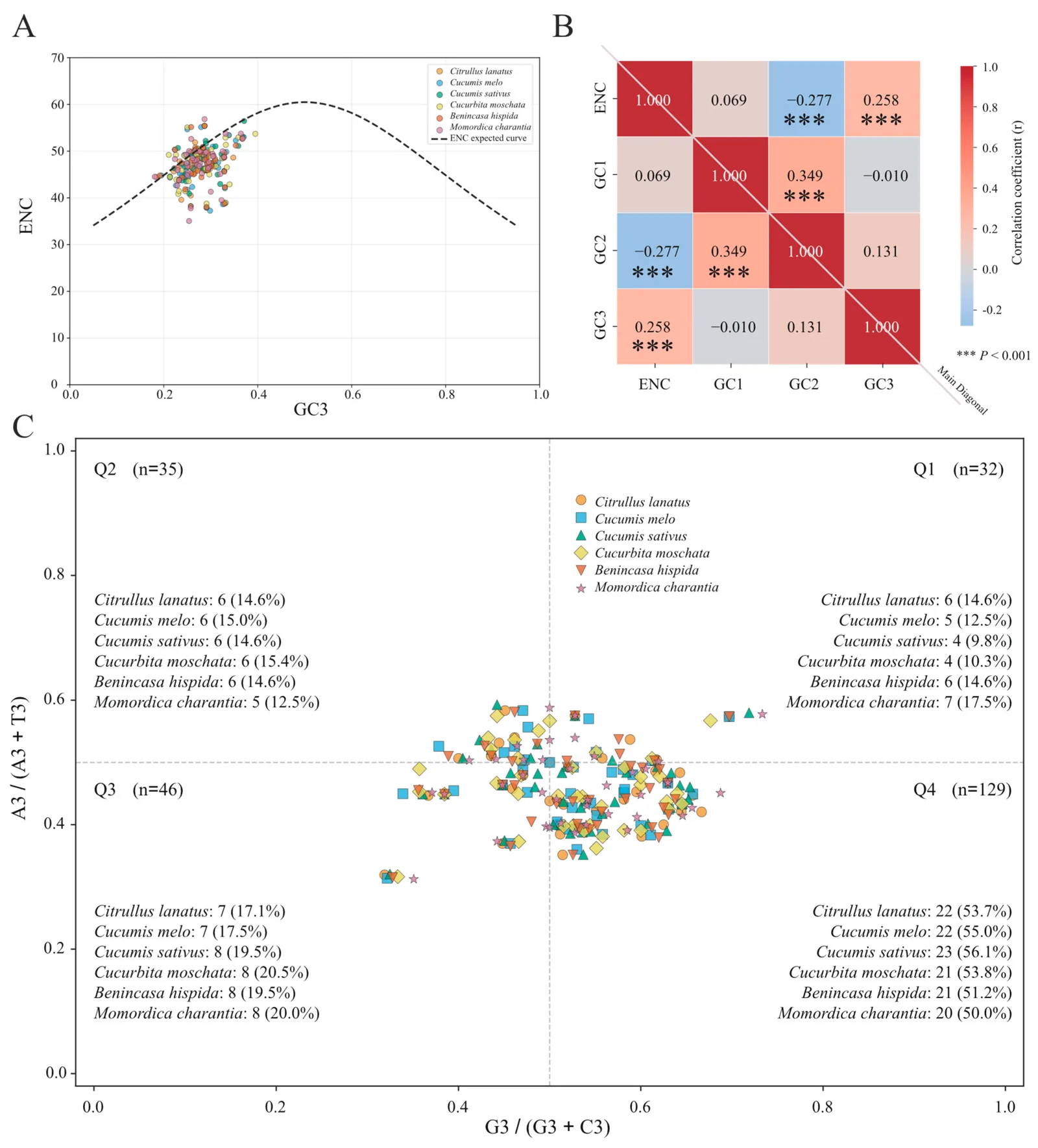
Codon usage bias analysis of chloroplast genomes from six Cucurbitaceae species. (**A**) ENC-plot with GC3 on the *x*-axis and ENC on the *y*-axis. The black dashed curve represents the expected ENC values under neutral expectation. (**B**) Correlation heatmap showing Pearson correlation coefficients and significance levels among ENC, GC1, GC2, and GC3 (***, *p* < 0.001). (**C**) PR2-plot with G3/(G3+C3) on the *x*-axis and A3/(A3+T3) on the *y*-axis. The center point (0.5, 0.5) indicates parity. Q1 (A3 > T3, G3 > C3), Q2 (A3 > T3, G3 < C3), Q3 (A3 < T3, G3 < C3), and Q4 (A3 < T3, G3 > C3) indicate the quadrant-specific base-composition biases; the number and proportion of genes in each quadrant are labeled within the plot.

The ENC-plot distribution patterns of the six species were generally consistent (Figure 3A). The expected ENC curve increased progressively within the GC3 range of 0.2 to 0.4 and reached its theoretical maximum of approximately 60 at GC3 ≈ 0.5. The actual ENC values of the six species ranged from 35 to 60, with most genes falling within the 40–50 interval. Within the GC3 range of 0.2 to 0.4, most genes were distributed below the expected curve, while a minority were observed above it; nevertheless, the overall trend remained predominantly below the expected values. The GC3 content was concentrated in the range of 0.2 to 0.4 (Table S2), showing a linear distribution trend.

#### 3.1.3. Correlation Analysis

Correlation analysis revealed that ENC was significantly negatively correlated with GC2 (*r* = −0.2773, ***, *p* < 0.001) and significantly positively correlated with GC3 (*r* = 0.2584, ***, *p* < 0.001), but showed no significant correlation with GC1 (*r* = 0.0692, *p* = 0.284). Among GC content parameters, GC1 was significantly positively correlated with GC2 (*r* = 0.3494, ***, *p* < 0.001). GC3 exhibited no significant correlation with GC1 and GC2 (Figure 3B). Because homologous genes among the six chloroplast genomes are not fully independent observations, the correlation analyses were repeated separately within each species (*n* = 39–41 genes per species) and at the gene level (*n* = 41, each gene represented by the mean of each parameter across the six species), with Benjamini–Hochberg FDR correction applied within each analysis level. Effect sizes were consistent across the pooled, per-species, and gene-level analyses (e.g., ENC-GC2: pooled *r* = −0.277; per-species *r* = −0.359 to −0.244, mean −0.279; gene-level *r* = −0.305; GC1-GC2: pooled *r* = +0.349; per-species mean +0.349; gene-level +0.355), indicating that the pooled pattern reflects associations present within species rather than an artifact of pooling. Per-species coefficients and FDR-corrected *p* values are provided in the Supplementary Materials.

#### 3.1.4. PR2-Plot Analysis

PR2-plot analysis showed that the third codon position usage of all six species was biased toward the lower region, predominantly falling into the Q4 quadrant, indicating T3 > A3 and G3 > C3 (Figure 3C; Table S3). The distribution patterns were generally consistent across the six species.

### 3.2. Neutrality Plot Analysis

**Figure 4:**
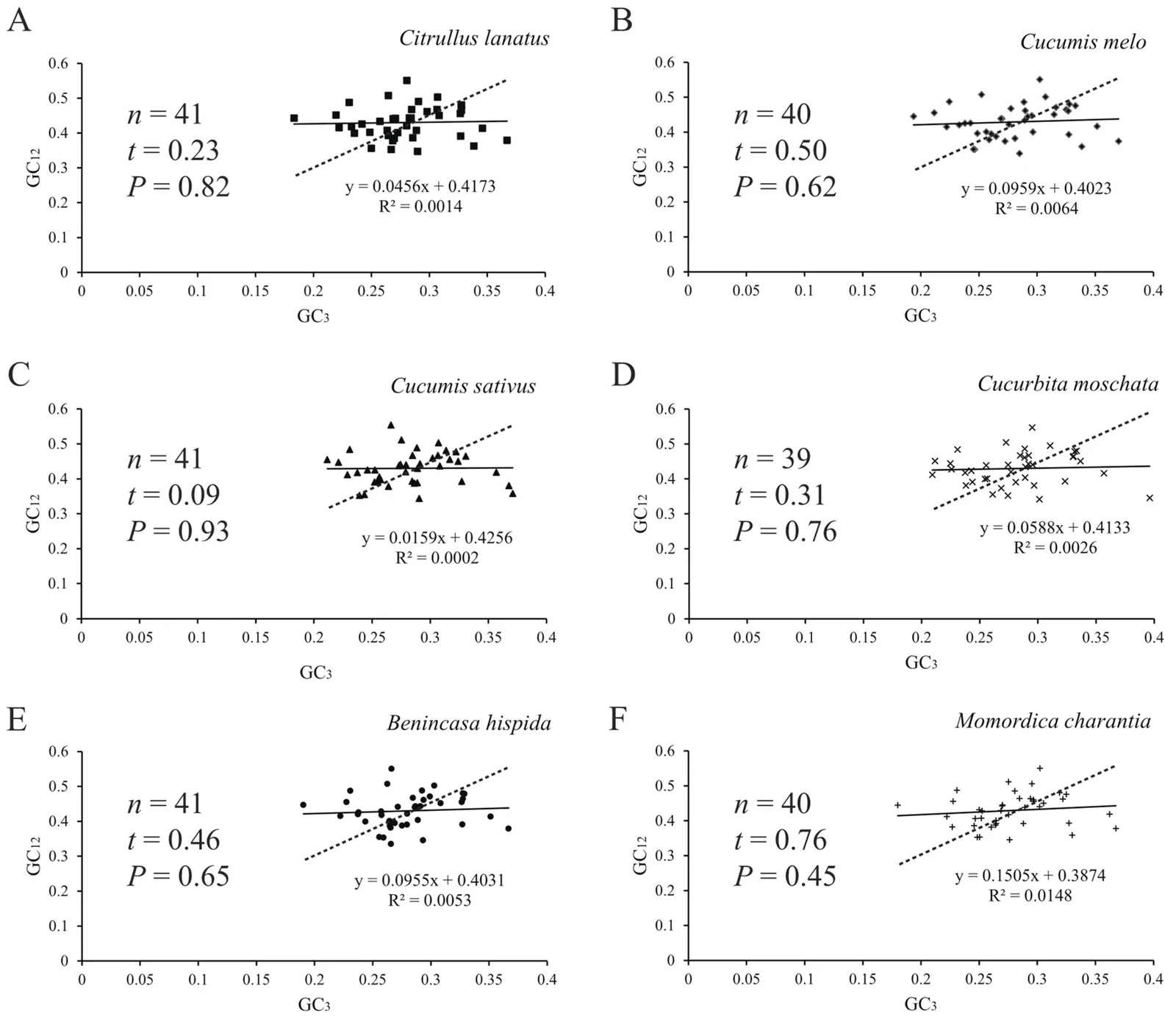
Neutrality plot analysis of chloroplast genomes from six Cucurbitaceae species. (**A**) *C. lanatus*; (**B**) *C. meloo*; (**C**) *C. sativus*; (**D**) *C. moschata*; (**E**) *B. hispida*; (**F**) *M. charantia*. The *x*-axis represents GC3, and the *y*-axis represents GC12. Solid lines indicate regression lines; dashed lines indicate the diagonal (*y* = *x*). Regression equations and R^2^ values are labeled below the points in each subpanel. Regression significance was assessed by *t*-test (two-tailed); none of the regressions were statistically significant (all *p* > 0.05).

Neutrality plot analysis was performed to evaluate the relative contributions of mutation pressure and natural selection to codon usage bias. The regression slopes of GC12 against GC3 across the six species were consistently low, ranging from 0.016 to 0.151, with R^2^ values of 0.0002–0.0148 (Figure 4; Table S4). The GC3 values of the genes were predominantly distributed between 0.2 and 0.35, while GC12 values ranged from 0.3 to 0.55, with the points being relatively scattered and showing no clear linear clustering. The distribution patterns were generally consistent across the six species, with only a few outlier genes extending beyond the aforementioned ranges. None of the six regressions was statistically significant (*p* = 0.455–0.930; Table S5), indicating that GC12 was not significantly correlated with GC3 in any of the six species. Given the very low explanatory power of these regressions, we refrain from a quantitative partition of mutation pressure and natural selection.

### 3.3. Phylogenetic Tree Construction and Topology Analysis

#### 3.3.1. Selection of Four Representative Genes

Four representative chloroplast genes (*atpB*, *matK*, *ndhF*, and *rbcL*) were selected for multi-gene concatenation analysis (Table 2). These four genes are present in all six species and have moderate sequence lengths. They are widely used molecular markers in chloroplast phylogenetic studies [47,48].

#### 3.3.2. Phylogenetic Tree Construction

**Figure 5:**
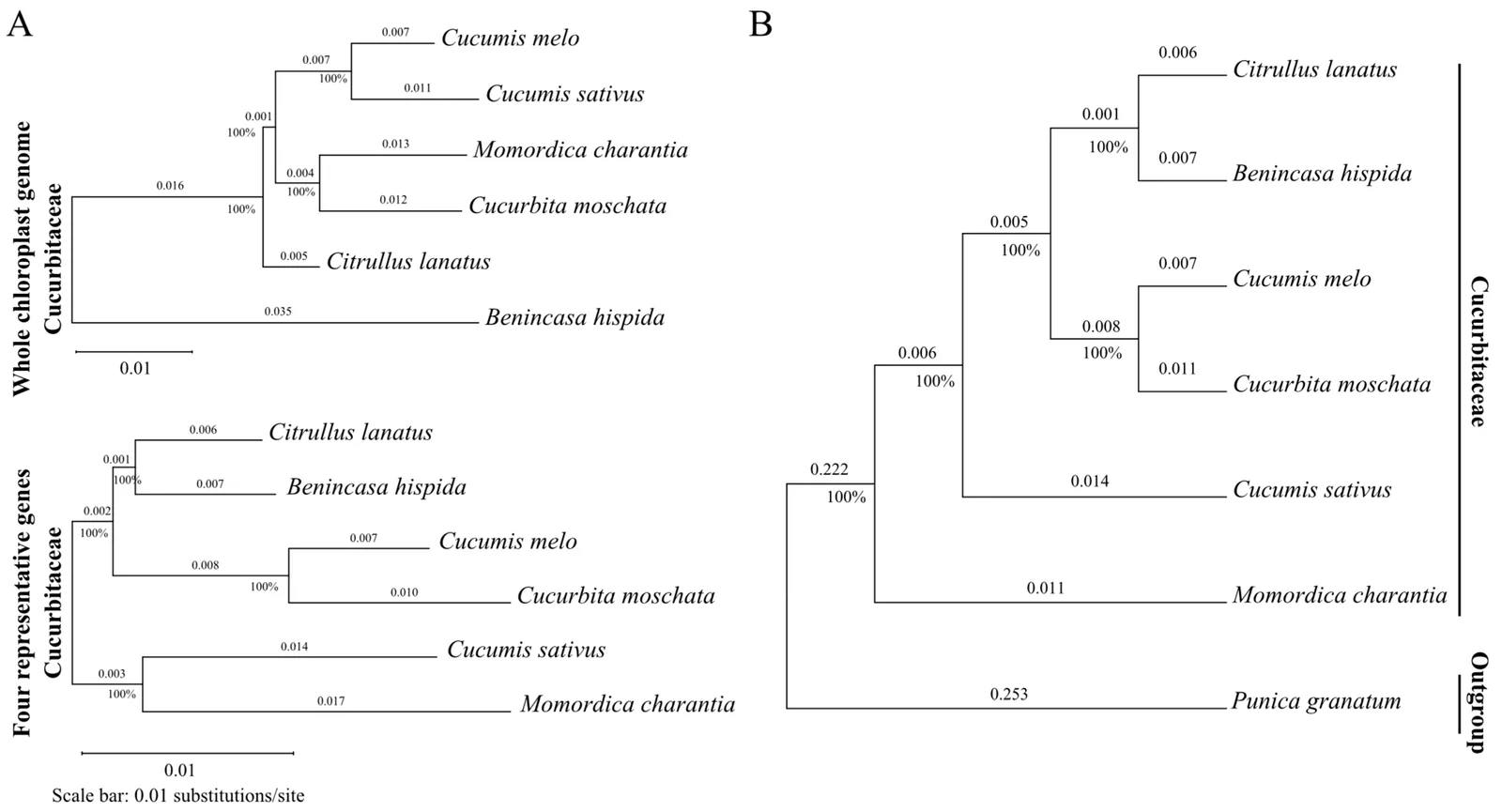
Maximum likelihood phylogenetic trees of six Cucurbitaceae species. (**A**) Upper panel: tree inferred from complete chloroplast genome sequences. Lower panel: tree inferred from concatenated sequences of four chloroplast genes (*atpB*, *matK*, *ndhF*, and *rbcL*). (**B**) Tree based on the four concatenated genes, rooted with *P. granatum* as the outgroup. Scale bar (0.01) represents nucleotide substitutions per site. Bootstrap support values (1000 replicates) are shown at nodes.

The maximum likelihood (ML) phylogenetic tree based on the concatenated sequences of the four genes is shown in Figure 5A. The six species formed two major clades: *M. charantia* and *C. sativus* clustered together as one clade, while the other clade contained *C. lanatus*, *B. hispida*, *C. melo*, and *C. moschata*, with *C. lanatus* and *B. hispida* grouped as one subclade, and *C. melo* and *C. moschata* as the other subclade.

The tree inferred from complete chloroplast genome sequences (Figure 5A, upper panel) showed a different topology: *B. hispida* diverged first from the remaining five species, followed by *C. lanatus*. The remaining four species further split into two pairs: *C. melo* with *C. sativus*, and *C. moschata* with *M. charantia*.

To further determine the root position of the tree and verify the stability of the topology, *P. granatum* was introduced as an outgroup, and the phylogenetic tree was reconstructed using the same methods (Figure 5B). With the introduction of the outgroup, *P. granatum* was positioned at the base of the tree. Among the six target species, *M. charantia* was placed at the basal-most position of the ingroup, followed by *C. sativus*, while the remaining four species formed a clade in which *C. lanatus* and *B. hispida* grouped, and *C. melo* and *C. moschata* grouped. The ingroup topology remained generally consistent with that of the four-gene tree (Figure 5A, lower panel), and all branches received 100% bootstrap support (Figure 5B).

Branch length analysis revealed that *M. charantia*, *C. sativus*, *C. melo*, and *C. moschata* had relatively longer total branch lengths, while *C. lanatus* and *B. hispida* had shorter ones, indicating differences in the degree of genetic differentiation among the chloroplast genome sequences of these species.

### 3.4. Identification and Comparison of Optimal Codons

To identify the specific codons preferentially used in Cucurbitaceae chloroplast genomes under natural selection, candidate preferred codons (hereafter referred to as optimal codons for brevity) were screened independently within each species, requiring RSCU > 1 and ΔRSCU ≥ 0.08 within that species, following the RSCU distribution patterns and the selective pressure signals observed in the ENC-plot analysis. The number of optimal codons ranged from 10 (*M. charantia*) to 17 (*C. melo*) among the six species. Eight codons, ACT, ATT, CAA, CGT, GAC, GCT, GGT, and TTC, were shared by all six species, whereas the remaining optimal codons were unique to a single species or shared by only a subset of species (Table 3 and Table S5).

Because each species retained only 39–41 genes, the high- and low-codon-bias groups used to compute ΔRSCU contained three to four genes; the sensitivity of the identification was therefore examined explicitly. The shared set was unchanged (eight codons) for ΔRSCU thresholds of 0.04–0.12, whereas varying the group size (5–25% of genes) changed the number of shared codons (5, 8, 4, 3, and 3, respectively), indicating that the exact composition of the set is sensitive to this choice. Bootstrap resampling of genes (1000 replicates) showed that four of the eight shared codons (ACT, ATT, CAA, and GAC) were recovered in at least 62% of replicates in every species, whereas the remaining four (CGT, GCT, GGT, and TTC) were recovered in 39–76% of replicates depending on the species; leave-one-out removal of individual group members frequently exchanged marginal members but rarely removed the four core codons (6 of 46 trials). The group composition was biologically consistent across species: psbA and rps8 belonged to the high-codon-bias group in all six genomes, and rpl2 to the low-codon-bias group in all six. The eight codons are therefore presented as candidate preferred codons identified under the pre-specified criterion, of which ACT, ATT, CAA, and GAC form the most robust core (detailed results provided in the Supplementary Materials).

## 4. Discussion

### 4.1. High Conservation of Codon Usage Preferences

Analysis of codon usage bias in chloroplast genomes of the six Cucurbitaceae species showed that the six species share eight optimal codons (ACT, ATT, CAA, CGT, GAC, GCT, GGT, and TTC), five ending with T (U), one with A, and two with C. The shared set indicates that codon usage is largely conserved among the six cultivated Cucurbitaceae species examined here, spanning five genera, although each species also carries species-specific optimal codons (Table 3).

### 4.2. Natural Selection as the Dominant Force

Several lines of evidence are consistent with natural selection playing a dominant role, although none of these analyses constitutes a direct test of selection. The non-significant GC12–GC3 correlation in the neutrality plot provides the primary line of evidence against a dominant role for mutation pressure, while the ENC-plot and PR2-plot offer complementary descriptive information. First, the effective number of codons (ENC) is widely used to measure the extent of codon usage bias, with lower values indicating stronger bias [11]. In the ENC-plot, the actual ENC values of genes in the six Cucurbitaceae species ranged from 35 to 60, with most genes falling within the 40 to 50 interval, while the expected curve peaked at approximately 60. Across all six species, the majority of genes (83.9%, 203 out of 242) were distributed below the expected ENC curve, while 16.1% (39 genes) were observed above it; nevertheless, the overall trend remained predominantly below the expected values. Similar patterns have been observed in the chloroplast genomes of other plant lineages, including *Chloranthus* [2], *Styphnolobium* [55], Annonaceae [56], *Miscanthus* [57], and *Manglietia* [58], where ENC values also fell below the expected curve; however, this pattern is not specific to selection and may also reflect mutational context, nucleotide composition, or gene-specific constraints. Second, neutrality plot analysis is commonly used to assess the relative contributions of mutation pressure and natural selection to codon usage bias [10]. In the present study, the regression slopes of GC12 against GC3 were low (0.016–0.151) and statistically non-significant (all *p* > 0.05), indicating that GC12 was not significantly correlated with GC3. Consistent with this, comparable low slopes have been reported in *Malus* chloroplast genomes (slope = 0.095), which likewise suggested a weak role of mutation pressure [59]. If mutation pressure were dominant, GC12 and GC3 would be expected to co-vary under the same genome-wide mutational bias; the observed lack of correlation therefore argues against a dominant mutational explanation. Third, PR2-plot analysis examines whether the four nucleotides are used evenly at the third codon position, with deviation from the center point (0.5, 0.5) indicating an asymmetry in nucleotide usage that may reflect selection, mutational bias, or both [12]. In the present study, all genes were biased toward the Q4 quadrant, with T3 > A3 and G3 > C3, indicating a consistent asymmetry in third-codon-position composition across all six species. Similar Q4 biases have been observed in the chloroplast genomes of *Toxicodendron vernicifluum* and *Zygophyllum* species [60,61]. Taken together, the non-significant GC12–GC3 correlation argues against a dominant mutational explanation, while the ENC-plot and PR2-plot are consistent with, but do not independently prove, a role for selection. Although the opposing view emphasizes context-dependent mutation [9], the weight of evidence in the present study favors natural selection as the dominant force shaping codon usage bias in six Cucurbitaceae chloroplast genomes.

### 4.3. Amino Acid Bias and Correlation Analysis of Codon Usage Parameters

In addition to the overall forces, the codon-level (amino-acid-level) distribution of the bias merits attention. The distribution of codon usage bias was not uniform across amino acid types. RSCU analysis is commonly used to evaluate the relative usage frequency of synonymous codons, with values above 1 indicating preferential usage [41]. In the present study, the eight optimal codons shared by all six species were distributed as follows: four encoded nonpolar hydrophobic amino acids (Ile, Ala, Gly, Phe), two encoded polar uncharged amino acids (Thr, Gln), one encoded a basic positively charged amino acid (Arg), and one encoded an acidic amino acid (Asp). Similar patterns have been observed in the chloroplast genomes of *Fagopyrum* [62] and Halimedineae [63], where optimal codons also predominantly encoded uncharged amino acids.

Correlation analysis further supports this interpretation. Pearson correlation analysis is commonly used to examine the relationships among codon usage parameters [46]. In the present study, ENC showed a highly significant positive correlation with GC3 (*r* = 0.2584, *p* < 0.001) [11], a highly significant negative correlation with GC2 (*r* = −0.2773, *p* < 0.001), and no significant correlation with GC1 (*r* = 0.0692, *p* = 0.2836). The highly significant positive correlations between GC1 and GC2 indicate that the first two codon positions are relatively conserved, likely constrained by amino acid physicochemical properties. Meanwhile, GC3 showed no significant correlation with GC1 but was significantly positively correlated with GC2, indicating that the third codon position is not fully independent of the first two positions. Comparable correlations have been reported in oil-tea *Camellia* chloroplast genomes. Chen et al. (2023) [64] analyzed 13 oil-tea *Camellia* samples from South China and found that GC3 was not correlated with GC1 and GC2 but was extremely significantly correlated with ENC. The distinct behavior of GC3 relative to the other codon positions is consistent with the view that the third codon position is subject to weaker structural constraints and is more responsive to mutational and selective forces [1].

### 4.4. Cross-Lineage Consistency of A/U Preference and Possible Mechanisms

The A/U-ending preference observed in this study has also been reported in other Cucurbitaceae chloroplast genomes. Zhang et al. (2018) [16] conducted comparative analyses of chloroplast genomes across Cucurbitaceae species and found that optimal codons similarly showed a preference for A/U endings. More recently, Jia et al. (2026) [21] analyzed 12 Cucurbita chloroplast genomes and identified 30 codons with RSCU values greater than 1.00, further supporting the prevalence of A/U-ending preferences within the family. Beyond Cucurbitaceae, this pattern appears widespread across angiosperm chloroplast genomes. Similar observations have been made in *Chloranthus* [2], *Dryas octopetala* [8], and other plants [65,66,67,68], where optimal codons also predominantly ended with A or U. The prevalence of A/U-ending preferences across distantly related lineages suggests that this feature may be linked to the evolutionary origin of chloroplasts. Chloroplasts are derived from cyanobacteria through endosymbiosis, and their translation systems have retained several prokaryotic characteristics [69]. In bacteria, A/U-ending codons generally correspond to more abundant tRNAs and support higher translation efficiency [6,70]. The A/U-ending tendency observed here, six of the eight shared optimal codons ending with A or U, is therefore in line with the view that translational selection has left detectable signatures in chloroplast codon usage patterns [69,71].

### 4.5. Domestication History and Evolutionary Scale Shape Codon Usage Patterns

The six species examined in this study, representing different genera of Cucurbitaceae, are all long-term cultivated crops, and their codon usage patterns are largely conserved among the species examined. In contrast, the genus *Rhododendron*, the largest woody plant genus in the Northern Hemisphere, has undergone rapid radiation, resulting in substantial genetic divergence among species, and differentiation in codon usage bias has been observed among its subgenera [72]. This contrast may reflect differences in evolutionary timescale and taxonomic depth: the radiation of Rhododendron has accumulated sufficient differentiation signals within the genus, whereas the six Cucurbitaceae species examined here show relatively conserved plastid codon usage. Whether this conservation reflects phylogenetic constraint, a shared cultivation history, or both cannot be distinguished from the present dataset, which includes only one cultivated accession per species and no wild relatives. Zhang et al. (2018) [16] also noted that codon usage patterns in Cucurbitaceae chloroplast genomes are relatively conserved at the family level, which is consistent with our observation. Phylogenetic analysis revealed divergent branching orders among the six species; however, codon usage patterns remained consistent across all six species, with no gradient variation associated with the divergence order. Future studies incorporating wild accessions and cultivated-versus-wild comparisons are needed to test whether domestication has contributed to the observed patterns.

### 4.6. Application Value, Methodological Contribution, and Limitations

The eight codons shared by all six species (ACT, ATT, CAA, CGT, GAC, GCT, GGT, and TTC) represent candidate preferred codons that may inform codon optimization of exogenous genes in chloroplast genetic engineering of the six Cucurbitaceae species examined and their close relatives. However, because these codons were identified from RSCU and ENC analyses alone, we emphasize that they are candidates rather than a direct prescription for improving transgene translation; chloroplast translation efficiency also depends on tRNA availability [6,70], transcript context, mRNA secondary structure, codon-pair context, and gene expression level [71]. Replacing exogenous gene codons with host-preferred codons remains a commonly used strategy [1], but experimental validation or independent tRNA/expression-based support would be needed before firm engineering recommendations can be made [14]. Methodologically, this study establishes a Python-based analytical pipeline covering CDS extraction and filtering, codon usage bias analysis, and visualization following sequence downloading from the NCBI database. CodonW was employed solely for ENC calculation to ensure comparability with previous studies.

However, this study is based on a single reference plastome from each of six cultivated species, and the findings should be interpreted as applying specifically to these six accessions. Whether the observed patterns extend to other tribes within Cucurbitaceae (e.g., Siraitieae, Thladiantheae), to wild congeners, or to multiple accessions within the same species remains to be investigated. Future studies incorporating broader taxon sampling and multiple accessions per species, combined with nuclear genomic data, are needed to assess whether the patterns reported here hold at the family level.

## 5. Conclusions

This study systematically analyzed codon usage bias in the chloroplast genomes of six Cucurbitaceae species. Optimal codons were identified independently within each species; eight of them, ACT, ATT, CAA, CGT, GAC, GCT, GGT, and TTC, are shared by all six species, and species-specific optimal codons were also detected. The shared set indicates that codon usage is largely conserved among the six cultivated Cucurbitaceae species examined here. The neutrality plot analysis revealed non-significant GC12-GC3 correlations across all six species, arguing against a dominant role for mutation pressure, while the ENC-plot and PR2-plot provide complementary descriptive evidence consistent with a role for natural selection. Correlation analysis revealed a highly significant positive correlation between ENC and GC3 (*r* = 0.2584, *p* < 0.001), and a highly significant negative correlation between ENC and GC2 (*r* = −0.2773, *p* < 0.001). The eight shared candidate preferred codons provide a preliminary reference for codon optimization of exogenous genes in chloroplast genetic engineering of these six species and their close relatives, pending experimental validation.

## Figures and Tables

**Table 1:** Summary statistics of codon usage parameters in chloroplast genomes of six Cucurbitaceae species.

Species	Gene Count	Mean ENC	ENC Range	Mean GC1 (%)	Mean GC2 (%)	Mean GC3 (%)	Mean GCall (%)
*C. lanatus*	41	47.24	37.88–55.41	46.85	39.45	28.09	38.13
*C. melo*	40	47.01	37.26–55.16	46.71	39.37	28.09	38.06
*C. sativus*	41	47.55	37.96–56.45	46.77	39.53	28.36	38.22
*C. moschata*	39	47.48	38.77–55.41	46.70	39.60	28.25	38.19
*B. hispida*	41	47.18	38.03–55.41	46.89	39.37	28.01	38.09
*M. charantia*	40	47.36	35.05–56.84	46.72	39.43	27.84	38.00

**Table 2:** Four representative chloroplast genes used for phylogenetic analysis.

Gene	Full Name	Function
*atpB*	ATP synthase subunit beta	Beta subunit of ATP synthase; involved in photosynthetic energy synthesis [49]
*matK*	Maturase K	Maturase K; involved in group II intron splicing; relatively high evolutionary rate [50,51]
*ndhF*	NADH dehydrogenase subunit F	Subunit F of NADH dehydrogenase; involved in respiration; moderate evolutionary rate [52,53]
*rbcL*	Ribulose-1,5-bisphosphate carboxylase/oxygenase large subunit	Large subunit of Rubisco; key enzyme in photosynthetic carbon fixation; the most commonly used marker gene in plant phylogenetics [52,54]

**Table 3:** Optimal codons identified independently within each species in the six Cucurbitaceae species.

Species	Optimal Count	Optimal Codons	Unique to Species
*C. lanatus*	14	AAA, ACC, ACT, ATT, CAA, CAC, CGT, CTA, GAC, GCT, GGT, TCT, TTA, TTC	None
*C. melo*	17	AAA, ACC, ACT, ATT, CAA, CGA, CGT, CTA, GAC, GCT, GGT, TCG, TCT, TGT, TTA, TTC, TTG	TCG, TGT, TTG
*C. sativus*	12	AAC, ACT, ATT, CAA, CCG, CGT, CTT, GAC, GCT, GGT, GTT, TTC	AAC, CCG, CTT, GTT
*C. moschata*	15	AAA, AAT, ACT, ATT, CAA, CAC, CGA, CGT, GAC, GCT, GGT, TAC, TCT, TTA, TTC	AAT, TAC
*B. hispida*	14	ACC, ACT, ATT, CAA, CAC, CGA, CGT, GAC, GCT, GGT, TCA, TCT, TTA, TTC	TCA
*M. charantia*	10	ACT, AGT, ATT, CAA, CGT, GAC, GCT, GGT, TTA, TTC	AGT

Note: Red underlines indicate the optimal codons shared by all six Cucurbitaceae species.

## Data Availability

The chloroplast genome sequences analyzed in this study are publicly available in the NCBI RefSeq database (https://www.ncbi.nlm.nih.gov/refseq/). The custom Python scripts used for data analysis and the supporting datasets are included in the Supplementary Materials. Further inquiries can be directed to the corresponding author.

## Supplementary Materials

The following supporting information can be downloaded at: https://www.mdpi.com/article/10.3390/genes17091095/s1, Figure S1. Circular genome maps of the six chloroplast genomes. Figure S2. RSCU heatmap of codon usage across six species (high-resolution version). Figure S3. Per-species and gene-level correlation heatmaps. Figure S4. Cross-species consistency of effect sizes (forest plot). Figure S5. Optimal codon cutoff sensitivity (group size and ΔRSCU). Figure S6. Bootstrap stability of shared candidate preferred codons (B = 1000). Figure S7. Filter sensitivity: three nested sets (A, B, S0). Figure S8. Shared codons across six species under different filters. Figure S9. Two-dimensional filter sweep (start-codon rule × length threshold). Table S1. RSCU values of each codon in six Cucurbitaceae species. Table S2. GC3 content and ENC for each gene in six species. Table S3. Coordinates and quadrant assignments in the PR2-plot analysis. Table S4. GC content at each codon position for the neutrality-plot analysis. Table S5. Identification of candidate preferred codons and high/low gene sets. Table S6. Sensitivity analysis of the CDS start-codon filter. Table S7. Codon-usage parameter correlations: effect-size consistency and full results. Table S8. Stability of candidate preferred codons: bootstrap, threshold sweep, leave-one-out. Table S9. CDS filter sensitivity: three nested sets and excluded-gene details. Table S10. Two-dimensional filter sweep: start-codon rule × minimum CDS length. Supplementary Code: Computational Workflow and Source Code.

## References

[1] Parvathy S.T., Udayasuriyan V., Bhadana V. (2022). Codon Usage Bias. Mol. Biol. Rep..

[2] Zhang J., Feng M. (2025). Analysis of the Codon Usage Bias Pattern in the Chloroplast Genomes of *Chloranthus* Species (Chloranthaceae). Genes.

[3] Hu Q., Wu J., Fan C., Luo Y., Liu J., Deng Z., Li Q. (2024). Comparative Analysis of Codon Usage Bias in the Chloroplast Genomes of Eighteen Ampelopsideae Species (Vitaceae). BMC Genom. Data.

[4] Yang X., Wang Y., Gong W., Li Y. (2024). Comparative Analysis of the Codon Usage Pattern in the Chloroplast Genomes of Gnetales Species. Int. J. Mol. Sci..

[5] Li Y., Hu X., Xiao M., Huang J., Lou Y., Hu F., Fu X., Li Y., He H., Cheng J. (2023). An Analysis of Codon Utilization Patterns in the Chloroplast Genomes of Three Species of Coffea. BMC Genom. Data.

[6] Ikemura T. (1985). Codon Usage and tRNA Content in Unicellular and Multicellular Organisms. Mol. Biol. Evol..

[7] Sharp P.M., Li W.H. (1987). The Codon Adaptation Index—A Measure of Directional Synonymous Codon Usage Bias, and Its Potential Applications. Nucleic Acids Res..

[8] Ling L., Zhang S., Yang T. (2024). Analysis of Codon Usage Bias in Chloroplast Genomes of *Dryas octopetala* Var. *asiatica* (Rosaceae). Genes.

[9] Morton B.R. (2022). Context-Dependent Mutation Dynamics, Not Selection, Explains the Codon Usage Bias of Most Angiosperm Chloroplast Genes. J. Mol. Evol..

[10] Sueoka N. (1988). Directional Mutation Pressure and Neutral Molecular Evolution. Proc. Natl. Acad. Sci. USA.

[11] Wright F. (1990). The “effective Number of Codons” Used in a Gene. Gene.

[12] Sueoka N. (1995). Intrastrand Parity Rules of DNA Base Composition and Usage Biases of Synonymous Codons. J. Mol. Evol..

[13] Sueoka N. (1999). Two Aspects of DNA Base Composition: G+C Content and Translation-Coupled Deviation from Intra-Strand Rule of A = T and G = C. J. Mol. Evol..

[14] Daniell H., Lin C.-S., Yu M., Chang W.-J. (2016). Chloroplast Genomes: Diversity, Evolution, and Applications in Genetic Engineering. Genome Biol..

[15] Wang H., Wang Z., Liu Z., Smagghe G., Zhao X., Li D., Gai Y. (2026). Integrative Chloroplast Genomics of Polygonaceae: Evolutionary Dynamics, Codon Optimization, and Phylogenetic Resolution. BMC Plant Biol..

[16] Zhang X., Zhou T., Yang J., Sun J., Ju M., Zhao Y., Zhao G. (2018). Comparative Analyses of Chloroplast Genomes of Cucurbitaceae Species: Lights into Selective Pressures and Phylogenetic Relationships. Molecules.

[17] Zhang P., Xu W., Lu X., Wang L. (2021). Analysis of Codon Usage Bias of Chloroplast Genomes in Gynostemma Species. Physiol. Mol. Biol. Plants.

[18] Zhao Y., Zhang X., Zhou T., Chen X., Ding B. (2023). Complete Chloroplast Genome Sequence of *Gynostemma guangxiense*: Genome Structure, Codon Usage Bias, and Phylogenetic Relationships in Gynostemma (Cucurbitaceae). Braz. J. Bot..

[19] Mu J.-J., Zhang J.-S. (2025). Codon Usage Bias Analysis in the Chloroplast Genome of *Actinostemma tenerum* (Cucurbitaceae). Curr. Issues Mol. Biol..

[20] Song W., Chen Z., He L., Feng Q., Zhang H., Du G., Shi C., Wang S. (2022). Comparative Chloroplast Genome Analysis of Wax Gourd (*Benincasa hispida*) with Three Benincaseae Species, Revealing Evolutionary Dynamic Patterns and Phylogenetic Implications. Genes.

[21] Jia X., Wang P., Zhou C., Amanullah S., Wu F., Zeng C., Zhang P., Zhu Q. (2026). Comparative Complete Chloroplast Genome Analysis of Cucurbita (Cucurbitaceae) Species Revealed Insights into Phylogenetic Evolution, Adaptive Pressure, and Lineage Diversification. Front. Plant Sci..

[22] Yildirim E. (2026). Introductory Chapter: The Scientific and Agricultural Importance of Cucurbitaceae. Cucurbitaceae Crops—Breeding, Production, Current Research and Developments.

[23] Borecka M., Karaś M. (2025). A Comprehensive Review of the Nutritional and Health-Promoting Properties of Edible Parts of Selected Cucurbitaceae Plants. Foods.

[24] Mukherjee P.K., Singha S., Kar A., Chanda J., Banerjee S., Dasgupta B., Haldar P.K., Sharma N. (2022). Therapeutic Importance of Cucurbitaceae: A Medicinally Important Family. J. Ethnopharmacol..

[25] Marcelis L.F.M., Heuvelink E., Goudriaan J. (1998). Modelling Biomass Production and Yield of Horticultural Crops: A Review. Sci. Hortic..

[26] Xu P., Zhao C., Li S., Li S., Li A., Zhao J., Ma A., Wang Q., Guo D., Zhou J. (2025). Gene Editing Tools Promote the Development of Chloroplast Gene Engineering. Curr. Plant Biol..

[27] Caddell D., Langenfeld N.J., Eckels M.J., Zhen S., Klaras R., Mishra L., Bugbee B., Coleman-Derr D. (2023). Photosynthesis in Rice Is Increased by CRISPR/Cas9-Mediated Transformation of Two Truncated Light-Harvesting Antenna. Front. Plant Sci..

[28] Hu J., Yao J., Lu J., Liu W., Zhao Z., Li Y., Jiang L., Zha L. (2024). The Complete Chloroplast Genome Sequences of Nine Melon Varieties (*Cucumis melo* L.): Lights into Comparative Analysis and Phylogenetic Relationships. Front. Genet..

[29] Shi C., Wang S., Zhao F., Peng H., Xiang C.-L. (2017). Full Chloroplast Genome Assembly of 11 Diverse Watermelon Accessions. Front. Genet..

[30] Guan J., Miao H., Zhang Z., Dong S., Zhou Q., Liu X., Beckles D.M., Gu X., Huang S., Zhang S. (2024). A Near-Complete Cucumber Reference Genome Assembly and Cucumber-DB, a Multi-Omics Database. Mol. Plant.

[31] Cui J., Yang Y., Luo S., Wang L., Huang R., Wen Q., Han X., Miao N., Cheng J., Liu Z. (2020). Whole-Genome Sequencing Provides Insights into the Genetic Diversity and Domestication of Bitter Gourd (*Momordica* Spp.). Hortic. Res..

[32] Niu Y., Luo Y., Wang C., Liao W. (2021). Deciphering Codon Usage Patterns in Genome of *Cucumis sativus* in Comparison with Nine Species of Cucurbitaceae. Agronomy.

[33] Schaefer H., Renner S.S. (2011). Phylogenetic Relationships in the Order Cucurbitales and a New Classification of the Gourd Family (Cucurbitaceae). Taxon.

[34] Sayers E.W., Cavanaugh M., Clark K., Pruitt K.D., Schoch C.L., Sherry S.T., Karsch-Mizrachi I. (2022). GenBank. Nucleic Acids Res..

[35] Greiner S., Lehwark P., Bock R. (2019). OrganellarGenomeDRAW (OGDRAW) Version 1.3.1: Expanded Toolkit for the Graphical Visualization of Organellar Genomes. Nucleic Acids Res..

[36] Tillich M., Lehwark P., Pellizzer T., Ulbricht-Jones E.S., Fischer A., Bock R., Greiner S. (2017). GeSeq—Versatile and Accurate Annotation of Organelle Genomes. Nucleic Acids Res..

[37] Cock P.J.A., Antao T., Chang J.T., Chapman B.A., Cox C.J., Dalke A., Friedberg I., Hamelryck T., Kauff F., Wilczynski B. (2009). Biopython: Freely Available Python Tools for Computational Molecular Biology and Bioinformatics. Bioinformatics.

[38] Wang Z., Cai Q., Wang Y., Li M., Wang C., Wang Z., Jiao C., Xu C., Wang H., Zhang Z. (2022). Comparative Analysis of Codon Bias in the Chloroplast Genomes of Theaceae Species. Front. Genet..

[39] Kuroda H., Suzuki H., Kusumegi T., Hirose T., Yukawa Y., Sugiura M. (2007). Translation of psbC mRNAs Starts from the Downstream GUG, Not the Upstream AUG, and Requires the Extended Shine–Dalgarno Sequence in Tobacco Chloroplasts. Plant Cell Physiol..

[40] Hirose T., Ideue T., Wakasugi T., Sugiura M. (1999). The Chloroplast infA Gene with a Functional UUG Initiation Codon. FEBS Lett..

[41] Sharp P.M., Tuohy T.M., Mosurski K.R. (1986). Codon Usage in Yeast: Cluster Analysis Clearly Differentiates Highly and Lowly Expressed Genes. Nucleic Acids Res..

[42] Osawa S., Jukes T.H., Watanabe K., Muto A. (1992). Recent Evidence for Evolution of the Genetic Code. Microbiol. Rev..

[43] Jukes T.H., Osawa S. (1993). Evolutionary Changes in the Genetic Code. Comp. Biochem. Physiol. B.

[44] Tamura K., Stecher G., Kumar S. (2021). MEGA11: Molecular Evolutionary Genetics Analysis Version 11. Mol. Biol. Evol..

[45] Felsenstein J. (1981). Evolutionary Trees from DNA Sequences: A Maximum Likelihood Approach. J. Mol. Evol..

[46] Pearson K. (1896). Mathematical Contributions to the Theory of Evolution. III. Regression, Heredity, and Panmixia. Philos. Trans. R. Soc. A.

[47] Kathriarachchi H., Hoffmann P., Samuel R., Wurdack K.J., Chase M.W. (2005). Molecular Phylogenetics of Phyllanthaceae Inferred from Five Genes (Plastid atpB, matK, 3′ndhF, rbcL, and Nuclear PHYC). Mol. Phylogenet. Evol..

[48] Chen Z., Yang T., Lin L., Lu L., Li H.-L., Sun M., Liu B., Chen M., Niu Y., Ye J. (2016). Tree of Life for the Genera of Chinese Vascular Plants. J. Syst. Evol..

[49] Hoot S.B., Culham A., Crane P.R. (1995). The Utility of atpB Gene Sequences in Resolving Phylogenetic Relationships: Comparison with rbcL and 18S Ribosomal DNA Sequences in the Lardizabalaceae. Ann. Mo. Bot. Gard..

[50] Johnson L. (1994). matK DNA Sequences and Phylogenetic Reconstruction in Saxifragaceae s. Str. Syst. Bot..

[51] Hilu K.W., Borsch T., Müller K., Soltis D.E., Soltis P.S., Savolainen V., Chase M.W., Powell M.P., Alice L.A., Evans R. (2003). Angiosperm Phylogeny Based on matK Sequence Information. Am. J. Bot..

[52] Olmstead R.G., Sweere J.A. (1994). Combining Data in Phylogenetic Systematics: An Empirical Approach Using Three Molecular Data Sets in the Solanaceae. Syst. Biol..

[53] Kim K.J., Jansen R.K. (1995). ndhF Sequence Evolution and the Major Clades in the Sunflower Family. Proc. Natl. Acad. Sci. USA.

[54] Chase M., Olmstead R., Morgan D., Les D., Mishler B., Duvall M., Price R., Hills H., Kron K., Rettig J. (1993). Phylogenetics of Seed Plants: An Analysis of Nucleotide Sequences from the Plastid Gene rbcL. Ann. Mo. Bot. Gard..

[55] Mu Z.-Q., Zhang X., Yan J.-J., Chen B.-P., Wang H.-W. (2026). Analysis of Chloroplast Genome Characteristics and Codon Usage Bias of *Styphnolobium japonicum* f. *oligophyllum*. Curr. Issues Mol. Biol..

[56] Hu X., Li Y., Meng F., Duan Y., Sun M., Yang S., Liu H. (2024). Analysis of Chloroplast Genome Characteristics and Codon Usage Bias in 14 Species of Annonaceae. Funct. Integr. Genom..

[57] Sheng J., She X., Liu X., Wang J., Hu Z. (2021). Comparative Analysis of Codon Usage Patterns in Chloroplast Genomes of Five Miscanthus Species and Related Species. PeerJ.

[58] Luo Y., Luo W., Zhao T., Yang J., Yuan L., Zhang P., Gong Z., Li H., Sima Y., Xu T. (2025). The Complete Chloroplast Genomes of Three Manglietia Species and Phylogenetic Insight into the Genus Manglietia Blume. Curr. Issues Mol. Biol..

[59] Li G., Zhang L., Xue P., Zhu M. (2023). Comparative Analysis on the Codon Usage Pattern of the Chloroplast Genomes in Malus Species. Biochem. Genet..

[60] Zhu Y., Feng H., Yang X., Xu R., Wang C., Yu J., Zong D. (2026). Analysis and Comparison of Chloroplast Genome Codon Usage Patterns in Five Accessions of *Toxicodendron vernicifluum*. BMC Genom..

[61] Ling Z. (2024). Analysis of Codon Preference in Chloroplast Genomes of Four Zygophyllum Species. Acta Bot. Boreali-Occident. Sin..

[62] Liu Q., Li S., He D., Liu J., He X., Lin C., Li J., Huang Z., Huang L., Nie G. (2025). Comparative Analysis of Codon Usage Patterns in the Chloroplast Genomes of Fagopyrum Species. Agronomy.

[63] Fang J., Hu Y., Hu Z. (2024). Comparative Analysis of Codon Usage Patterns in 16 Chloroplast Genomes of Suborder Halimedineae. BMC Genom..

[64] Chen J., Ma W., Hu X., Zhou K. (2023). Synonymous Codon Usage Bias in the Chloroplast Genomes of 13 Oil-Tea Camellia Samples from South China. Forests.

[65] Chen H., Zhang J. (2025). Chloroplast Genome Evolution and Codon Usage In the Medicinal Plant *Pothos chinensis* (Araceae). Genes.

[66] Geng X., Xue Y., Wang H., Tu H., Jing R., Li Z., Gai Y. (2026). Comprehensive Analysis of Chloroplast Genome Evolution in Poaceae: Codon Usage Patterns, Selection Pressures, and Phylogenomic Relationships. BMC Genom..

[67] Li X., Mo X., Wang D. (2025). Phylogeny and Evolutionary Dynamics of the Rubia Genus Based on the Chloroplast Genome of *Rubia tibetica*. Sci. Rep..

[68] Singh N.K., Singh B.K., Gidhi A., Srivastava H., Pandey A., Kumar S., Pattanayak A., Bhadana V.P., Rakshit S., Tribhuvan K.U. (2026). Chloroplast Genome Sequencing in Winged Bean (*Psophocarpus tetragonolobus* L.) and Comparative Analysis with Other Legumes. BMC Plant Biol..

[69] Morton B.R. (1998). Selection on the Codon Bias of Chloroplast and Cyanelle Genes in Different Plant and Algal Lineages. J. Mol. Evol..

[70] Novoa E.M., Ribas de Pouplana L. (2012). Speeding with Control: Codon Usage, tRNAs, and Ribosomes. Trends Genet..

[71] Fages-Lartaud M., Hundvin K., Hohmann-Marriott M.F. (2022). Mechanisms Governing Codon Usage Bias and the Implications for Protein Expression in the Chloroplast of *Chlamydomonas reinhardtii*. Plant J..

[72] Shen J., Li X., Li M., Cheng H., Huang X., Jin S. (2022). Characterization, Comparative Phylogenetic, and Gene Transfer Analyses of Organelle Genomes of *Rhododendron* × *Pulchrum*. Front. Plant Sci..

