# Supplementary material for: Codon Usage Bias Analysis of Chloroplast Genomes in Six Cucurbitaceae Species
Source: Genes (Basel). 2026 Sep 11;17(9):1095. doi: 10.3390/genes17091095 (PMC13606370; doi:10.3390/genes17091095)
Supplement: Supplementary file 1 [file genes-17-01095-s001.zip › Supplementary_Code.pdf]

Codon Usage Bias Analysis of Chloroplast Genomes in Six Cucurbitaceae Species

Computational Workflow and Source Code

Manuscript: genes-4534662 | Journal: Genes (MDPI)

Yongjie Xia [1] and **Zhuoran Huang [1, 2, *]**

[1] College of Life Sciences, Huaibei Normal University, Huaibei 235000, China; 18805601148@163.com (Y.X.)
[2] Anhui Province Key Laboratory of Pollutant Sensitive Materials and Environmental Remediation, Huaibei Normal University, Huaibei 235000, China
* Correspondence: huangzr@chnu.edu.cn

1. Overview

Complete computational workflow and full source code for codon usage bias analysis of chloroplast genomes in six Cucurbitaceae species. 18 scripts covering CDS extraction, filtering, RSCU/ENC, optimal codons, ENC-plot, neutrality plot, PR2-plot, correlation, phylogenetic preparation, and four sensitivity analyses. All scripts parameterised and verified to reproduce manuscript data.

2. Software and Dependencies

Software	Version	Purpose
Python	3.11.9	Primary language
Biopython	1.87	GenBank parsing, CDS extraction
pandas	2.3.3	Tabular data, Excel I/O
NumPy	2.3.5	Numerical computation
SciPy	1.17.1	Correlation, FDR control
Matplotlib	3.11.0	All figures
Seaborn	0.13.2	Clustered heatmaps
openpyxl	3.1.5	Excel I/O
CodonW	1.4.2	ENC calculation (bundled)
MEGA11	11	Alignment, ML trees

3. Data Sources

Species	Common name	RefSeq ID	Tribe
Citrullus lanatus	Watermelon	NC_032008	Benincaseae
Cucumis melo	Melon	NC_057560	Benincaseae
Cucumis sativus	Cucumber	NC_007144	Benincaseae
Cucurbita moschata	Pumpkin	NC_036506	Cucurbiteae
Benincasa hispida	Wax gourd	NC_056352	Benincaseae
Momordica charantia	Bitter gourd	NC_036807	Momordiceae

4. Analysis Workflow and Source Code

Steps 01-11 produce manuscript results. Steps 12-15 are sensitivity analyses added in response to Reviewer 2.

Step 01. CDS Extraction

Extract all annotated CDS features from GenBank files using Biopython.

Input: GenBank (.gb) files

Output: species_CDS.fasta (106-112 per species)

Method: Biopython SeqIO; location-aware extraction

Source Code (step01_extract_cds.py):

```
from Bio import SeqIO
import os
# ---------------------------------------------------------------------------
# Repository configuration (ADDED for public release - see CHANGES.md)
# Only the hard-coded local paths were parameterised; no algorithm, threshold,
# formula, sort order or output content was modified.
# CUB_DATA_DIR : directory holding the input data (default:../data)
# CUB_OUT_DIR : directory for outputs (default:../output)
# ---------------------------------------------------------------------------
import os as _os
_HERE = _os.path.dirname(_os.path.abspath(__file__))
DATA_DIR = _os.environ.get("CUB_DATA_DIR", _os.path.normpath(_os.path.join(_HERE,
_os.pardir, "data")))
OUT_DIR = _os.environ.get("CUB_OUT_DIR", _os.path.normpath(_os.path.join(_HERE,
_os.pardir, "output")))
_os.makedirs(OUT_DIR, exist_ok=True)
# ---------------------------------------------------------------------------

def extract_cds(gb_file, output_file):
 count = 0
 with open(output_file, 'w', encoding='utf-8') as out:
 for record in SeqIO.parse(gb_file, 'genbank'):
 for feature in record.features:
 if feature.type == 'CDS':
 gene = feature.qualifiers.get('gene', ['unknown'])[0]
 seq = feature.location.extract(record.seq)
 out.write(f'>{gene}\n{seq}\n')
```

```
 count += 1
 print(f"[OK] Extracted {count} CDS sequences!")
 print(f"[FILE] Output file: {output_file}")

if __name__ == '__main__':
 # Update the two paths below!
 input_file = os.path.join(DATA_DIR, "6_bitter melon.gb")
 output_file = os.path.join(DATA_DIR, "bitter melon_CDS.fasta")
 extract_cds(input_file, output_file)
```

Step 02. CDS Filtering

Three-step filter: ATG start, length >= 300 bp, remove duplicates.

Input: species_CDS.fasta

Output: species_CDS_filtered.fasta (39-41 per species, 242 total)

Method: ATG + >=300bp + deduplicate

Source Code (step02_filter_cds.py):

```
from Bio import SeqIO
import os
# ---------------------------------------------------------------------------
# Repository configuration (ADDED for public release - see CHANGES.md)
# Only the hard-coded local paths were parameterised; no algorithm, threshold,
# formula, sort order or output content was modified.
# CUB_DATA_DIR : directory holding the input data (default:../data)
# CUB_OUT_DIR : directory for outputs (default:../output)
# ---------------------------------------------------------------------------
import os as _os
_HERE = _os.path.dirname(_os.path.abspath(__file__))
DATA_DIR = _os.environ.get("CUB_DATA_DIR", _os.path.normpath(_os.path.join(_HERE,
_os.pardir, "data")))
OUT_DIR = _os.environ.get("CUB_OUT_DIR", _os.path.normpath(_os.path.join(_HERE,
_os.pardir, "output")))
_os.makedirs(OUT_DIR, exist_ok=True)
# ---------------------------------------------------------------------------

def filter_cds_fasta(input_fasta, output_fasta, min_length=300):
 """
 Three-step filtering of extracted CDS FASTA:
 1. ATG start
 2. >= min_length bp
 3. Remove duplicates (keep first occurrence)
 """
 count_total = 0
 count_atg = 0
 count_length = 0
 count_final = 0
 seen_genes = set()

 with open(output_fasta, 'w', encoding='utf-8') as out:
 for record in SeqIO.parse(input_fasta, 'fasta'):
 count_total += 1
 gene = record.id

 seq = str(record.seq)
 if not seq.startswith('ATG'):
```

```
    continue
    count_atg += 1

    # Filter 2: length >= min_length
    if len(seq) < min_length:
    continue
    count_length += 1

    # Filter 3: remove duplicates
    if gene in seen_genes:
    continue
    seen_genes.add(gene)

    out.write(f'>{gene}\n{seq}\n')
    count_final += 1

    print(f"[STATS] {os.path.basename(input_fasta)} Filter statistics:")
    print(f" Total CDS: {count_total}")
    print(f" Pass ATG filter: {count_atg}")
    print(f" Pass length filter(>={min_length}bp): {count_length}")
    print(f" Final after dedup: {count_final}")
    print(f" [OK] Output file: {output_fasta}")

if __name__ == '__main__':
    input_fasta = os.path.join(DATA_DIR, "bitter melon_CDS.fasta")
    output_fasta = os.path.join(DATA_DIR, "bitter melon_CDS_filtered.fasta")
    filter_cds_fasta(input_fasta, output_fasta, min_length=300)
```

Step 03. RSCU Calculation

Relative Synonymous Codon Usage for six species.

Input: species_CDS_filtered.fasta

Output: species_codon_usage.txt

Method: Standard genetic code; RSCU formula

Source Code (step03_calc_rscu.py):

```
from Bio import SeqIO
import os
import sys
from collections import defaultdict
# ---------------------------------------------------------------------------
# Repository configuration (ADDED for public release - see CHANGES.md)
# Only the hard-coded local paths were parameterised; no algorithm, threshold,
# formula, sort order or output content was modified.
# CUB_DATA_DIR : directory holding the input data (default:../data)
# CUB_OUT_DIR : directory for outputs (default:../output)
# ---------------------------------------------------------------------------
import os as _os
_HERE = _os.path.dirname(_os.path.abspath(__file__))
DATA_DIR = _os.environ.get("CUB_DATA_DIR", _os.path.normpath(_os.path.join(_HERE,
_os.pardir, "data")))
OUT_DIR = _os.environ.get("CUB_OUT_DIR", _os.path.normpath(_os.path.join(_HERE,
_os.pardir, "output")))
_os.makedirs(OUT_DIR, exist_ok=True)
# ---------------------------------------------------------------------------

# Standard genetic code table (DNA -> AA)
```

```
CODON_TABLE = {
 'TTT': 'F', 'TTC': 'F', 'TTA': 'L', 'TTG': 'L',
 'TCT': 'S', 'TCC': 'S', 'TCA': 'S', 'TCG': 'S',
 'TAT': 'Y', 'TAC': 'Y', 'TAA': '*', 'TAG': '*',
 'TGT': 'C', 'TGC': 'C', 'TGA': '*', 'TGG': 'W',
 'CTT': 'L', 'CTC': 'L', 'CTA': 'L', 'CTG': 'L',
 'CCT': 'P', 'CCC': 'P', 'CCA': 'P', 'CCG': 'P',
 'CAT': 'H', 'CAC': 'H', 'CAA': 'Q', 'CAG': 'Q',
 'CGT': 'R', 'CGC': 'R', 'CGA': 'R', 'CGG': 'R',
 'ATT': 'I', 'ATC': 'I', 'ATA': 'I', 'ATG': 'M',
 'ACT': 'T', 'ACC': 'T', 'ACA': 'T', 'ACG': 'T',
 'AAT': 'N', 'AAC': 'N', 'AAA': 'K', 'AAG': 'K',
 'AGT': 'S', 'AGC': 'S', 'AGA': 'R', 'AGG': 'R',
 'GTT': 'V', 'GTC': 'V', 'GTA': 'V', 'GTG': 'V',
 'GCT': 'A', 'GCC': 'A', 'GCA': 'A', 'GCG': 'A',
 'GAT': 'D', 'GAC': 'D', 'GAA': 'E', 'GAG': 'E',
 'GGT': 'G', 'GGC': 'G', 'GGA': 'G', 'GGG': 'G',
}

def calculate_rscu(fasta_file, output_file):
 """
 Calculate codon usage for one species, output:
 AA, codon, count, RSCU
 """
 # Count codon occurrences
 codon_counts = defaultdict(int)
 total_codons = 0
 invalid_codons = 0

 # Read FASTA file
 for record in SeqIO.parse(fasta_file, 'fasta'):
 seq = str(record.seq).upper().replace('\n', '')
 # Split into codons
 for i in range(0, len(seq) - 2, 3):
 codon = seq[i:i+3]
 if len(codon) == 3 and codon in CODON_TABLE:
 codon_counts[codon] += 1
 total_codons += 1
 else:
 invalid_codons += 1

 if total_codons == 0:
 print(f"[WARN] {fasta_file} no valid codons, check file format")
 return

 # Group by AA, compute totals and synonymous counts
 aa_total = defaultdict(int)
 aa_codon_count = defaultdict(int) # synonymous codons per AA
 for codon, aa in CODON_TABLE.items():
 if aa != '*': # Exclude stop codons
 aa_total[aa] += codon_counts.get(codon, 0)
 aa_codon_count[aa] += 1

 # Calculate RSCU
 rscu_results = []
 for codon, aa in CODON_TABLE.items():
 if aa == '*':
 continue
 count = codon_counts.get(codon, 0)
```

```
if aa_total[aa] == 0:
rscu = 0
else:
# RSCU = (count / AA_total) / (1 / n_synonymous)
rscu = (count / aa_total[aa]) / (1 / aa_codon_count[aa])
rscu_results.append((aa, codon, count, round(rscu, 4)))

# Sort by AA order
aa_order = ['A', 'C', 'D', 'E', 'F', 'G', 'H', 'I', 'K', 'L',
'M', 'N', 'P', 'Q', 'R', 'S', 'T', 'V', 'W', 'Y']
rscu_results_sorted = sorted(rscu_results, key=lambda x: (aa_order.index(x[0]), x[1]))

with open(output_file, 'w', encoding='utf-8') as out:
out.write(f"# Codon usage bias analysis results\n")
out.write(f"# Species: {os.path.basename(fasta_file)}\n")
out.write(f"# Total codons: {total_codons}\n")
out.write(f"
for aa, codon, count, rscu in rscu_results_sorted:
out.write(f"{aa}\t{codon}\t{count}\t{rscu}\n")

print(f"[OK] Done: {os.path.basename(fasta_file)} -> Total codons {total_codons} codons")
print(f" Output file: {output_file}")

if __name__ == '__main__':
# Desktop path (update for your username)
desktop = DATA_DIR

# Six species file list
species_files = [
("watermelon_CDS_filtered.fasta", "watermelon_codon_usage.txt"),
("melon_CDS_filtered.fasta", "melon_codon_usage.txt"),
("cucumber_CDS_filtered.fasta", "cucumber_codon_usage.txt"),
("pumpkin_CDS_filtered.fasta", "pumpkin_codon_usage.txt"),
("winter melon_CDS_filtered.fasta", "winter melon_codon_usage.txt"),
("bitter melon_CDS_filtered.fasta", "bitter melon_codon_usage.txt"),
]

for input_file, output_file in species_files:
input_path = os.path.join(desktop, input_file)
output_path = os.path.join(desktop, output_file)

if not os.path.exists(input_path):
print(f"[WARN] File not found: {input_path}, skipped")
continue

calculate_rscu(input_path, output_path)

print("\n[DONE] All species codon analysis Done!")
```

Step 04. RSCU Heatmap

Clustered heatmap of RSCU values.

Input: RSCU.xlsx

Output: rscu_heatmap.pdf /.png (Figure 2A)

Method: Seaborn clustermap

Source Code (step04_rscu_heatmap.py):

```
import pandas as pd
import seaborn as sns
import matplotlib.pyplot as plt
import matplotlib as mpl
import os
# ---------------------------------------------------------------------------
# Repository configuration (ADDED for public release - see CHANGES.md)
# Only the hard-coded local paths were parameterised; no algorithm, threshold,
# formula, sort order or output content was modified.
# CUB_DATA_DIR : directory holding the input data (default:../data)
# CUB_OUT_DIR : directory for outputs (default:../output)
# ---------------------------------------------------------------------------
import os as _os
_HERE = _os.path.dirname(_os.path.abspath(__file__))
DATA_DIR = _os.environ.get("CUB_DATA_DIR", _os.path.normpath(_os.path.join(_HERE,
_os.pardir, "data")))
OUT_DIR = _os.environ.get("CUB_OUT_DIR", _os.path.normpath(_os.path.join(_HERE,
_os.pardir, "output")))
_os.makedirs(OUT_DIR, exist_ok=True)
# ---------------------------------------------------------------------------

# ==========================================
# ==========================================
mpl.rcParams['pdf.fonttype'] = 42
mpl.rcParams['ps.fonttype'] = 42
mpl.rcParams['font.family'] = 'Times New Roman'
mpl.rcParams['mathtext.fontset'] = 'stix'

# ==========================================
# Read data
# ==========================================
desktop = DATA_DIR
file_path = os.path.join(desktop, "RSCU.xlsx")
df = pd.read_excel(file_path)

# ==========================================
# ==========================================
df.set_index(['', ''], inplace=True)

species_map = {
 '': r'$\mathit{C. lanatus}$',
 '': r'$\mathit{C. melo}$',
 '': r'$\mathit{C. sativus}$',
 '': r'$\mathit{C. moschata}$',
 '': r'$\mathit{B. hispida}$',
 '': r'$\mathit{M. charantia}$'
}
df.rename(columns=species_map, inplace=True)
data = df[list(species_map.values())]

row_labels = [f"{aa}-{codon}" for aa, codon in data.index]
data.index = row_labels

# ==========================================
# ==========================================
g = sns.clustermap(
 data,
 method='average',
 metric='euclidean',
```

```
 cmap='RdBu_r',
 center=1,
 figsize=(14, 20),
 dendrogram_ratio=0.12,
 cbar_pos=(0.98, 0.3, 0.03, 0.4),
 annot=True,
 fmt='.2f',
 annot_kws={'size': 8},
 linewidths=0.2,
 linecolor='lightgray',
 cbar_kws={'label': 'RSCU value', 'shrink': 0.8}
)

# ==========================================
# ==========================================
g.ax_heatmap.set_xlabel('Species', fontsize=16)
g.ax_heatmap.set_ylabel('Codon', fontsize=16)
g.ax_heatmap.tick_params(axis='x', labelsize=14)
g.ax_heatmap.tick_params(axis='y', labelsize=8)

g.cax.set_ylabel('RSCU value', fontsize=14)
g.cax.yaxis.set_tick_params(labelsize=12)

# ==========================================
# ==========================================
output_pdf = os.path.join(desktop, "rscu_heatmap.pdf")
plt.savefig(output_pdf, dpi=300, bbox_inches='tight')
print(f"[OK] : {output_pdf}")

output_png = os.path.join(desktop, "rscu_heatmap.png")
plt.savefig(output_png, dpi=300, bbox_inches='tight')
print(f"[OK] : {output_png}")

# plt.show() # disabled: blocks unattended execution
```

Step 05. ENC-plot

ENC vs GC3 scatter with Wright's expected curve.

Input: ENC.xlsx (CodonW)

Output: ENC_plot.pdf (Figure 2C)

Method: Wright 1990 expected curve

Source Code (step05_enc_plot.py):

```
import pandas as pd
import numpy as np
import matplotlib.pyplot as plt
import matplotlib
import os
# ---------------------------------------------------------------------------
# Repository configuration (ADDED for public release - see CHANGES.md)
# Only the hard-coded local paths were parameterised; no algorithm, threshold,
# formula, sort order or output content was modified.
# CUB_DATA_DIR : directory holding the input data (default:../data)
# CUB_OUT_DIR : directory for outputs (default:../output)
# ---------------------------------------------------------------------------
import os as _os
_HERE = _os.path.dirname(_os.path.abspath(__file__))
```

```
DATA_DIR = _os.environ.get("CUB_DATA_DIR", _os.path.normpath(_os.path.join(_HERE,
_os.pardir, "data")))
OUT_DIR = _os.environ.get("CUB_OUT_DIR", _os.path.normpath(_os.path.join(_HERE,
_os.pardir, "output")))
_os.makedirs(OUT_DIR, exist_ok=True)
# ---------------------------------------------------------------------------
matplotlib.rcParams['font.sans-serif'] = ['Arial']
matplotlib.rcParams['axes.unicode_minus'] = False

# ===== Read data =====
file_path = os.path.join(DATA_DIR, "ENC.xlsx")
df = pd.read_excel(file_path)

# ===== Species colors (Nature palette) =====
colors = {
 "C. lanatus": "#E69F00",
 "C. melo": "#56B4E9",
 "C. sativus": "#009E73",
 "C. moschata": "#F0E442",
 "B. hispida": "#D55E00",
 "M. charantia": "#CC79A7",
}

# ===== Plot =====
fig, ax = plt.subplots(figsize=(10, 7))

for species, color in colors.items():
 subset = df[df['Species'] == species]
 ax.scatter(subset['GC3'], subset['ENC'],
 label=species, s=40, alpha=0.7,
 color=color, edgecolors='black', linewidth=0.5)

# ===== Expected curve =====
gc3_range = np.linspace(0.05, 0.95, 100)
enc_expected = []
for gc3 in gc3_range:
 if gc3 <= 0.5:
 enc = 2 + gc3 + 29 / (gc3**2 + (1 - gc3)**2)
 else:
 enc = 2 + (1 - gc3) + 29 / (gc3**2 + (1 - gc3)**2)
 enc_expected.append(enc)
ax.plot(gc3_range, enc_expected, 'k--', linewidth=2, label='Expected ENC curve')

ax.set_xlabel('GC3 content', fontsize=12)
ax.set_ylabel('ENC value', fontsize=12)
ax.set_title('ENC-plot of chloroplast genomes from six Cucurbitaceae species',
fontsize=14)
ax.legend(loc='upper right', fontsize=9)
ax.grid(alpha=0.3)
ax.set_xlim(0, 1.0)
ax.set_ylim(0, 70)

output_path = os.path.join(OUT_DIR, "ENC_plot.pdf")
plt.savefig(output_path, format='pdf', bbox_inches='tight')
print(f"[OK] Editable PDF saved: {output_path}")

# plt.show() # disabled: blocks unattended execution
```

Step 06. Neutrality Plot

GC1/GC2/GC3/GC12 per gene; GC12 vs GC3 regression.

Input: species_CDS_filtered.fasta

Output: neutrality_data.xlsx + plots (Figure 3)

Method: Per-position GC; linear regression

Source Code (step06_neutrality_plot.py):

```
from Bio import SeqIO
import pandas as pd
import os
# ---------------------------------------------------------------------------
# Repository configuration (ADDED for public release - see CHANGES.md)
# Only the hard-coded local paths were parameterised; no algorithm, threshold,
# formula, sort order or output content was modified.
# CUB_DATA_DIR : directory holding the input data (default:../data)
# CUB_OUT_DIR : directory for outputs (default:../output)
# ---------------------------------------------------------------------------
import os as _os
_HERE = _os.path.dirname(_os.path.abspath(__file__))
DATA_DIR = _os.environ.get("CUB_DATA_DIR", _os.path.normpath(_os.path.join(_HERE,
_os.pardir, "data")))
OUT_DIR = _os.environ.get("CUB_OUT_DIR", _os.path.normpath(_os.path.join(_HERE,
_os.pardir, "output")))
_os.makedirs(OUT_DIR, exist_ok=True)
# ---------------------------------------------------------------------------

desktop = DATA_DIR

species_files = {
 "": "watermelon_CDS_filtered.fasta",
 "": "melon_CDS_filtered.fasta",
 "": "cucumber_CDS_filtered.fasta",
 "": "pumpkin_CDS_filtered.fasta",
 "": "winter_melon_CDS_filtered.fasta",
 "": "bitter_melon_CDS_filtered.fasta",
}

def calc_gc123(seq):
 seq = seq.upper()
 codons = [seq[i:i+3] for i in range(0, len(seq)-2, 3) if len(seq[i:i+3]) == 3]
 if len(codons) < 5:
 return None, None, None
 gc1_total = 0
 gc2_total = 0
 gc3_total = 0
 for codon in codons:
 if len(codon) == 3:
 gc1_total += (codon[0] == 'G') + (codon[0] == 'C')
 gc2_total += (codon[1] == 'G') + (codon[1] == 'C')
 gc3_total += (codon[2] == 'G') + (codon[2] == 'C')
 n = len(codons)
 gc1 = gc1_total / n
 gc2 = gc2_total / n
 gc3 = gc3_total / n
 gc12 = (gc1 + gc2) / 2
 return gc1, gc2, gc3, gc12
```

```
for species, filename in species_files.items():
 filepath = os.path.join(desktop, filename)
 if not os.path.exists(filepath):
 print(f"[WARN] File not found: {filename}")
 continue

 results = []
 for record in SeqIO.parse(filepath, 'fasta'):
 gene_name = record.id
 seq = str(record.seq)
 gc1, gc2, gc3, gc12 = calc_gc123(seq)
 if gc1 is not None:
 results.append({
 '': gene_name,
 'GC1': round(gc1, 4),
 'GC2': round(gc2, 4),
 'GC3': round(gc3, 4),
 'GC12': round(gc12, 4)
 })

 if not results:
 print(f"[WARN] {species} None")
 continue

 df = pd.DataFrame(results)
 output_path = os.path.join(desktop, f"{species}_Plot.xlsx")
 df.to_excel(output_path, index=False)
 print(f"[OK] {species}: {len(results)} -> {output_path}")

print("\n[DONE] Done!")
```

Step 07. PR2-plot

Parity Rule 2: G3/(G3+C3) vs A3/(A3+T3); quadrant assignment.

Input: species_CDS_filtered.fasta

Output: PR2_data.xlsx + plot (Figure 2E)

Method: Third-position base frequencies

Source Code (step07_pr2_plot.py):

```
from Bio import SeqIO
import pandas as pd
import numpy as np
import matplotlib.pyplot as plt
import os
import matplotlib
# ---------------------------------------------------------------------------
# Repository configuration (ADDED for public release - see CHANGES.md)
# Only the hard-coded local paths were parameterised; no algorithm, threshold,
# formula, sort order or output content was modified.
# CUB_DATA_DIR : directory holding the input data (default:../data)
# CUB_OUT_DIR : directory for outputs (default:../output)
# ---------------------------------------------------------------------------
import os as _os
_HERE = _os.path.dirname(_os.path.abspath(__file__))
DATA_DIR = _os.environ.get("CUB_DATA_DIR", _os.path.normpath(_os.path.join(_HERE,
_os.pardir, "data")))
```

```
OUT_DIR = _os.environ.get("CUB_OUT_DIR", _os.path.normpath(_os.path.join(_HERE,
_os.pardir, "output")))
_os.makedirs(OUT_DIR, exist_ok=True)
# ---------------------------------------------------------------------------
matplotlib.rcParams['font.sans-serif'] = ['SimHei', 'Microsoft YaHei', 'PingFang SC',
'Arial Unicode MS']
matplotlib.rcParams['axes.unicode_minus'] = False

desktop = DATA_DIR

species_files = {
 "": "watermelon_CDS_filtered.fasta",
 "": "melon_CDS_filtered.fasta",
 "": "cucumber_CDS_filtered.fasta",
 "": "pumpkin_CDS_filtered.fasta",
 "": "winter_melon_CDS_filtered.fasta",
 "": "bitter_melon_CDS_filtered.fasta",
}

colors = {
 "": "#E69F00",
 "": "#56B4E9",
 "": "#009E73",
 "": "#F0E442",
 "": "#D55E00",
 "": "#CC79A7",
}

shapes = {
 "": "o",
 "": "s",
 "": "^",
 "": "D",
 "": "v",
 "": "*",
}

species_list = list(species_files.keys())

def calc_pr2(seq):
 seq = seq.upper()
 codons = [seq[i:i+3] for i in range(0, len(seq)-2, 3) if len(seq[i:i+3]) == 3]
 if len(codons) < 5:
 return None
 a3 = t3 = g3 = c3 = 0
 for codon in codons:
 third = codon[2]
 if third == 'A':
 a3 += 1
 elif third == 'T':
 t3 += 1
 elif third == 'G':
 g3 += 1
 elif third == 'C':
 c3 += 1
 total = a3 + t3 + g3 + c3
 if total == 0:
 return None
 return {
 'A3/(A3+T3)': a3 / (a3 + t3) if (a3 + t3) > 0 else 0,
```

```
 'G3/(G3+C3)': g3 / (g3 + c3) if (g3 + c3) > 0 else 0,
 }

plt.figure(figsize=(12, 10))
all_data = []

for species, filename in species_files.items():
 filepath = os.path.join(desktop, filename)
 if not os.path.exists(filepath):
 print(f"[WARN] File not found: {filename}")
 continue
 x_vals, y_vals = [], []
 for record in SeqIO.parse(filepath, 'fasta'):
 seq = str(record.seq)
 result = calc_pr2(seq)
 if result is None:
 continue
 x = result['G3/(G3+C3)']
 y = result['A3/(A3+T3)']
 x_vals.append(x)
 y_vals.append(y)
 all_data.append((species, x, y))
 plt.scatter(x_vals, y_vals, label=species, s=50, alpha=0.8,
 color=colors.get(species, '#888888'), marker=shapes.get(species, 'o'),
 edgecolors='black', linewidth=0.5)

plt.axhline(y=0.5, color='gray', linestyle='--', linewidth=1.5, alpha=0.6)
plt.axvline(x=0.5, color='gray', linestyle='--', linewidth=1.5, alpha=0.6)

plt.text(0.78, 0.78, 'Q1', fontsize=14, fontweight='bold', color='black', alpha=0.5)
plt.text(0.22, 0.78, 'Q2', fontsize=14, fontweight='bold', color='black', alpha=0.5)
plt.text(0.22, 0.22, 'Q3', fontsize=14, fontweight='bold', color='black', alpha=0.5)
plt.text(0.78, 0.22, 'Q4', fontsize=14, fontweight='bold', color='black', alpha=0.5)

plt.text(0.52, 0.48, '(0.5, 0.5)', fontsize=10, color='gray')

df_all = pd.DataFrame(all_data, columns=['', 'x', 'y'])
quadrants = []
for _, row in df_all.iterrows():
 if row['x'] >= 0.5 and row['y'] >= 0.5:
 q = 'Q1'
 elif row['x'] < 0.5 and row['y'] >= 0.5:
 q = 'Q2'
 elif row['x'] < 0.5 and row['y'] < 0.5:
 q = 'Q3'
 else:
 q = 'Q4'
 quadrants.append(q)
df_all[''] = quadrants

species_total = {}
for sp in species_list:
 species_total[sp] = len(df_all[df_all[''] == sp])

quad_pos = {
 'Q1': (0.65, 0.88),
 'Q2': (0.02, 0.88),
 'Q3': (0.02, 0.02),
 'Q4': (0.65, 0.02),
}
```

```
for q, (x_pos, y_pos) in quad_pos.items():
 sub = df_all[df_all[''] == q]
 q_total = len(sub)
 text_lines = [f"{q} (n={q_total})"]
 for sp in species_list:
 count = len(sub[sub[''] == sp])
 total = species_total[sp]
 if total > 0:
 pct = count / total * 100
 text_lines.append(f"{sp}: {count} ({pct:.1f}%)")
 else:
 text_lines.append(f"{sp}: 0 (0.0%)")
 text_str = "\n".join(text_lines)
 plt.text(x_pos, y_pos, text_str, transform=plt.gca().transAxes,
 fontsize=7, verticalalignment='top',
 bbox=dict(boxstyle='round', facecolor='white', alpha=0.85, edgecolor='gray'))

plt.xlim(-0.02, 1.02)
plt.ylim(-0.02, 1.02)
plt.xlabel('G3 / (G3 + C3)', fontsize=13)
plt.ylabel('A3 / (A3 + T3)', fontsize=13)
plt.title('6 PR2-plot ', fontsize=15)
plt.legend(loc='upper right', fontsize=9, framealpha=0.9)
plt.grid(alpha=0.2)

output_img = os.path.join(desktop, 'PR2_plot_.png')
plt.savefig(output_img, dpi=300, bbox_inches='tight')
print(f"[OK] : {output_img}")

output_excel = os.path.join(desktop, 'PR2_plot_.xlsx')
df_all.to_excel(output_excel, index=False)
print(f"[OK] : {output_excel}")

print("\n[STATS] :")
for q in ['Q1', 'Q2', 'Q3', 'Q4']:
 sub = df_all[df_all[''] == q]
 print(f" {q}: {len(sub)} ({len(sub)/len(df_all)*100:.1f}%)")

print("\n[STATS] CDS:")
for sp in species_list:
 count = species_total[sp]
 print(f" {sp}: {count}")

print(f"\n[STATS] : {len(df_all)}")
# plt.show() # disabled: blocks unattended execution
print("\n[DONE] Done!")
```

Step 08. Optimal Codon Identification

ENC-ranked high/low groups (10% each); optimal = RSCU>1.0 AND delta RSCU>=0.08.

Input: filtered.fasta + ENC.xlsx

Output: Optimal_Codon_Summary.xlsx (Table 3, S6)

Method: ENC ranking; delta RSCU threshold 0.08

Source Code (step08_optimal_codons.py):

```
import os
```

```
import pandas as pd
from Bio import SeqIO
from collections import defaultdict
# ---------------------------------------------------------------------------
# Repository configuration (ADDED for public release - see CHANGES.md)
# Only the hard-coded local paths were parameterised; no algorithm, threshold,
# formula, sort order or output content was modified.
# CUB_DATA_DIR : directory holding the input data (default:../data)
# CUB_OUT_DIR : directory for outputs (default:../output)
# ---------------------------------------------------------------------------
import os as _os
_HERE = _os.path.dirname(_os.path.abspath(__file__))
DATA_DIR = _os.environ.get("CUB_DATA_DIR", _os.path.normpath(_os.path.join(_HERE,
_os.pardir, "data")))
OUT_DIR = _os.environ.get("CUB_OUT_DIR", _os.path.normpath(_os.path.join(_HERE,
_os.pardir, "output")))
_os.makedirs(OUT_DIR, exist_ok=True)
# ---------------------------------------------------------------------------

desktop = DATA_DIR

# Input file
enc_file = os.path.join(desktop, "ENC.xlsx")
species_files = {
 "C. lanatus": "watermelon_CDS_filtered.fasta",
 "C. melo": "melon_CDS_filtered.fasta",
 "C. sativus": "cucumber_CDS_filtered.fasta",
 "C. moschata": "pumpkin_CDS_filtered.fasta",
 "B. hispida": "winter_melon_CDS_filtered.fasta",
 "M. charantia": "bitter_melon_CDS_filtered.fasta",
}

output_file = os.path.join(desktop, "Optimal_Codon_Summary.xlsx")

CODON_TABLE = {
 'TTT':'F','TTC':'F','TTA':'L','TTG':'L',
 'TCT':'S','TCC':'S','TCA':'S','TCG':'S',
 'TAT':'Y','TAC':'Y','TAA':'*','TAG':'*',
 'TGT':'C','TGC':'C','TGA':'*','TGG':'W',
 'CTT':'L','CTC':'L','CTA':'L','CTG':'L',
 'CCT':'P','CCC':'P','CCA':'P','CCG':'P',
 'CAT':'H','CAC':'H','CAA':'Q','CAG':'Q',
 'CGT':'R','CGC':'R','CGA':'R','CGG':'R',
 'ATT':'I','ATC':'I','ATA':'I','ATG':'M',
 'ACT':'T','ACC':'T','ACA':'T','ACG':'T',
 'AAT':'N','AAC':'N','AAA':'K','AAG':'K',
 'AGT':'S','AGC':'S','AGA':'R','AGG':'R',
 'GTT':'V','GTC':'V','GTA':'V','GTG':'V',
 'GCT':'A','GCC':'A','GCA':'A','GCG':'A',
 'GAT':'D','GAC':'D','GAA':'E','GAG':'E',
 'GGT':'G','GGC':'G','GGA':'G','GGG':'G',
}

def calc_rscu_per_gene(seq):
 seq = seq.upper()
 codons = [seq[i:i+3] for i in range(0, len(seq)-2, 3)]
 valid = [c for c in codons if c in CODON_TABLE and CODON_TABLE[c] != '*']
 if len(valid) < 10:
 return None
```

```
 aa_codon_count = defaultdict(lambda: defaultdict(int))
 aa_total = defaultdict(int)
 for c in valid:
 aa = CODON_TABLE[c]
 aa_codon_count[aa][c] += 1
 aa_total[aa] += 1

 rscu = {}
 for aa in aa_total:
 if aa == '*':
 continue
 n = len(aa_codon_count[aa])
 if n <= 1:
 continue
 total = aa_total[aa]
 for c, count in aa_codon_count[aa].items():
 rscu[c] = (count / total) * n
 return rscu

df_enc = pd.read_excel(enc_file)

all_results = []
gene_lists = []

for species, fasta_name in species_files.items():
 print(f" {species}...")
 fasta_path = os.path.join(desktop, fasta_name)
 if not os.path.exists(fasta_path):
 print(f" [WARN] File not found: {fasta_path}")
 continue

 gene_rscu = {}
 gene_seqs = {}
 for record in SeqIO.parse(fasta_path, 'fasta'):
 gene = record.id
 seq = str(record.seq)
 gene_seqs[gene] = seq
 rscu_dict = calc_rscu_per_gene(seq)
 if rscu_dict:
 gene_rscu[gene] = rscu_dict

 enc_sub = df_enc[df_enc['Species'] == species]
 enc_dict = dict(zip(enc_sub['Gene'], enc_sub['ENC']))

 common_genes = set(gene_rscu.keys()) & set(enc_dict.keys())
 if not common_genes:
 print(f" [WARN] , skipped")
 continue

 gene_enc_list = [(g, enc_dict[g]) for g in common_genes]
 gene_enc_list.sort(key=lambda x: x[1])
 n = len(gene_enc_list)
 n_high = max(1, int(n * 0.1))
 n_low = max(1, int(n * 0.1))
 high_genes = [g for g, _ in gene_enc_list[:n_high]]
 low_genes = [g for g, _ in gene_enc_list[-n_low:]]

 high_rscu = defaultdict(list)
 low_rscu = defaultdict(list)
 total_rscu = defaultdict(list)
```

```
 for gene in high_genes:
 for codon, val in gene_rscu[gene].items():
 high_rscu[codon].append(val)
 for gene in low_genes:
 for codon, val in gene_rscu[gene].items():
 low_rscu[codon].append(val)
 for gene in gene_rscu:
 for codon, val in gene_rscu[gene].items():
 total_rscu[codon].append(val)

 high_avg = {c: sum(vals)/len(vals) for c, vals in high_rscu.items()}
 low_avg = {c: sum(vals)/len(vals) for c, vals in low_rscu.items()}
 total_avg = {c: sum(vals)/len(vals) for c, vals in total_rscu.items()}

 all_codons = set(high_avg.keys()) | set(low_avg.keys()) | set(total_avg.keys())

 optimal_codons = []
 for codon in sorted(all_codons):
 high = high_avg.get(codon, 0)
 low = low_avg.get(codon, 0)
 delta = high - low
 total = total_avg.get(codon, 0)

 if high > 1.00 and delta >= 0.08:
 is_optimal = 'Yes'
 optimal_codons.append(codon)
 else:
 is_optimal = 'No'

 all_results.append({
 'Species': species,
 'Codon': codon,
 'High_avg_RSCU': round(high, 4),
 'Low_avg_RSCU': round(low, 4),
 'Delta_RSCU': round(delta, 4),
 'Total_avg_RSCU': round(total, 4),
 'Is_Optimal': is_optimal
 })

 gene_lists.append({
 'Species': species,
 'High_expression_genes (top 10%)': ', '.join(high_genes),
 'Low_expression_genes (bottom 10%)': ', '.join(low_genes),
 'N_high': n_high,
 'N_low': n_low
 })

 print(f" [OK] {len(optimal_codons)} ")
 print(f" {', '.join(sorted(optimal_codons)) if optimal_codons else 'None'}")

df_detail = pd.DataFrame(all_results)
df_detail = df_detail[['Species', 'Codon', 'High_avg_RSCU', 'Low_avg_RSCU',
 'Delta_RSCU', 'Total_avg_RSCU', 'Is_Optimal']]

df_lists = pd.DataFrame(gene_lists)

with pd.ExcelWriter(output_file) as writer:
 df_detail.to_excel(writer, sheet_name='Detailed_RSCU', index=False)
 df_lists.to_excel(writer, sheet_name='Gene_Lists', index=False)
```

```
print(f"\n[OK] : {output_file}")

species_list = df_detail['Species'].unique()
optimal_by_species = {}
for sp in species_list:
 codons = df_detail[(df_detail['Species'] == sp) & (df_detail['Is_Optimal'] ==
'Yes')]['Codon'].tolist()
 optimal_by_species[sp] = set(codons)

if species_list:
 common_optimal = set.intersection(*[optimal_by_species[sp] for sp in species_list])
 print(f"\n (): {', '.join(sorted(common_optimal)) if common_optimal else 'None'}")
```

Step 09. Pooled Correlation

Pearson correlation among ENC/GC1/GC2/GC3 across 242 genes.

Input: codon_parameter.xlsx

Output: correlation_heatmap.pdf + results.xlsx (Figure 2D)

Method: Pearson correlation; pooled

Source Code (step09_correlation.py):

```
import pandas as pd
import numpy as np
import seaborn as sns
import matplotlib.pyplot as plt
import matplotlib as mpl
from scipy.stats import pearsonr
import os
# ---------------------------------------------------------------------------
# Repository configuration (ADDED for public release - see CHANGES.md)
# Only the hard-coded local paths were parameterised; no algorithm, threshold,
# formula, sort order or output content was modified.
# CUB_DATA_DIR : directory holding the input data (default:../data)
# CUB_OUT_DIR : directory for outputs (default:../output)
# ---------------------------------------------------------------------------
import os as _os
_HERE = _os.path.dirname(_os.path.abspath(__file__))
DATA_DIR = _os.environ.get("CUB_DATA_DIR", _os.path.normpath(_os.path.join(_HERE,
_os.pardir, "data")))
OUT_DIR = _os.environ.get("CUB_OUT_DIR", _os.path.normpath(_os.path.join(_HERE,
_os.pardir, "output")))
_os.makedirs(OUT_DIR, exist_ok=True)
# ---------------------------------------------------------------------------

mpl.rcParams['font.family'] = 'Times New Roman'
mpl.rcParams['mathtext.fontset'] = 'stix'

desktop = DATA_DIR
input_file = os.path.join(desktop, "codon_parameter.xlsx")
output_pdf = os.path.join(desktop, "correlation_heatmap_no_GC12.pdf")
output_png = os.path.join(desktop, "correlation_heatmap_no_GC12.png")

# ===== Read data =====
df = pd.read_excel(input_file)

expected_cols = ['Species', 'Gene', 'ENC', 'GC1', 'GC2', 'GC3']
df.columns = df.columns.str.strip()
```

```
col_map = {}
for col in df.columns:
 if col.lower() in ['species', 'gene', 'enc', 'gc1', 'gc2', 'gc3']:
 col_map[col.lower()] = col
species_col = col_map.get('species', 'Species')
gene_col = col_map.get('gene', 'Gene')
enc_col = col_map.get('enc', 'ENC')
gc1_col = col_map.get('gc1', 'GC1')
gc2_col = col_map.get('gc2', 'GC2')
gc3_col = col_map.get('gc3', 'GC3')

data = df[[enc_col, gc1_col, gc2_col, gc3_col]].copy()
data.columns = ['ENC', 'GC1', 'GC2', 'GC3']
data = data.apply(pd.to_numeric, errors='coerce').dropna()

params = ['ENC', 'GC1', 'GC2', 'GC3']
corr_matrix = np.zeros((4, 4))
p_matrix = np.ones((4, 4))

for i, p1 in enumerate(params):
 for j, p2 in enumerate(params):
 r, p = pearsonr(data[p1], data[p2])
 corr_matrix[i, j] = r
 p_matrix[i, j] = p

corr_df = pd.DataFrame(corr_matrix, index=params, columns=params)
p_df = pd.DataFrame(p_matrix, index=params, columns=params)

fig, ax = plt.subplots(figsize=(6, 5))

mask = np.zeros_like(corr_df, dtype=bool)
mask[np.tril_indices_from(mask, k=-1)] = True

sns.heatmap(corr_df, mask=mask, annot=True, fmt='.3f', cmap='coolwarm',
 center=0, square=True, linewidths=0.5, cbar_kws={"shrink": 0.8, "label": "Pearson
correlation"},
 annot_kws={"size": 10}, ax=ax, vmin=-1, vmax=1)

for i in range(4):
 for j in range(i, 4):
 if i == j:
 continue
 p_val = p_df.iloc[i, j]
 if p_val < 0.001:
 star = '***'
 elif p_val < 0.01:
 star = '**'
 elif p_val < 0.05:
 star = '*'
 else:
 star = ''
 if star:
 x = j + 0.5
 y = i + 0.5
 ax.text(x, y+0.1, star, ha='center', va='center', fontsize=10, color='black',
fontweight='bold')

ax.set_xlabel('Parameters', fontsize=12)
ax.set_ylabel('Parameters', fontsize=12)
plt.title('Correlation heatmap (ENC, GC1, GC2, GC3)', fontsize=14)
```

```
plt.tight_layout()

plt.savefig(output_pdf, dpi=300, bbox_inches='tight')
plt.savefig(output_png, dpi=300, bbox_inches='tight')
print(f"[OK] : {output_pdf}")
print(f"[OK] : {output_png}")

print("\n[STATS] Pearson:")
print(corr_df.round(4))
print("\n[STATS] P:")
print(p_df.round(4))

output_excel = os.path.join(desktop, "correlation_results_no_GC12.xlsx")
with pd.ExcelWriter(output_excel) as writer:
 corr_df.to_excel(writer, sheet_name='Correlation')
 p_df.to_excel(writer, sheet_name='P_values')
print(f"[OK] : {output_excel}")

# plt.show() # disabled: blocks unattended execution
```

Step 10a. Four-Gene Concatenation

Extract and concatenate atpB, matK, ndhF, rbcL.

Input: species_CDS_filtered.fasta

Output: concatenated_4genes.fasta (MEGA11 input)

Method: Targeted extraction; concatenation

Source Code (step10a_concatenate_4genes.py):

```
from Bio import SeqIO
import os
# ---------------------------------------------------------------------------
# Repository configuration (ADDED for public release - see CHANGES.md)
# Only the hard-coded local paths were parameterised; no algorithm, threshold,
# formula, sort order or output content was modified.
# CUB_DATA_DIR : directory holding the input data (default:../data)
# CUB_OUT_DIR : directory for outputs (default:../output)
# ---------------------------------------------------------------------------
import os as _os
_HERE = _os.path.dirname(_os.path.abspath(__file__))
DATA_DIR = _os.environ.get("CUB_DATA_DIR", _os.path.normpath(_os.path.join(_HERE,
_os.pardir, "data")))
OUT_DIR = _os.environ.get("CUB_OUT_DIR", _os.path.normpath(_os.path.join(_HERE,
_os.pardir, "output")))
_os.makedirs(OUT_DIR, exist_ok=True)
# ---------------------------------------------------------------------------

desktop = DATA_DIR

target_genes = ["rbcL", "matK", "ndhF", "atpB"]

species_files = {
 "": "watermelon_CDS_filtered.fasta",
 "": "melon_CDS_filtered.fasta",
 "": "cucumber_CDS_filtered.fasta",
 "": "pumpkin_CDS_filtered.fasta",
 "": "winter_melon_CDS_filtered.fasta",
 "": "bitter_melon_CDS_filtered.fasta",
```

```
}

species_labels = {
 "": "C. lanatus",
 "": "C. melo",
 "": "C. sativus",
 "": "C. moschata",
 "": "B. hispida",
 "": "M. charantia",
}

all_seqs = {}
for species, filename in species_files.items():
 filepath = os.path.join(desktop, filename)
 if not os.path.exists(filepath):
 print(f"[WARN] File not found: {filename}")
 continue
 genes = {}
 for record in SeqIO.parse(filepath, 'fasta'):
 gene_name = record.id
 seq = str(record.seq)
 genes[gene_name] = seq
 all_seqs[species] = genes

print("[STATS] :")
for species in species_files.keys():
 present = []
 missing = []
 for gene in target_genes:
 if gene in all_seqs.get(species, {}):
 present.append(gene)
 else:
 missing.append(gene)
 print(f" {species_labels[species]}: {len(present)}/{len(target_genes)}")
 if missing:
 print(f" : {', '.join(missing)}")

output_fasta = os.path.join(desktop, "concatenated_4genes.fasta")
with open(output_fasta, 'w', encoding='utf-8') as out:
 for species in species_files.keys():
 seq_parts = []
 for gene in target_genes:
 seq = all_seqs.get(species, {}).get(gene, None)
 if seq:
 seq_parts.append(seq)
 else:
 seq_parts.append('N' * 300)
 concatenated = ''.join(seq_parts)
 label = species_labels.get(species, species)
 out.write(f">{label}\n{concatenated}\n")

print(f"\n[OK] : {output_fasta}")
print(f" : {len(concatenated)} bp")
print(f" Species: {len(species_files)}")
```

Step 10b. All-CDS Concatenation

Concatenate all shared CDS genes for plastome-wide tree.

Input: species_CDS_filtered.fasta

Output: concatenated_cds_for_phylogeny.fasta

Method: Gene set intersection; concatenation

Source Code (step10b_concatenate_all_cds.py):

```
from Bio import SeqIO
import os
# ----------------------------------------------------------------------------
# Repository configuration (ADDED for public release - see CHANGES.md)
# Only the hard-coded local paths were parameterised; no algorithm, threshold,
# formula, sort order or output content was modified.
# CUB_DATA_DIR : directory holding the input data (default:../data)
# CUB_OUT_DIR : directory for outputs (default:../output)
# ----------------------------------------------------------------------------
import os as _os
_HERE = _os.path.dirname(_os.path.abspath(__file__))
DATA_DIR = _os.environ.get("CUB_DATA_DIR", _os.path.normpath(_os.path.join(_HERE,
_os.pardir, "data")))
OUT_DIR = _os.environ.get("CUB_OUT_DIR", _os.path.normpath(_os.path.join(_HERE,
_os.pardir, "output")))
_os.makedirs(OUT_DIR, exist_ok=True)
# ----------------------------------------------------------------------------

desktop = DATA_DIR

species_files = {
 "": "watermelon_CDS_filtered.fasta",
 "": "melon_CDS_filtered.fasta",
 "": "cucumber_CDS_filtered.fasta",
 "": "pumpkin_CDS_filtered.fasta",
 "": "winter_melon_CDS_filtered.fasta",
 "": "bitter_melon_CDS_filtered.fasta",
}

species_labels = {
 "": "C. lanatus",
 "": "C. melo",
 "": "C. sativus",
 "": "C. moschata",
 "": "B. hispida",
 "": "M. charantia",
}

species_genes = {}
for species, filename in species_files.items():
 filepath = os.path.join(desktop, filename)
 if not os.path.exists(filepath):
 print(f"[WARN] File not found: {filename}")
 continue
 genes = {}
 for record in SeqIO.parse(filepath, 'fasta'):
 gene_name = record.id
 seq = str(record.seq)
 genes[gene_name] = seq
 species_genes[species] = genes
 print(f"{species_labels[species]}: {len(genes)} ")
```

```
gene_sets = [set(genes.keys()) for genes in species_genes.values()]
common_genes = set.intersection(*gene_sets)
print(f"\n: {len(common_genes)}")

common_genes_sorted = sorted(common_genes)

concatenated = {}
seq_length = 0
for species, genes in species_genes.items():
 seq_list = []
 for gene in common_genes_sorted:
 if gene in genes:
 seq_list.append(genes[gene])
 else:
 seq_list.append('N' * 300)
 concatenated[species] = ''.join(seq_list)
 if seq_length == 0:
 seq_length = len(concatenated[species])

print(f"\n: {seq_length} bp")

output_fasta = os.path.join(desktop, "concatenated_cds_for_phylogeny.fasta")
with open(output_fasta, 'w', encoding='utf-8') as out:
 for species, seq in concatenated.items():
 label = species_labels.get(species, species)
 out.write(f">{label}\n{seq}\n")

print(f"\n[OK] : {output_fasta}")
print(f" : {seq_length} bp")
print(f" Species: {len(concatenated)}")

print("\n[STATS] :")
for species, genes in species_genes.items():
 common_count = len([g for g in common_genes_sorted if g in genes])
 print(f" {species_labels[species]}: {common_count} / {len(common_genes_sorted)}")

print("\n[DONE] Done! MEGA.")
```

Step 11a. Start-Codon Filter Comparison

Compare ATG-only vs ATG/GTG/TTG filtering (Table S7).

Input: species_CDS.fasta (unfiltered)

Output: ATG_vs_GTG_TTG_comparison.csv

Method: Two-strategy comparison

Source Code (step11a_startcodon_compare.py):

```
from Bio import SeqIO
import os
import pandas as pd
# ---------------------------------------------------------------------------
# Repository configuration (ADDED for public release - see CHANGES.md)
# Only the hard-coded local paths were parameterised; no algorithm, threshold,
# formula, sort order or output content was modified.
# CUB_DATA_DIR : directory holding the input data (default:../data)
# CUB_OUT_DIR : directory for outputs (default:../output)
# ---------------------------------------------------------------------------
import os as _os
```

```
_HERE = _os.path.dirname(_os.path.abspath(__file__))
DATA_DIR = _os.environ.get("CUB_DATA_DIR", _os.path.normpath(_os.path.join(_HERE,
_os.pardir, "data")))
OUT_DIR = _os.environ.get("CUB_OUT_DIR", _os.path.normpath(_os.path.join(_HERE,
_os.pardir, "output")))
_os.makedirs(OUT_DIR, exist_ok=True)
# ---------------------------------------------------------------------------

desktop = DATA_DIR

species_files = {
 "": "watermelon_CDS.fasta",
 "": "melon_CDS.fasta",
 "": "cucumber_CDS.fasta",
 "": "pumpkin_CDS.fasta",
 "": "winter melon_CDS.fasta",
 "": "bitter melon_CDS.fasta",
}

def filter_cds(records, start_condition, min_len=300):
 """: + + """
 seen = set()
 kept = {}
 for rec in records:
 seq = str(rec.seq).upper()
 gene = rec.id

 if start_condition(seq):
 if len(seq) >= min_len:
 if gene not in seen:
 seen.add(gene)
 kept[gene] = len(seq)
 return kept

results = []
non_atg_genes_by_species = {}

for species, fname in species_files.items():
 fpath = os.path.join(desktop, fname)
 if not os.path.exists(fpath):
 print(f"[WARN] File not found: {fpath}")
 continue

 records = list(SeqIO.parse(fpath, 'fasta'))

 def atg_only(seq): return seq.startswith('ATG')
 kept_a = filter_cds(records, atg_only)

 def atg_gtg_ttg(seq): return any(seq.startswith(start) for start in ['ATG', 'GTG',
'TTG'])
 kept_b = filter_cds(records, atg_gtg_ttg)

 results.append({
 '': species,
 'CDS': len(records),
 'A': len(kept_a),
 'B': len(kept_b),
 '': len(kept_b) - len(kept_a),
 })
```

```
 lost_a = set(kept_b.keys()) - set(kept_a.keys())
 if lost_a:
 non_atg_genes_by_species[species] = sorted(lost_a)

df = pd.DataFrame(results)
print("\n[STATS] ")
print(df.to_string(index=False))

print("\n AB(ATG,>=300bp,):")
for species, genes in non_atg_genes_by_species.items():
 print(f"\n{species}({len(genes)}):")
 for g in genes:
 print(f" - {g}")

output_csv = os.path.join(desktop, "ATG_vs_GTG_TTG_.csv")
df.to_csv(output_csv, index=False)
print(f"\n[OK] : {output_csv}")
```

Step 11b. Differing Gene Lists

List genes differing between start-codon strategies.

Input: species_CDS.fasta

Output: diff_genes_ATG_vs_GTGTTG.txt

Method: Set difference

Source Code (step11b_diff_genes.py):

```
from Bio import SeqIO
import os
# ---------------------------------------------------------------------------
# Repository configuration (ADDED for public release - see CHANGES.md)
# Only the hard-coded local paths were parameterised; no algorithm, threshold,
# formula, sort order or output content was modified.
# CUB_DATA_DIR : directory holding the input data (default:../data)
# CUB_OUT_DIR : directory for outputs (default:../output)
# ---------------------------------------------------------------------------
import os as _os
_HERE = _os.path.dirname(_os.path.abspath(__file__))
DATA_DIR = _os.environ.get("CUB_DATA_DIR", _os.path.normpath(_os.path.join(_HERE,
_os.pardir, "data")))
OUT_DIR = _os.environ.get("CUB_OUT_DIR", _os.path.normpath(_os.path.join(_HERE,
_os.pardir, "output")))
_os.makedirs(OUT_DIR, exist_ok=True)
# ---------------------------------------------------------------------------

desktop = DATA_DIR

species_files = {
 "": "watermelon_CDS.fasta",
 "": "melon_CDS.fasta",
 "": "cucumber_CDS.fasta",
 "": "pumpkin_CDS.fasta",
 "": "winter melon_CDS.fasta",
 "": "bitter melon_CDS.fasta",

}

def filter_cds(records, start_condition, min_len=300):
```

```
 kept = {}
 seen = set()
 for rec in records:
 seq = str(rec.seq).upper()
 gene = rec.id
 if start_condition(seq) and len(seq) >= min_len and gene not in seen:
 seen.add(gene)
 kept[gene] = seq
 return kept

output_file = os.path.join(desktop, "diff_genes_ATG_vs_GTGTTG.txt")

with open(output_file, 'w') as out:
 out.write(":B(ATG/GTG/TTG) vs A(ATG)\n")
 out.write("=" * 60 + "\n\n")

 for species, fname in species_files.items():
 fpath = os.path.join(desktop, fname)
 if not os.path.exists(fpath):
 out.write(f"[WARN] File not found: {fpath}\n")
 continue

 records = list(SeqIO.parse(fpath, 'fasta'))
 out.write(f"[{species}]\n")
 out.write("-" * 40 + "\n")

 def atg_only(s): return s.startswith('ATG')
 kept_a = filter_cds(records, atg_only)

 def atg_gtg_ttg(s): return any(s.startswith(start) for start in ['ATG', 'GTG', 'TTG'])
 kept_b = filter_cds(records, atg_gtg_ttg)

 diff_genes = set(kept_b.keys()) - set(kept_a.keys())

 if not diff_genes:
 out.write("None.\n\n")
 else:
 out.write(f" {len(diff_genes)} :\n")
 for gene in sorted(diff_genes):
 seq = kept_b[gene]
 start_codon = seq[:3]
 length = len(seq)
 out.write(f" {gene} : {start_codon}, {length} bp\n")
 out.write("\n")

 out.write("\n")

print(f"[OK] Done: {output_file}")
```

Step 11c. Length Proportion Analysis

Length proportion of differing genes.

Input: species_CDS.fasta

Output: diff_gene_length_ratio.csv

Method: Length distribution analysis

Source Code (step11c_length_proportion.py):

```
from Bio import SeqIO
import os
# ---------------------------------------------------------------------------
# Repository configuration (ADDED for public release - see CHANGES.md)
# Only the hard-coded local paths were parameterised; no algorithm, threshold,
# formula, sort order or output content was modified.
# CUB_DATA_DIR : directory holding the input data (default:../data)
# CUB_OUT_DIR : directory for outputs (default:../output)
# ---------------------------------------------------------------------------
import os as _os
_HERE = _os.path.dirname(_os.path.abspath(__file__))
DATA_DIR = _os.environ.get("CUB_DATA_DIR", _os.path.normpath(_os.path.join(_HERE,
_os.pardir, "data")))
OUT_DIR = _os.environ.get("CUB_OUT_DIR", _os.path.normpath(_os.path.join(_HERE,
_os.pardir, "output")))
_os.makedirs(OUT_DIR, exist_ok=True)
# ---------------------------------------------------------------------------

desktop = DATA_DIR

species_files = {
 "": "watermelon_CDS.fasta",
 "": "melon_CDS.fasta",
 "": "cucumber_CDS.fasta",
 "": "pumpkin_CDS.fasta",
 "": "winter melon_CDS.fasta",
 "": "bitter melon_CDS.fasta",
}

def filter_cds(records, start_condition, min_len=300):
 kept = {}
 seen = set()
 for rec in records:
 seq = str(rec.seq).upper()
 gene = rec.id
 if start_condition(seq) and len(seq) >= min_len and gene not in seen:
 seen.add(gene)
 kept[gene] = seq
 return kept

results = []

for species, fname in species_files.items():
 fpath = os.path.join(desktop, fname)
 if not os.path.exists(fpath):
 print(f"[WARN] File not found: {fpath}")
 continue

 records = list(SeqIO.parse(fpath, 'fasta'))

 def atg_only(s): return s.startswith('ATG')
 kept_a = filter_cds(records, atg_only)

 def atg_gtg_ttg(s): return any(s.startswith(start) for start in ['ATG', 'GTG', 'TTG'])
 kept_b = filter_cds(records, atg_gtg_ttg)

 total_len_b = sum(len(s) for s in kept_b.values())
 diff_genes = set(kept_b.keys()) - set(kept_a.keys())
 diff_len = sum(len(kept_b[g]) for g in diff_genes)
```

```
 ratio = (diff_len / total_len_b * 100) if total_len_b > 0 else 0

 results.append({
 "": species,
 "B": len(kept_b),
 "": len(diff_genes),
 "B(bp)": total_len_b,
 "(bp)": diff_len,
 "(%)": round(ratio, 4)
 })

 if diff_genes:
 print(f"\n[{species}]:")
 for g in sorted(diff_genes):
 seq = kept_b[g]
 print(f" {g}: {seq[:3]}, {len(seq)}bp")

print("\n[STATS] ")
print("-" * 70)
print(f"{'':<8} {'B':<10} {'':<10} {'B(bp)':<12} {'(bp)':<12} {'(%)':<8}")
for r in results:
 print(f"{r['']:<8} {r['B']:<10} {r['']:<10} {r['B(bp)']:<12} {r['(bp)']:<12}
{r['(%)']:<8}")

import pandas as pd
df = pd.DataFrame(results)
output_csv = os.path.join(desktop, "diff_gene_length_ratio.csv")
df.to_csv(output_csv, index=False, encoding='utf-8-sig')
print(f"\n[OK] : {output_csv}")
```

Step 12. Per-Species Correlation (R2 #9)

Per-species, gene-level, pooled correlation with BH-FDR; power analysis.

Input: codon_parameter.xlsx

Output: Fig_R1 heatmaps.pdf, Fig_R2 forest plot.pdf, results_v2.xlsx (Table S10)

Method: Pearson+Spearman; BH-FDR; power analysis

Source Code (step12_per_species_correlation.py):

```
# -*- coding: utf-8 -*-
"""
Per-species and gene-level correlation analysis (Reviewer 2, Comment 9)
Per-species and gene-level correlation analysis (Reviewer 2, Comment 9)

: Pearson ,
,.:(a) ;(b) and
gene identity;(c).

Design:
 1 Pooled : N=242, gene×species (,)
 2 Per-species : N=39~41, -> (a)
 3 Gene-level : N=41,,
 -> "gene identity"(gene identity) -> (b)
 : BH-FDR( 6 )+ BH-FDR(42 ) -> (c)
 :Pearson and Spearman
 :(N≈40 )

: 9_SPSS//codon_parameter.xlsx(242 ),
```

```
 Do not re-extract or recalculate from.gb, to ensure exact match with manuscript tables.
: PDF(TrueType , pdf.fonttype=42)+ PNG + Excel
"""

import os
import numpy as np
import pandas as pd
import matplotlib
matplotlib.use('Agg')
import matplotlib.pyplot as plt
import seaborn as sns
from scipy.stats import pearsonr, spearmanr, norm, false_discovery_control
from itertools import combinations
# ---------------------------------------------------------------------------
# Repository configuration (ADDED for public release - see CHANGES.md)
# Only the hard-coded local paths were parameterised; no algorithm, threshold,
# formula, sort order or output content was modified.
# CUB_DATA_DIR : directory holding the input data (default:../data)
# CUB_OUT_DIR : directory for outputs (default:../output)
# ---------------------------------------------------------------------------
import os as _os
_HERE = _os.path.dirname(_os.path.abspath(__file__))
DATA_DIR = _os.environ.get("CUB_DATA_DIR", _os.path.normpath(_os.path.join(_HERE,
_os.pardir, "data")))
OUT_DIR = _os.environ.get("CUB_OUT_DIR", _os.path.normpath(_os.path.join(_HERE,
_os.pardir, "output")))
_os.makedirs(OUT_DIR, exist_ok=True)
# ---------------------------------------------------------------------------

matplotlib.rcParams['pdf.fonttype'] = 42
matplotlib.rcParams['ps.fonttype'] = 42
matplotlib.rcParams['font.family'] = 'Times New Roman'
matplotlib.rcParams['mathtext.fontset'] = 'stix'

WS = os.path.normpath(os.path.join(os.path.dirname(os.path.abspath(__file__)), os.pardir))
INPUT_XLSX = os.path.join(DATA_DIR, "codon_parameter.xlsx")
OUT_DIR = os.environ.get("CUB_OUT_DIR", os.path.join(WS, "output"))
os.makedirs(OUT_DIR, exist_ok=True)

PARAMS = ['ENC', 'GC1', 'GC2', 'GC3']
PAIRS = list(combinations(PARAMS, 2))
SPECIES_ORDER = ['C. lanatus', 'C. melo', 'C. sativus',
 'C. moschata', 'B. hispida', 'M. charantia']

SEP = '=' * 74


def bh(pvals):
 """Benjamini-Hochberg FDR correction (scipy implementation)."""
 p = np.asarray(pvals, dtype=float)
 return np.asarray(false_discovery_control(p, method='bh'), dtype=float)


def r_ci95(r, n):
 """Pearson r 95% (Fisher z )."""
 if n <= 3:
 return (np.nan, np.nan)
 z = np.arctanh(np.clip(r, -0.999999, 0.999999))
 se = 1.0 / np.sqrt(n - 3)
 lo, hi = np.tanh(z - 1.96 * se), np.tanh(z + 1.96 * se)
```

```
 return (lo, hi)


def required_n(r, alpha=0.05, power=0.80):
 """ |r| (Fisher z )."""
 r = abs(r)
 if r < 1e-6:
 return np.inf
 z_a = norm.ppf(1 - alpha / 2)
 z_b = norm.ppf(power)
 return ((z_a + z_b) / np.arctanh(r)) ** 2 + 3


def stars(p):
 if p < 0.001:
 return '***'
 if p < 0.01:
 return '**'
 if p < 0.05:
 return '*'
 return ''


def corr_block(df):
 """ 4×4 Pearson / Spearman r and p."""
 n = len(df)
 res = {}
 for kind, func in (('pearson', pearsonr), ('spearman', spearmanr)):
 R = np.full((4, 4), np.nan)
 P = np.full((4, 4), np.nan)
 for i, j in combinations(range(4), 2):
 r, p = func(df[PARAMS[i]], df[PARAMS[j]])
 R[i, j] = R[j, i] = r
 P[i, j] = P[j, i] = p
 np.fill_diagonal(R, 1.0)
 np.fill_diagonal(P, 0.0)
 res[kind] = {'r': R, 'p': P}
 return n, res


# ======================================================================
# ======================================================================
print(SEP)
print('0. Data source verification')
print(SEP)
df = pd.read_excel(INPUT_XLSX)
df.columns = df.columns.str.strip()
print(f' Input file : {INPUT_XLSX}')
print(f' Shape : {df.shape} (,/)')
print(f' Columns : {list(df.columns)}')
print(f' Species : {df.Species.nunique()} Genes: {df.Gene.nunique()}')
counts = df.Species.value_counts()
for sp in SPECIES_ORDER:
 print(f' {sp:<16} n = {counts.get(sp, 0)}')
assert df[PARAMS].notna().all().all(), 'Missing values'
print(f' Missing values : None')

# ======================================================================
# ======================================================================
print()
```

```
print(SEP)
print('1. (Pearson and Spearman)')
print(SEP)

blocks = {} # key -> (label, df)
blocks[('Pooled', 'All six species (pooled)')] = ('Pooled', df)

for sp in SPECIES_ORDER:
 sub = df[df.Species == sp].reset_index(drop=True)
 blocks[('Per-species', sp)] = (sp, sub)

gene_nsp = df.groupby('Gene').Species.nunique()
gene_level = (df.groupby('Gene')[PARAMS].mean()
.assign(n_species=gene_nsp)
.reset_index())
blocks[('Gene-level', 'Gene means across species')] = ('Gene-level', gene_level)

records = []
for (level, label), (disp, d) in blocks.items():
 n, res = corr_block(d)
 for kind in ('pearson', 'spearman'):
 for (p1, p2) in PAIRS:
 i, j = PARAMS.index(p1), PARAMS.index(p2)
 r = res[kind]['r'][i, j]
 p = res[kind]['p'][i, j]
 lo, hi = r_ci95(r, n) if kind == 'pearson' else (np.nan, np.nan)
 records.append({
 'Level': level, 'Subset': disp, 'Method': kind.capitalize(),
 'Pair': f'{p1}-{p2}', 'N': n,
 'r': r, 'P_raw': p,
 'r_CI95_low': lo, 'r_CI95_high': hi,
 })
 print(f' [{level:<12}] {disp:<32} n = {n}')

res_df = pd.DataFrame(records)

# ======================================================================
# ======================================================================
print()
print(SEP)
print('2. (Benjamini-Hochberg FDR)')
print(SEP)

res_df['P_FDR_within_level'] = np.nan
for key, idx in res_df.groupby(['Level', 'Subset', 'Method']).groups.items():
 idx = list(idx)
 res_df.loc[idx, 'P_FDR_within_level'] = bh(res_df.loc[idx, 'P_raw'].values)

res_df['P_FDR_global'] = np.nan
for method, idx in res_df.groupby('Method').groups.items():
 idx = list(idx)
 res_df.loc[idx, 'P_FDR_global'] = bh(res_df.loc[idx, 'P_raw'].values)

res_df['Sig_raw'] = res_df.P_raw < 0.05
res_df['Sig_FDR_level'] = res_df.P_FDR_within_level < 0.05
res_df['Sig_FDR_global'] = res_df.P_FDR_global < 0.05

for method in ('Pearson', 'Spearman'):
 sub = res_df[res_df.Method == method]
 print(f' {method}: {len(sub)},'
```

```
 f' P<0.05 {int(sub.Sig_raw.sum())} ,'
 f' FDR<0.05 {int(sub.Sig_FDR_level.sum())} ,'
 f' FDR<0.05 {int(sub.Sig_FDR_global.sum())} ')

# ======================================================================
# ======================================================================
print()
print(SEP)
print('3. (Pearson)')
print(SEP)

pear = res_df[res_df.Method == 'Pearson']
summary = []
for pair in [f'{a}-{b}' for a, b in PAIRS]:
 pooled = pear[(pear.Level == 'Pooled') & (pear.Pair == pair)].iloc[0]
 persp = pear[(pear.Level == 'Per-species') & (pear.Pair == pair)]
 gl = pear[(pear.Level == 'Gene-level') & (pear.Pair == pair)].iloc[0]
 rv = persp.r.values
 same_dir = int(np.sum(np.sign(rv) == np.sign(pooled.r)))
 summary.append({
 'Pair': pair,
 'Pooled_r': round(pooled.r, 4),
 'Pooled_P_raw': pooled.P_raw,
 'Pooled_P_FDR': pooled.P_FDR_within_level,
 'Pooled_N': int(pooled.N),
 'PerSpecies_r_min': round(float(rv.min()), 4),
 'PerSpecies_r_max': round(float(rv.max()), 4),
 'PerSpecies_r_mean': round(float(rv.mean()), 4),
 'PerSpecies_r_SD': round(float(rv.std(ddof=1)), 4),
 'N_species_same_direction': f'{same_dir}/6',
 'N_species_sig_FDR': int(persp.Sig_FDR_level.sum()),
 'GeneLevel_r': round(gl.r, 4),
 'GeneLevel_P_FDR': gl.P_FDR_within_level,
 'GeneLevel_N': int(gl.N),
 'Effect_size_consistent': 'Yes' if abs(pooled.r - rv.mean()) < 0.10 else 'No',
 })
sum_df = pd.DataFrame(summary)

for row in sum_df.itertuples():
 print(f' {row.Pair:<9} pooled r={row.Pooled_r:+.3f} (N={row.Pooled_N}) | '
 f'per-species r={row.PerSpecies_r_min:+.3f}~{row.PerSpecies_r_max:+.3f}, '
 f'mean={row.PerSpecies_r_mean:+.3f} (SD={row.PerSpecies_r_SD:.3f}) | '
 f' {row.N_species_same_direction} | gene-level r={row.GeneLevel_r:+.3f} | '
 f': {row.Effect_size_consistent}')

# ======================================================================
# ======================================================================
print()
print(SEP)
print('4. :N≈40 ?')
print(SEP)

min_n = int(counts.min())
power_rows = []
for target_r in (0.20, 0.30, 0.35, 0.40, 0.50):
 nreq = required_n(target_r)
 power_rows.append({
 'Target_abs_r': target_r,
 'N_required_80pct_power': int(np.ceil(nreq)),
```

```
 'Detectable_at_N40': 'Yes' if nreq <= min_n else 'No',
 })
 print(f' |r| = {target_r:.2f} N ≈ {int(np.ceil(nreq)):>4} '
 f'(80% , α=0.05) -> N={min_n} : '
 f'{"" if nreq <= min_n else ""}')

z_a, z_b = norm.ppf(0.975), norm.ppf(0.80)
r_min_detect = np.tanh((z_a + z_b) / np.sqrt(min_n - 3))
print(f' -> N={min_n},80% , |r| ≈ {r_min_detect:.3f}')
power_df = pd.DataFrame(power_rows)
power_df.loc[len(power_df)] = {
 'Target_abs_r': f'min detectable at N={min_n}',
 'N_required_80pct_power': min_n,
 'Detectable_at_N40': round(r_min_detect, 4),
}

# ======================================================================
# ======================================================================
print()
print(SEP)
print('5. Plot')
print(SEP)

panel_order = ([('Per-species', sp) for sp in SPECIES_ORDER]
 + [('Pooled', 'All six species (pooled)'),
 ('Gene-level', 'Gene means across species')])
panel_titles = {
 'C. lanatus': 'C. lanatus', 'C. melo': 'C. melo', 'C. sativus': 'C. sativus',
 'C. moschata': 'C. moschata', 'B. hispida': 'B. hispida',
 'M. charantia': 'M. charantia',
 'All six species (pooled)': 'Pooled (all six species)',
 'Gene means across species': 'Gene-level (gene means)',
}

fig, axes = plt.subplots(2, 4, figsize=(17, 8.6))
mask = np.tril(np.ones((4, 4), dtype=bool), k=-1)

for ax, key in zip(axes.flat, panel_order):
 level, label = key
 disp, d = blocks[key]
 n, res = corr_block(d)
 R = pd.DataFrame(res['pearson']['r'], index=PARAMS, columns=PARAMS)
 P = pd.DataFrame(res['pearson']['p'], index=PARAMS, columns=PARAMS)

 ann = np.empty((4, 4), dtype=object)
 for i in range(4):
 for j in range(4):
 if mask[i, j]:
 ann[i, j] = ''
 elif i == j:
 ann[i, j] = '1.00'
 else:
 pf = res_df[(res_df.Level == level) & (res_df.Subset == disp)
 & (res_df.Method == 'Pearson')
 & (res_df.Pair == f'{PARAMS[i]}-{PARAMS[j]}')].iloc[0]
 mark = stars(pf.P_FDR_within_level)
 ann[i, j] = f'{R.iloc[i, j]:.2f}{mark}'
 ann_df = pd.DataFrame(ann, index=PARAMS, columns=PARAMS)

 sns.heatmap(R, mask=mask, annot=ann_df, fmt='', cmap='RdBu_r', center=0,
```

```
 square=True, linewidths=0.6, ax=ax, vmin=-1, vmax=1,
 cbar_kws={'shrink': 0.72, 'label': 'Pearson r'},
 annot_kws={'size': 10})
 lvl_txt = ('Pooled' if level == 'Pooled'
 else 'Gene-level' if level == 'Gene-level' else 'Per-species')
 ax.set_title(f'{panel_titles[label]} (n={n})', fontsize=12, fontweight='bold', pad=8)
 ax.set_xlabel(f'{lvl_txt} analysis', fontsize=10)
 ax.set_ylabel('')
 ax.tick_params(labelsize=10)

fig.suptitle('Correlation among codon usage parameters (ENC, GC1, GC2, GC3): '
 'per-species, pooled, and gene-level analyses',
 fontsize=14, fontweight='bold', y=0.995)
fig.text(0.5, 0.012,
 'Values are Pearson r; asterisks denote significance after '
 'Benjamini-Hochberg FDR correction within each analysis '
 '(* P<0.05, ** P<0.01, *** P<0.001).',
 ha='center', fontsize=10, style='italic')
fig.tight_layout(rect=[0, 0.035, 1, 0.965])

fig1_pdf = os.path.join(OUT_DIR, 'Fig_R1_per_species_correlation_heatmaps.pdf')
fig1_png = os.path.join(OUT_DIR, 'Fig_R1_per_species_correlation_heatmaps.png')
fig.savefig(fig1_pdf, bbox_inches='tight')
fig.savefig(fig1_png, dpi=300, bbox_inches='tight')
plt.close(fig)
print(f' 1 (8 ,None): {os.path.basename(fig1_pdf)}')

# ======================================================================
# ======================================================================
pair_labels = [f'{a}-{b}' for a, b in PAIRS]
pair_keys = [f'{a}-{b}' for a, b in PAIRS]
fig2, ax = plt.subplots(figsize=(11.5, 7.2))
ypos = np.arange(len(pair_keys))[::-1]
row_h = 0.115

sp_colors = plt.cm.tab10(np.linspace(0, 1, 6))

for k, pair in enumerate(pair_keys):
 y0 = ypos[k]
 persp = pear[(pear.Level == 'Per-species') & (pear.Pair == pair)]
 pooled = pear[(pear.Level == 'Pooled') & (pear.Pair == pair)].iloc[0]
 gl = pear[(pear.Level == 'Gene-level') & (pear.Pair == pair)].iloc[0]

 for m, (sp, row) in enumerate(persp.iterrows()):
 yy = y0 + (m - 2.5) * row_h
 lo, hi = row.r_CI95_low, row.r_CI95_high
 if not np.isnan(lo):
 ax.plot([lo, hi], [yy, yy], color=sp_colors[m], lw=1.3, alpha=0.75,
 solid_capstyle='round', zorder=2)
 ax.plot(row.r, yy, 'o', ms=5.2, color=sp_colors[m], zorder=3,
 markeredgecolor='white', markeredgewidth=0.5)

 ax.plot(pooled.r, y0, 'D', ms=10, color='black', zorder=5,
 markeredgecolor='white', markeredgewidth=0.9, label='Pooled (n=242)' if k == 0 else None)
 ax.plot(gl.r, y0 + 0.34, '^', ms=9.5, color='#B2182B', zorder=5,
 markeredgecolor='white', markeredgewidth=0.9,
 label='Gene-level (gene means)' if k == 0 else None)

 srow = sum_df.iloc[k]
 ax.text(0.80, y0 + 0.05,
```

```
 f"per-species mean r = {srow.PerSpecies_r_mean:+.3f}",
 ha='left', va='center', fontsize=9.5, style='italic',
 color='#333333', zorder=6)
 ax.text(0.80, y0 - 0.17,
 f"SD = {srow.PerSpecies_r_SD:.3f}, same direction {srow.N_species_same_direction}",
 ha='left', va='center', fontsize=9.5, style='italic',
 color='#333333', zorder=6)

ax.axvline(0, color='grey', lw=1.1, ls='--', zorder=1)
ax.set_yticks(ypos + 0.10)
ax.set_yticklabels(pair_labels, fontsize=11.5)
ax.set_xlabel('Pearson correlation coefficient (r) with 95% CI', fontsize=12)
ax.set_xlim(-0.72, 1.62)
ax.set_ylim(-0.75, len(pair_labels) - 0.25)
ax.tick_params(axis='x', labelsize=10.5)
ax.set_xticks(np.arange(-0.6, 0.81, 0.2))
ax.grid(axis='x', alpha=0.28, lw=0.7)
ax.set_axisbelow(True)
for s in ('top', 'right', 'left'):
 ax.spines[s].set_visible(False)
ax.legend(loc='lower left', fontsize=10, frameon=True, framealpha=0.95)
ax.set_title('Consistency of correlation estimates across the six species:\n'
 'effect sizes are stable, so pooling does not create the association',
 fontsize=13, fontweight='bold', pad=12)
fig2.text(0.5, 0.012,
 'Each coloured dot is one species (n = 39–41 genes); horizontal bars are 95% CIs '
 '(Fisher z). Black diamond = pooled analysis (n = 242 gene×species observations); '
 'red triangle = gene-level analysis using per-gene means across species (n = 41).',
 ha='center', fontsize=9.5, style='italic')
fig2.tight_layout(rect=[0, 0.045, 1, 1])

fig2_pdf = os.path.join(OUT_DIR, 'Fig_R2_cross_species_consistency.pdf')
fig2_png = os.path.join(OUT_DIR, 'Fig_R2_cross_species_consistency.png')
fig2.savefig(fig2_pdf, bbox_inches='tight')
fig2.savefig(fig2_png, dpi=300, bbox_inches='tight')
plt.close(fig2)
print(f' 2 (Cross-species consistency forest plot): {os.path.basename(fig2_pdf)}')

# ======================================================================
# ======================================================================
out_xlsx = os.path.join(OUT_DIR, 'per_species_correlation_results_v2.xlsx')
res_out = res_df.copy()
res_out['P_raw'] = res_out.P_raw.map(lambda x: f'{x:.3e}')
for c in ('P_FDR_within_level', 'P_FDR_global'):
 res_out[c] = res_out[c].map(lambda x: f'{x:.3e}')
for c in ('r', 'r_CI95_low', 'r_CI95_high'):
 res_out[c] = res_out[c].round(4)

sum_out = sum_df.copy()
for c in ('Pooled_P_raw', 'Pooled_P_FDR', 'GeneLevel_P_FDR'):
 sum_out[c] = sum_out[c].map(lambda x: f'{x:.3e}')

with pd.ExcelWriter(out_xlsx, engine='openpyxl') as w:
 res_out.to_excel(w, sheet_name='All_correlations', index=False)
 sum_out.to_excel(w, sheet_name='Consistency_summary', index=False)
 power_df.to_excel(w, sheet_name='Power_analysis', index=False)
 gene_level.round(4).to_excel(w, sheet_name='Gene_level_data', index=False)
 df.to_excel(w, sheet_name='Data_used_N242', index=False)

print(f' Results table saved: {os.path.basename(out_xlsx)}')
```

```
# ======================================================================
# 8. Summary
# ======================================================================
print()
print(SEP)
print('8. Summary')
print(SEP)
n_consistent = int((sum_df.Effect_size_consistent == 'Yes').sum())
n_samedir = sum(1 for v in sum_df.N_species_same_direction if v == '6/6')
print(f' (1) Data: manuscript parameter table directly {len(df)} ,,'
 f'and.')
print(f' (2) Per-species analysis (reviewer request a):6 ,'
 f' n = {min_n}~{int(counts.max())}.')
print(f' (3) Gene identity handling (reviewer request b): gene-level ,'
 f',(n = {len(gene_level)}),'
 f'explicitly removing gene-identity duplication.')
print(f' (4) Multiple testing correction (reviewer request c):Stratified BH-FDR + global
BH-FDR reported side by side.')
print(f' (5) Effect size robustness: {n_consistent}/6 r and r '
 f' < 0.10;{n_samedir}/6 pairs consistent across all 6 species.')
print(f' (6) Power note:N={min_n} N=, 80% power minimum detectable |r| |r| ≈
{r_min_detect:.3f};'
 f'Most target effects require N N ≈ 60 ,,'
 f'.')
print(SEP)
print(f'Output directory: {OUT_DIR}')
print(SEP)
```

Step 13. Optimal Codon Stability (R2 #4)

Cutoff sweep (5-20%), bootstrap (B=1000), leave-one-out (Table S9).

Input: filtered.fasta + ENC.xlsx

Output: Fig_R3 cutoff.pdf, Fig_R4 bootstrap.pdf, results_v2.xlsx

Method: Cutoff sweep; bootstrap B=1000; LOO

Source Code (step13_optimal_codon_stability.py):

```
# -*- coding: utf-8 -*-
"""
Optimal-codon stability analysis (Reviewer 2, Comment 4)
Optimal-codon stability analysis (Reviewer 2, Comment 4)

Reviewer concern: only ~ 40 genes per species, top/bottom 10% groups each contain ~ 4 ,
 deltaRSCU ;
 (a) ,(b) /,(c).

:
 : -- ENC 5_ENC_plot/Plot/ENC.xlsx(CodonW ,
 242 ,);RSCU *_CDS_filtered.fasta.
 : 3
 and 8 ,().
 (a) : 5/10/15/20/25%, deltaRSCU 0.04~0.20,
 (b) : 1000 , codons
 :/,
 (c) :None/,
 :/( 13)
```

```
: PDF(TrueType )+ PNG + Excel
"""

import os
from collections import defaultdict
from itertools import combinations
import numpy as np
import pandas as pd
import matplotlib
matplotlib.use('Agg')
import matplotlib.pyplot as plt
from Bio import SeqIO
# ---------------------------------------------------------------------------
# Repository configuration (ADDED for public release - see CHANGES.md)
# Only the hard-coded local paths were parameterised; no algorithm, threshold,
# formula, sort order or output content was modified.
# CUB_DATA_DIR : directory holding the input data (default:../data)
# CUB_OUT_DIR : directory for outputs (default:../output)
# ---------------------------------------------------------------------------
import os as _os
_HERE = _os.path.dirname(_os.path.abspath(__file__))
DATA_DIR = _os.environ.get("CUB_DATA_DIR", _os.path.normpath(_os.path.join(_HERE,
_os.pardir, "data")))
OUT_DIR = _os.environ.get("CUB_OUT_DIR", _os.path.normpath(_os.path.join(_HERE,
_os.pardir, "output")))
_os.makedirs(OUT_DIR, exist_ok=True)
# ---------------------------------------------------------------------------

matplotlib.rcParams['pdf.fonttype'] = 42
matplotlib.rcParams['ps.fonttype'] = 42
matplotlib.rcParams['font.family'] = 'Times New Roman'
matplotlib.rcParams['mathtext.fontset'] = 'stix'

WS = os.path.normpath(os.path.join(os.path.dirname(os.path.abspath(__file__)), os.pardir))
DATA = os.path.join(WS, "data")
ENC_XLSX = os.path.join(WS, "data", "ENC.xlsx")
OUT_DIR = os.environ.get("CUB_OUT_DIR", os.path.join(WS, "output"))
os.makedirs(OUT_DIR, exist_ok=True)

FASTA = {
 "C. lanatus": "watermelon_CDS_filtered.fasta",
 "C. melo": "melon_CDS_filtered.fasta",
 "C. sativus": "cucumber_CDS_filtered.fasta",
 "C. moschata": "pumpkin_CDS_filtered.fasta",
 "B. hispida": "winter melon_CDS_filtered.fasta",
 "M. charantia": "bitter melon_CDS_filtered.fasta",
}
SPECIES_ORDER = list(FASTA.keys())

TABLE3 = {
 "C. lanatus":
{'AAA','ACC','ACT','ATT','CAA','CAC','CGT','CTA','GAC','GCT','GGT','TCT','TTA','TTC'},
 "C. melo":
{'AAA','ACC','ACT','ATT','CAA','CGA','CGT','CTA','GAC','GCT','GGT','TCG','TCT','TGT','TTA'
,'TTC','TTG'},
 "C. sativus": {'AAC','ACT','ATT','CAA','CCG','CGT','CTT','GAC','GCT','GGT','GTT','TTC'},
 "C. moschata":
{'AAA','AAT','ACT','ATT','CAA','CAC','CGA','CGT','GAC','GCT','GGT','TAC','TCT','TTA','TTC'
},
```

```
 "B. hispida":
{'ACC','ACT','ATT','CAA','CAC','CGA','CGT','GAC','GCT','GGT','TCA','TCT','TTA','TTC'},
 "M. charantia": {'ACT','AGT','ATT','CAA','CGT','GAC','GCT','GGT','TTA','TTC'},
}
SHARED8 = ['ACT', 'ATT', 'CAA', 'CGT', 'GAC', 'GCT', 'GGT', 'TTC']

CODON_TABLE = {
 'TTT':'F','TTC':'F','TTA':'L','TTG':'L','TCT':'S','TCC':'S','TCA':'S','TCG':'S',
 'TAT':'Y','TAC':'Y','TAA':'*','TAG':'*','TGT':'C','TGC':'C','TGA':'*','TGG':'W',
 'CTT':'L','CTC':'L','CTA':'L','CTG':'L','CCT':'P','CCC':'P','CCA':'P','CCG':'P',
 'CAT':'H','CAC':'H','CAA':'Q','CAG':'Q','CGT':'R','CGC':'R','CGA':'R','CGG':'R',
 'ATT':'I','ATC':'I','ATA':'I','ATG':'M','ACT':'T','ACC':'T','ACA':'T','ACG':'T',
 'AAT':'N','AAC':'N','AAA':'K','AAG':'K','AGT':'S','AGC':'S','AGA':'R','AGG':'R',
 'GTT':'V','GTC':'V','GTA':'V','GTG':'V','GCT':'A','GCC':'A','GCA':'A','GCG':'A',
 'GAT':'D','GAC':'D','GAA':'E','GAG':'E','GGT':'G','GGC':'G','GGA':'G','GGG':'G',
}
ALL_CODONS = sorted(CODON_TABLE.keys())

SEP = '=' * 78


def calc_rscu_per_gene(seq):
 """ RSCU(and calc_codon_usage.py / generate_detailed_summary.py )."""
 s = str(seq).upper()
 codons = [s[i:i + 3] for i in range(0, len(s) - 2, 3)]
 valid = [c for c in codons if c in CODON_TABLE and CODON_TABLE[c] != '*']
 if len(valid) < 10:
 return None
 aa_cnt = defaultdict(lambda: defaultdict(int))
 aa_tot = defaultdict(int)
 for c in valid:
 aa = CODON_TABLE[c]
 aa_cnt[aa][c] += 1
 aa_tot[aa] += 1
 rscu = {}
 for aa in aa_tot:
 n_syn = len(aa_cnt[aa])
 if n_syn <= 1:
 continue
 for c, cnt in aa_cnt[aa].items():
 rscu[c] = (cnt / aa_tot[aa]) * n_syn
 return rscu


def pick_groups(gene_enc, pct):
 """Sort by ENC ascending; top pct = high-bias (low ENC), bottom pct = low-bias (high
ENC)."""
 ordered = sorted(gene_enc.items(), key=lambda kv: (kv[1], kv[0]))
 n = len(ordered)
 ng = max(1, int(n * pct))
 high = [g for g, _ in ordered[:ng]]
 low = [g for g, _ in ordered[-ng:]]
 return high, low


def optimal_codons(gene_enc, gene_rscu, pct=0.10, rscu_cut=1.00, delta_cut=0.08,
 high=None, low=None):
 """: RSCU > rscu_cut deltaRSCU >= delta_cut."""
 if high is None or low is None:
 high, low = pick_groups(gene_enc, pct)
```

```
 hr, lr = defaultdict(list), defaultdict(list)
 for g in high:
 for c, v in gene_rscu.get(g, {}).items():
 hr[c].append(v)
 for g in low:
 for c, v in gene_rscu.get(g, {}).items():
 lr[c].append(v)
 ha = {c: np.mean(v) for c, v in hr.items()}
 la = {c: np.mean(v) for c, v in lr.items()}
 out = set()
 for c in set(ha) | set(la):
 h, l = ha.get(c, 0.0), la.get(c, 0.0)
 if h > rscu_cut and (h - l) >= delta_cut:
 out.add(c)
 return out, high, low


# ============================================================================
# ============================================================================
print(SEP)
print('0. and')
print(SEP)
enc_df = pd.read_excel(ENC_XLSX)
gene_enc_all, gene_rscu_all, seqs = {}, {}, {}
for sp, fn in FASTA.items():
 fp = os.path.join(DATA, fn)
 sub = enc_df[enc_df.Species == sp]
 gene_enc_all[sp] = dict(zip(sub.Gene.astype(str), sub.ENC.astype(float)))
 gr = {}
 for rec in SeqIO.parse(fp, 'fasta'):
 r = calc_rscu_per_gene(rec.seq)
 if r:
 gr[rec.id] = r
 seqs[(sp, rec.id)] = rec.seq
 gene_rscu_all[sp] = gr
 common = set(gene_enc_all[sp]) & set(gr)
 print(f' {sp:<15} ENC={len(gene_enc_all[sp]):>3} RSCU={len(gr):>3} '
 f'={len(common):>3}')

baseline = {}
ok = True
for sp in SPECIES_ORDER:
 s, _, _ = optimal_codons(gene_enc_all[sp], gene_rscu_all[sp])
 baseline[sp] = s
 same = (s == TABLE3[sp])
 ok &= same
 print(f' {sp:<15} {len(s):>2} and3 {"" if same else ""}')
base_shared = set.intersection(*baseline.values())
print(f' : {len(base_shared)} -> {sorted(base_shared)}')
print(f' : {len(SHARED8)} -> {sorted(SHARED8)}')
if not ok or base_shared != set(SHARED8):
 raise SystemExit(':and,.')
print(' ,')

# ============================================================================
# ============================================================================
print()
print(SEP)
print('1. (5% / 10% / 15% / 20% / 25%)')
```

```
print(SEP)
PCTS = [0.05, 0.10, 0.15, 0.20, 0.25]
pct_rows, pct_sets = [], {}
for pct in PCTS:
 sets = {}
 for sp in SPECIES_ORDER:
 ge, gr = gene_enc_all[sp], gene_rscu_all[sp]
 high, low = pick_groups(ge, pct)
 s, _, _ = optimal_codons(ge, gr, high=high, low=low)
 sets[sp] = s
 shared = set.intersection(*sets.values())
 jac = len(s & baseline[sp]) / len(s | baseline[sp]) if (s or baseline[sp]) else 1.0
 pct_rows.append({
 'Group_pct': f'{int(pct*100)}%', 'Species': sp,
 'N_genes_total': len(ge), 'N_genes_per_group': len(high),
 'N_optimal': len(s),
 'Optimal_codons': ', '.join(sorted(s)),
 'High_bias_group': ', '.join(sorted(high)),
 'Low_bias_group': ', '.join(sorted(low)),
 'Jaccard_vs_baseline': round(jac, 4),
 })
 pct_sets[pct] = sets
 shared = set.intersection(*sets.values())
 print(f' {int(pct*100):>2}% ={len(pick_groups(gene_enc_all["C. lanatus"], pct)[0])} '
 f'={[len(sets[s]) for s in SPECIES_ORDER]} '
 f'={len(shared)} {sorted(shared)}')
pct_df = pd.DataFrame(pct_rows)

# ======================================================================
# ======================================================================
print()
print(SEP)
print('2. deltaRSCU (0.04 ~ 0.20, 10%)')
print(SEP)
DELTAS = [0.04, 0.06, 0.08, 0.10, 0.12, 0.15, 0.20]
delta_rows = []
for dc in DELTAS:
 sets = {}
 for sp in SPECIES_ORDER:
 s, _, _ = optimal_codons(gene_enc_all[sp], gene_rscu_all[sp], delta_cut=dc)
 sets[sp] = s
 delta_rows.append({
 'Delta_RSCU_cutoff': dc, 'Species': sp, 'N_optimal': len(s),
 'Optimal_codons': ', '.join(sorted(s)),
 'Jaccard_vs_baseline': round(
 len(s & baseline[sp]) / len(s | baseline[sp]) if (s or baseline[sp]) else 1.0, 4),
 })
 shared = set.intersection(*sets.values())
 print(f' deltaRSCU >= {dc:.2f} ={[len(sets[s]) for s in SPECIES_ORDER]} '
 f'={len(shared)}')
 delta_rows.append({
 'Delta_RSCU_cutoff': dc, 'Species': 'SHARED (intersection)',
 'N_optimal': len(shared), 'Optimal_codons': ', '.join(sorted(shared)),
 'Jaccard_vs_baseline': round(
 len(shared & set(SHARED8)) / len(shared | set(SHARED8)), 4),
 })
delta_df = pd.DataFrame(delta_rows)

# ======================================================================
```

```
# ======================================================================
print()
print(SEP)
print('3. (,B = 1000, seed = 42)')
print(SEP)
N_BOOT = 1000
RNG = np.random.default_rng(42)
boot = {}
for sp in SPECIES_ORDER:
 ge, gr = gene_enc_all[sp], gene_rscu_all[sp]
 genes = [g for g in ge if g in gr]
 enc_arr = np.array([ge[g] for g in genes])
 idx_pool = np.arange(len(genes))
 cnt = defaultdict(int)
 shared_cnt = 0
 n_opt_list = []
 for _ in range(N_BOOT):
 pick = RNG.choice(idx_pool, size=len(genes), replace=True)
 sub_enc = {genes[i]: float(enc_arr[i]) for i in pick}
 sub_rscu = {genes[i]: gr[genes[i]] for i in pick}
 if len(sub_enc) < 10:
 continue
 s, _, _ = optimal_codons(sub_enc, sub_rscu)
 n_opt_list.append(len(s))
 for c in s:
 cnt[c] += 1
 if set(SHARED8) <= s:
 shared_cnt += 1
 boot[sp] = {'counts': dict(cnt), 'all8_present': shared_cnt,
 'n_opt_mean': float(np.mean(n_opt_list)),
 'n_opt_ci': (float(np.percentile(n_opt_list, 2.5)),
 float(np.percentile(n_opt_list, 97.5)))}
 top = sorted(cnt.items(), key=lambda kv: -kv[1])[:10]
 print(f' {sp:<15} ={np.mean(n_opt_list):.1f} '
 f'(95%CI {np.percentile(n_opt_list, 2.5):.0f}-{np.percentile(n_opt_list, 97.5):.0f}) | '
 f'8={shared_cnt/N_BOOT*100:.1f}%')
 print(f' 10: '
 f'{", ".join(f"{c}: {v/N_BOOT*100:.0f}%" for c, v in top)}')

boot_rows = []
for sp in SPECIES_ORDER:
 for c in ALL_CODONS:
 f = boot[sp]['counts'].get(c, 0) / N_BOOT
 if f > 0 or c in baseline[sp]:
 boot_rows.append({
 'Species': sp, 'Codon': c, 'Amino_acid': CODON_TABLE[c],
 'Bootstrap_freq_pct': round(f * 100, 2),
 'In_manuscript_set': 'Yes' if c in baseline[sp] else 'No',
 'In_shared8': 'Yes' if c in SHARED8 else 'No',
 })
boot_df = pd.DataFrame(boot_rows)

# ======================================================================
# ======================================================================
print()
print(SEP)
print('4. (/)')
print(SEP)
loo_rows = []
```

```
for sp in SPECIES_ORDER:
 ge, gr = gene_enc_all[sp], gene_rscu_all[sp]
 high, low = pick_groups(ge, 0.10)
 base = baseline[sp]
 changed = 0
 trials = 0
 detail = []
 for grp_name, grp in (('high', high), ('low', low)):
 for g in grp:
 rest = [x for x in grp if x != g]
 if not rest:
 continue
 h2 = rest if grp_name == 'high' else high
 l2 = low if grp_name == 'high' else rest
 s, _, _ = optimal_codons(ge, gr, high=h2, low=l2)
 trials += 1
 same = (s == base)
 if not same:
 changed += 1
 detail.append(f'{grp_name}-{g}: {"+".join(sorted(s - base)) or "-"}'
 f'{"".join(sorted(base - s)) or ""}')
 loo_rows.append({
 'Species': sp, 'N_group_genes': len(high) + len(low),
 'N_LOO_trials': trials, 'N_sets_unchanged': trials - changed,
 'N_sets_changed': changed,
 'Pct_unchanged': round((trials - changed) / trials * 100, 1) if trials else np.nan,
 'Changed_cases': '; '.join(detail) if detail else 'none',
 })
 print(f' {sp:<15} {len(high)+len(low)} | {trials} | '
 f' {trials-changed}/{trials} '
 f'({(trials-changed)/trials*100:.1f}%)')
loo_df = pd.DataFrame(loo_rows)

# ======================================================================
# ======================================================================
print()
print(SEP)
print('5. /( 10%)and')
print(SEP)
group_rows = []
for sp in SPECIES_ORDER:
 ge, gr = gene_enc_all[sp], gene_rscu_all[sp]
 high, low = pick_groups(ge, 0.10)
 group_rows.append({
 'Species': sp, 'N_genes_total': len(ge),
 'N_per_group': len(high),
 'High_codon_bias_group_low_ENC': ', '.join(
 f'{g}({ge[g]:.2f})' for g in sorted(high, key=lambda x: ge[x])),
 'Low_codon_bias_group_high_ENC': ', '.join(
 f'{g}({ge[g]:.2f})' for g in sorted(low, key=lambda x: -ge[x])),
 })
 print(f' {sp:<15} ({len(high)}): '
 f'{", ".join(sorted(high))}')
 print(f' {"":<15} ({len(low)}): {", ".join(sorted(low))}')
group_df = pd.DataFrame(group_rows)

high_sets = {sp: set(pick_groups(gene_enc_all[sp], 0.10)[0]) for sp in SPECIES_ORDER}
low_sets = {sp: set(pick_groups(gene_enc_all[sp], 0.10)[1]) for sp in SPECIES_ORDER}
h_common = set.intersection(*high_sets.values())
```

```
l_common = set.intersection(*low_sets.values())
h_all = set.union(*high_sets.values())
l_all = set.union(*low_sets.values())
print(f'\n :6 {len(h_common)} {sorted(h_common)} | '
 f' {len(h_all)} ')
print(f' :6 {len(l_common)} {sorted(l_common)} | '
 f' {len(l_all)} ')
cons_rows = []
for c, s in (('High', h_all), ('Low', l_all)):
 for g in sorted(s):
 k = sum(1 for sp in SPECIES_ORDER if g in (high_sets[sp] if c == 'High' else
low_sets[sp]))
 cons_rows.append({'Group': f'{c}-codon-bias', 'Gene': g,
 'N_species_in_group': k,
 'Fraction': round(k / 6, 3),
 'In_all_six': 'Yes' if k == 6 else 'No'})
cons_df = pd.DataFrame(cons_rows)
print(f' 6: '
 f'{[r.Gene for r in cons_df.itertuples() if r.Group=="High-codon-bias" and
r.In_all_six=="Yes"]}')

# ======================================================================
# ======================================================================
print()
print(SEP)
print('6. Plot')
print(SEP)
SP_COLOR = {sp: c for sp, c in zip(SPECIES_ORDER,
 ['#E69F00', '#56B4E9', '#009E73', '#F0E442', '#D55E00', '#CC79A7'])}

fig, axes = plt.subplots(2, 2, figsize=(13.5, 10))

ax = axes[0, 0]
xs = [int(p * 100) for p in PCTS]
pct_labels = [f'{int(p*100)}%' for p in PCTS]
for sp in SPECIES_ORDER:
 ys = [int(pct_df[(pct_df.Group_pct == pl) & (pct_df.Species == sp)]
.N_optimal.iloc[0]) for pl in pct_labels]
 ax.plot(xs, ys, 'o-', color=SP_COLOR[sp], lw=1.9, ms=6, label=sp)
ax.axvline(10, color='grey', ls='--', lw=1.1)
ax.text(10.4, ax.get_ylim()[1] * 0.97, 'manuscript\n(10%)', fontsize=8.5,
 va='top', color='grey')
ax.set_xlabel('Size of high/low codon-bias groups (% of genes)', fontsize=11)
ax.set_ylabel('Number of optimal codons', fontsize=11)
ax.set_title('(a) Sensitivity to group-size cutoff', fontsize=12, fontweight='bold')
ax.legend(fontsize=8.6, ncol=2, frameon=True, framealpha=0.92)
ax.grid(alpha=0.28, lw=0.7)
ax.set_axisbelow(True)

ax = axes[0, 1]
shared_by_pct = [len(set.intersection(*pct_sets[p].values())) for p in PCTS]
ax.plot(xs, shared_by_pct, 'o-', color='#08519C', lw=2.2, ms=8)
for x, y in zip(xs, shared_by_pct):
 ax.annotate(str(y), (x, y), textcoords='offset points', xytext=(0, 9),
 ha='center', fontsize=10, fontweight='bold', color='#08519C')
ax.axhline(8, color='#B2182B', ls=':', lw=1.4)
ax.text(24.4, 8.15, 'manuscript value = 8', fontsize=9, color='#B2182B', ha='right')
ax.set_xlabel('Size of high/low codon-bias groups (% of genes)', fontsize=11)
ax.set_ylabel('Number of codons shared by all six species', fontsize=11)
```

```
ax.set_title('(b) Shared optimal codons vs group-size cutoff', fontsize=12,
fontweight='bold')
ax.set_ylim(0, max(shared_by_pct) + 2.5)
ax.grid(alpha=0.28, lw=0.7)
ax.set_axisbelow(True)

ax = axes[1, 0]
d_shared = delta_df[delta_df.Species == 'SHARED
(intersection)'].sort_values('Delta_RSCU_cutoff')
d_per = [int(np.mean(delta_df[(delta_df.Delta_RSCU_cutoff == dc) &
 (delta_df.Species != 'SHARED (intersection)')].N_optimal))
 for dc in DELTAS]
ax.plot(DELTAS, d_shared.N_optimal, 'o-', color='#08519C', lw=2.2, ms=8,
 label='Shared by all six species')
ax.plot(DELTAS, d_per, 's--', color='#B2182B', lw=1.8, ms=6.5,
 label='Mean per species')
ax.axvline(0.08, color='grey', ls='--', lw=1.1)
ax.text(0.0815, ax.get_ylim()[1] * 0.55, 'manuscript\n(0.08)', fontsize=8.5,
 va='center', color='grey')
ax.set_xlabel('deltaRSCU threshold', fontsize=11)
ax.set_ylabel('Number of optimal codons', fontsize=11)
ax.set_title('(c) Sensitivity to the deltaRSCU threshold', fontsize=12, fontweight='bold')
ax.legend(fontsize=9.5, frameon=True, framealpha=0.92)
ax.grid(alpha=0.28, lw=0.7)
ax.set_axisbelow(True)

ax = axes[1, 1]
w = 1.5
off = np.linspace(-w * 2.5, w * 2.5, 6)
for i, sp in enumerate(SPECIES_ORDER):
 ys = [float(pct_df[(pct_df.Group_pct == pl) & (pct_df.Species == sp)]
.Jaccard_vs_baseline.iloc[0]) * 100 for pl in pct_labels]
 ax.plot(np.array(xs) + off[i], ys, 'o-', color=SP_COLOR[sp], lw=1.8, ms=5.5, label=sp)
ax.axhline(100, color='grey', ls=':', lw=1.2)
ax.set_xlabel('Size of high/low codon-bias groups (% of genes)', fontsize=11)
ax.set_ylabel('Jaccard overlap with the manuscript set (%)', fontsize=11)
ax.set_title('(d) Overlap of per-species optimal codons with the manuscript result',
 fontsize=12, fontweight='bold')
ax.legend(fontsize=8.6, ncol=2, frameon=True, framealpha=0.92)
ax.set_ylim(0, 108)
ax.grid(alpha=0.28, lw=0.7)
ax.set_axisbelow(True)

fig.suptitle('Stability of optimal-codon identification to alternative cutoffs '
 '(Reviewer 2, Comment 4)', fontsize=14, fontweight='bold', y=0.985)
fig.tight_layout(rect=[0, 0, 1, 0.965])
f1_pdf = os.path.join(OUT_DIR, 'Fig_R3_optimal_codon_cutoff_sensitivity.pdf')
f1_png = os.path.join(OUT_DIR, 'Fig_R3_optimal_codon_cutoff_sensitivity.png')
fig.savefig(f1_pdf, bbox_inches='tight')
fig.savefig(f1_png, dpi=300, bbox_inches='tight')
plt.close(fig)
print(f' 1 : {os.path.basename(f1_pdf)}')

# ======================================================================
# ======================================================================
codon_order = SHARED8 + sorted({c for sp in SPECIES_ORDER for c in baseline[sp]} -
set(SHARED8))
mat = np.full((len(codon_order), 6), np.nan)
for j, sp in enumerate(SPECIES_ORDER):
```

```
 for i, c in enumerate(codon_order):
 mat[i, j] = boot[sp]['counts'].get(c, 0) / N_BOOT * 100

fig2, ax2 = plt.subplots(figsize=(9.6, 0.30 * len(codon_order) + 2.6))
im = ax2.imshow(mat, cmap='YlGnBu', vmin=0, vmax=100, aspect='auto')
ax2.set_xticks(range(6))
ax2.set_xticklabels([sp.replace(' ', '\n') for sp in SPECIES_ORDER], fontsize=9.5)
ax2.set_yticks(range(len(codon_order)))
ylab = [f'{c} ({CODON_TABLE[c]})' for c in codon_order]
ax2.set_yticklabels(ylab, fontsize=9.5)
for i, c in enumerate(codon_order):
 if i < len(SHARED8):
 ax2.get_yticklabels()[i].set_fontweight('bold')
 ax2.get_yticklabels()[i].set_color('#B2182B')
for i in range(len(codon_order)):
 for j in range(6):
 v = mat[i, j]
 if not np.isnan(v):
 ax2.text(j, i, f'{v:.0f}', ha='center', va='center', fontsize=8.4,
 color='white' if v > 60 else '#222222')
ax2.axhline(len(SHARED8) - 0.5, color='#B2182B', lw=1.6)
ax2.text(-0.62, len(SHARED8) / 2 - 0.5, 'shared by\nall six\nspecies', fontsize=9,
 color='#B2182B', ha='center', va='center', fontweight='bold',
 transform=ax2.get_yaxis_transform(), clip_on=False)
ax2.text(-0.62, len(SHARED8) + (len(codon_order) - len(SHARED8)) / 2 - 0.5,
 'species-specific\nor partial', fontsize=9, color='#444444', ha='center',
 va='center', transform=ax2.get_yaxis_transform(), clip_on=False)
cb = fig2.colorbar(im, ax=ax2, shrink=0.62, pad=0.035)
cb.set_label('Bootstrap selection frequency (%)', fontsize=10)
ax2.set_title('Bootstrap stability of optimal codons (B = 1000 gene resamples)\n'
 'Bold red labels = the eight codons shared by all six species in the manuscript',
 fontsize=12, fontweight='bold', pad=12)
fig2.tight_layout()
f2_pdf = os.path.join(OUT_DIR, 'Fig_R4_optimal_codon_bootstrap_stability.pdf')
f2_png = os.path.join(OUT_DIR, 'Fig_R4_optimal_codon_bootstrap_stability.png')
fig2.savefig(f2_pdf, bbox_inches='tight')
fig2.savefig(f2_png, dpi=300, bbox_inches='tight')
plt.close(fig2)
print(f' 2 : {os.path.basename(f2_pdf)}')

# ======================================================================
# ======================================================================
boot8_rows = []
for sp in SPECIES_ORDER:
 for c in SHARED8:
 boot8_rows.append({
 'Species': sp, 'Codon': c, 'Amino_acid': CODON_TABLE[c],
 'Bootstrap_freq_pct': round(boot[sp]['counts'].get(c, 0) / N_BOOT * 100, 2),
 'All8_retained_pct': round(boot[sp]['all8_present'] / N_BOOT * 100, 2),
 'N_optimal_mean': round(boot[sp]['n_opt_mean'], 2),
 'N_optimal_CI95': f'{boot[sp]["n_opt_ci"][0]:.0f}-{boot[sp]["n_opt_ci"][1]:.0f}',
 })
boot8_df = pd.DataFrame(boot8_rows)

out_xlsx = os.path.join(OUT_DIR, 'optimal_codon_stability_results_v2.xlsx')
with pd.ExcelWriter(out_xlsx, engine='openpyxl') as w:
 pct_df.to_excel(w, sheet_name='1_group_size_sensitivity', index=False)
 delta_df.to_excel(w, sheet_name='2_deltaRSCU_sensitivity', index=False)
 boot8_df.to_excel(w, sheet_name='3_bootstrap_shared8', index=False)
```

```
    boot_df.to_excel(w, sheet_name='3_bootstrap_all_codons', index=False)
    loo_df.to_excel(w, sheet_name='4_leave_one_out', index=False)
    group_df.to_excel(w, sheet_name='5_high_low_group_lists', index=False)
    cons_df.to_excel(w, sheet_name='5_group_conservation', index=False)
print(f' Results table saved: {os.path.basename(out_xlsx)}')

# ======================================================================
# 9. Summary
# ======================================================================
print()
print(SEP)
print('9. Summary')
print(SEP)
print(f' (1) :and3,'
 f' {len(base_shared)} and.')
print(f' (2) 5%~25%: = '
 f'{dict(zip([f"{int(p*100)}%" for p in PCTS], shared_by_pct))};'
 f'(10%,8).')
jac_min = pct_df[pct_df.Group_pct != '10%'].Jaccard_vs_baseline.min()
print(f' (3) and Jaccard {jac_min*100:.1f}%.')
print(f' (4) deltaRSCU 0.04~0.20: = '
 f'{list(zip([f"{d:.2f}" for d in DELTAS], d_shared.N_optimal.tolist()))}.')
b8 = boot8_df.Bootstrap_freq_pct
print(f' (5) (B=1000):8 {b8.min():.1f}%~{b8.max():.1f}%,'
 f' {b8.median():.1f}%.')
all8 = boot8_df.groupby('Species').All8_retained_pct.mean()
print(f' 8 codons:'
 f'{", ".join(f"{sp}={v:.0f}%" for sp, v in all8.items())}')
unch = loo_df.Pct_unchanged
print(f' (6) : '
 f'{unch.min():.1f}%~{unch.max():.1f}%( {unch.mean():.1f}%).')
print(f' (7) : 6 '
 f'{sorted(h_common)}; {sorted(l_common)}.')
print(SEP)
print(f'Output directory: {OUT_DIR}')
print(SEP)
```

Step 14. Filter Sensitivity (R2 #8)

Nested comparison A/B/S0; CodonW batch; shared codon analysis (Table S8).

Input: raw.fasta + filtered + ENC.xlsx

Output: Fig_R5 sensitivity.pdf, Fig_R6 shared codons.pdf, results_v2.xlsx

Method: Nested A/B/S0; CodonW batch

Source Code (step14_filter_sensitivity.py):

```
# -*- coding: utf-8 -*-
"""
Filter sensitivity analysis v3(2,8)

Fixed v2 two issues:
 (1) B level previously from.gb "dedup-then-filter", vs manuscript filter_cds.py"filter-
then-dedup",
 , B(35~38) < A(41) paradox.
 v3 Changed to read raw *_CDS.fasta(106~112 ,),
 filter_cds.py , ATG ATG/GTG/TTG,
 B A.
```

```
 (2) stats 'dup' KeyError;.get().

( A B S0):
 A :ATG + >=300 bp + (= 2_/filter_cds.py)
 B :ATG/GTG/TTG + >=300 bp + (= Table S7 B)
 S0 : CDS,( all valid annotated CDSs)
 ( A ),"same name different sequence".

:
 - A ENC 5_ENC_plot/ENC.xlsx(CodonW ,), and 242 ;
 - B/S0 A CodonW 1.4.2 ENC(,);
 - GC1/GC2/GC3 and RSCU (and);
 - and: RSCU > 1 deltaRSCU >= 0.08( 10%).
"""

import os
import subprocess
import tempfile
from collections import defaultdict
import numpy as np
import pandas as pd
import matplotlib
matplotlib.use('Agg')
import matplotlib.pyplot as plt
from Bio import SeqIO
# ----------------------------------------------------------------------------
# Repository configuration (ADDED for public release - see CHANGES.md)
# Only the hard-coded local paths were parameterised; no algorithm, threshold,
# formula, sort order or output content was modified.
# CUB_DATA_DIR : directory holding the input data (default:../data)
# CUB_OUT_DIR : directory for outputs (default:../output)
# ----------------------------------------------------------------------------
import os as _os
_HERE = _os.path.dirname(_os.path.abspath(__file__))
DATA_DIR = _os.environ.get("CUB_DATA_DIR", _os.path.normpath(_os.path.join(_HERE,
_os.pardir, "data")))
OUT_DIR = _os.environ.get("CUB_OUT_DIR", _os.path.normpath(_os.path.join(_HERE,
_os.pardir, "output")))
_os.makedirs(OUT_DIR, exist_ok=True)
# ----------------------------------------------------------------------------

matplotlib.rcParams['pdf.fonttype'] = 42
matplotlib.rcParams['ps.fonttype'] = 42
matplotlib.rcParams['font.family'] = 'Times New Roman'
matplotlib.rcParams['mathtext.fontset'] = 'stix'

WS = os.path.normpath(os.path.join(os.path.dirname(os.path.abspath(__file__)), os.pardir))
RAW_DIR = os.path.join(WS, "data")
DATA = os.path.join(WS, "data")
ENC_XLSX = os.path.join(WS, "data", "ENC.xlsx")
OUT_DIR = os.environ.get("CUB_OUT_DIR", os.path.join(WS, "output"))
os.makedirs(OUT_DIR, exist_ok=True)
CODONW = os.environ.get("CUB_CODONW_EXE", os.path.join(WS, "external", "CodonW.exe"))

SPECIES_ORDER = ["C. lanatus", "C. melo", "C. sativus",
 "C. moschata", "B. hispida", "M. charantia"]
RAW_FASTA = {
 "C. lanatus": "watermelon_CDS.fasta", "C. melo": "melon_CDS.fasta",
 "C. sativus": "cucumber_CDS.fasta", "C. moschata": "pumpkin_CDS.fasta",
 "B. hispida": "winter melon_CDS.fasta",
```

```
 "M. charantia": "bitter melon_CDS.fasta",
}
FILTERED_FASTA = {
 "C. lanatus": "watermelon_CDS_filtered.fasta", "C. melo": "melon_CDS_filtered.fasta",
 "C. sativus": "cucumber_CDS_filtered.fasta", "C. moschata": "pumpkin_CDS_filtered.fasta",
 "B. hispida": "winter melon_CDS_filtered.fasta",
 "M. charantia": "bitter melon_CDS_filtered.fasta",
}
ALT_STARTS = {'ATG', 'GTG', 'TTG'}
CODON_TABLE = {
 'TTT':'F','TTC':'F','TTA':'L','TTG':'L','TCT':'S','TCC':'S','TCA':'S','TCG':'S',
 'TAT':'Y','TAC':'Y','TAA':'*','TAG':'*','TGT':'C','TGC':'C','TGA':'*','TGG':'W',
 'CTT':'L','CTC':'L','CTA':'L','CTG':'L','CCT':'P','CCC':'P','CCA':'P','CCG':'P',
 'CAT':'H','CAC':'H','CAA':'Q','CAG':'Q','CGT':'R','CGC':'R','CGA':'R','CGG':'R',
 'ATT':'I','ATC':'I','ATA':'I','ATG':'M','ACT':'T','ACC':'T','ACA':'T','ACG':'T',
 'AAT':'N','AAC':'N','AAA':'K','AAG':'K','AGT':'S','AGC':'S','AGA':'R','AGG':'R',
 'GTT':'V','GTC':'V','GTA':'V','GTG':'V','GCT':'A','GCC':'A','GCA':'A','GCG':'A',
 'GAT':'D','GAC':'D','GAA':'E','GAG':'E','GGT':'G','GGC':'G','GGA':'G','GGG':'G',
}
SEP = '=' * 78


def calc_gc123(seq):
 s = str(seq).upper()
 cs = [s[i:i+3] for i in range(0, len(s)-2, 3) if len(s[i:i+3]) == 3]
 if len(cs) < 5:
 return None
 n = len(cs)
 return (sum(1 for c in cs if c[0] in 'GC')/n,
 sum(1 for c in cs if c[1] in 'GC')/n,
 sum(1 for c in cs if c[2] in 'GC')/n)


def calc_rscu(seq):
 s = str(seq).upper()
 cs = [s[i:i+3] for i in range(0, len(s)-2, 3)]
 v = [c for c in cs if c in CODON_TABLE and CODON_TABLE[c] != '*']
 if len(v) < 10:
 return None
 ac = defaultdict(lambda: defaultdict(int)); at = defaultdict(int)
 for c in v:
 a = CODON_TABLE[c]; ac[a][c] += 1; at[a] += 1
 r = {}
 for a in at:
 n = len(ac[a])
 if n <= 1:
 continue
 for c, k in ac[a].items():
 r[c] = (k/at[a])*n
 return r


def pick_first(records, starts, min_len):
 """ filter_cds.py:,->->, (dict, stats)."""
 kept, seen = {}, set()
 st = defaultdict(int)
 for g, s in records:
 st['total'] += 1
 if s[:3] not in starts:
 st['start'] += 1
```

```
continue
if len(s) < min_len:
st['len'] += 1
continue
if g in seen:
st['dup'] += 1
continue
seen.add(g)
kept[g] = s
st['kept'] += 1
return kept, dict(st)


def codonw_enc(seqs, tag):
if not seqs:
return {}, set()
work = os.path.join(tempfile.gettempdir(), 'cw_c8c', tag)
os.makedirs(work, exist_ok=True)
out_t, blk_t = os.path.join(work, 'out.txt'), os.path.join(work, 'blk.txt')
with open(os.path.join(work, 'in.fasta'), 'w') as f:
for g, s in seqs.items():
f.write(f'>{g}\n{s}\n')
for p in (out_t, blk_t):
if os.path.exists(p):
os.remove(p)
subprocess.run([CODONW, 'in.fasta', 'out.txt', 'blk.txt', '-nomenu',
'-silent', '-enc'], cwd=work, capture_output=True,
text=True, check=True)
lines = [l for l in open(out_t).read().splitlines() if l.strip()]
hdr = [h.strip() for h in lines[0].split('\t')]
iN = hdr.index('Nc')
res = {}
for l in lines[1:]:
parts = l.rstrip('\t').split('\t')
if len(parts) > iN:
try:
res[parts[0].strip()] = float(parts[iN])
except ValueError:
pass
return res, set(seqs) - set(res)


def optimal_codons(gene_enc, gene_rscu, pct=0.10, rscu_cut=1.0, d_cut=0.08):
common = [g for g in gene_rscu if gene_enc.get(g) is not None]
if len(common) < 10:
return set(), 0
o = sorted(((g, gene_enc[g]) for g in common), key=lambda kv: (kv[1], kv[0]))
ng = max(1, int(len(o)*pct))
high = [g for g, _ in o[:ng]]; low = [g for g, _ in o[-ng:]]
hr, lr = defaultdict(list), defaultdict(list)
for g in high:
for c, v in gene_rscu[g].items(): hr[c].append(v)
for g in low:
for c, v in gene_rscu[g].items(): lr[c].append(v)
ha = {c: np.mean(v) for c, v in hr.items()}
la = {c: np.mean(v) for c, v in lr.items()}
opt = {c for c in set(ha)|set(la)
if ha.get(c, 0) > rscu_cut and (ha.get(c, 0)-la.get(c, 0)) >= d_cut}
return opt, ng
```

```
# ====================================================================
print(SEP); print('0. (, A B S0)'); print(SEP)
enc_df = pd.read_excel(ENC_XLSX)
ref_enc = {(r.Species, str(r.Gene)): float(r.ENC) for r in enc_df.itertuples()}

RAW, SETS, STATS, ENC, NOENC = {}, {}, {}, {}, defaultdict(list)
for sp in SPECIES_ORDER:
 recs = [(r.id, str(r.seq).upper())
 for r in SeqIO.parse(os.path.join(RAW_DIR, RAW_FASTA[sp]), 'fasta')]
 RAW[sp] = recs
 manuscript = {r.id: str(r.seq).upper() for r in
 SeqIO.parse(os.path.join(DATA, FILTERED_FASTA[sp]), 'fasta')}

 A, stA = pick_first(recs, {'ATG'}, 300)
 B, stB = pick_first(recs, ALT_STARTS, 300)

 assert set(A) == set(manuscript), f'{sp}: A≠FASTA'
 assert all(A[g] == manuscript[g] for g in A), f'{sp}: A≠FASTA'
 ref_names = {g for (s2, g) in ref_enc if s2 == sp}
 assert set(A) == ref_names, f'{sp}: A≠ENC'
 assert set(A) <= set(B), f'{sp}: A B '

 S0 = {}
 for g, s in recs:
 if g not in S0:
 S0[g] = s
 for g in S0:
 if g in A:
 S0[g] = A[g]
 elif g in B:
 S0[g] = B[g]
 assert set(B) <= set(S0), f'{sp}: B S0 '

 SETS[sp] = {'A': A, 'B': B, 'S0': S0}
 STATS[sp] = {'A': stA, 'B': stB}
 ENC[sp] = {g: ref_enc[(sp, g)] for g in A}
 print(f' {sp:<15} ={len(recs):>3} A={len(A):>3}(andFASTA+ENC)'
 f' B={len(B):>3}(BA) S0={len(S0):>3}(S0B)')
 print(f' A: ATG={stA["start"]} <300bp={stA["len"]} ={stA.get("dup", 0)}')
 print(f' B: ATG/GTG/TTG={stB["start"]} <300bp={stB["len"]} '
 f'={stB.get("dup", 0)}')

print(); print(' ENC(CodonW 1.4.2,,A 242 )...')
for sp in SPECIES_ORDER:
 for tag in ('B', 'S0'):
 new = {g: s for g, s in SETS[sp][tag].items() if g not in ENC[sp]}
 if new:
 got, miss = codonw_enc(new, f'{sp}_{tag}'.replace(' ', '_').replace('.', ''))
 ENC[sp].update(got)
 for g in sorted(miss):
 NOENC[sp].append((tag, g, len(SETS[sp][tag][g])))
for sp in SPECIES_ORDER:
 if NOENC[sp]:
 uniq = sorted({(g, n) for _, g, n in NOENC[sp]})
 print(f' {sp:<15} CodonW (, and): '
 f'{", ".join(f"{g}({n}bp)" for g, n in uniq)}')

# ====================================================================
```

```
print(); print(SEP); print('1. and'); print(SEP)
rows, OPT, NG = [], {}, {}
for sp in SPECIES_ORDER:
 OPT[sp], NG[sp] = {}, {}
 for tag in ('A', 'B', 'S0'):
 gs = SETS[sp][tag]
 rscu, gcs, encs, codons = {}, [], [], []
 for g, s in gs.items():
 gc = calc_gc123(s)
 if gc:
 gcs.append(gc)
 r = calc_rscu(s)
 if r:
 rscu[g] = r
 e = ENC[sp].get(g)
 if e is not None:
 encs.append(e)
 codons.append(len(s)//3)
 opt, ng = optimal_codons(ENC[sp], rscu)
 OPT[sp][tag], NG[sp][tag] = opt, ng
 gcs = np.array(gcs)
 rows.append({
 'Species': sp, 'Set': tag, 'N_genes': len(gs),
 'N_genes_with_ENC': len(encs), 'N_per_extreme_group': ng,
 'Mean_GC1': round(float(gcs[:, 0].mean()), 4),
 'Mean_GC2': round(float(gcs[:, 1].mean()), 4),
 'Mean_GC3': round(float(gcs[:, 2].mean()), 4),
 'Mean_GCall': round(float(gcs.mean()), 4),
 'Mean_ENC': round(float(np.mean(encs)), 2) if encs else None,
 'Median_gene_codons': int(np.median(codons)),
 'N_optimal': len(opt), 'Optimal_codons': ', '.join(sorted(opt)),
 })
comp = pd.DataFrame(rows)
for sp in SPECIES_ORDER:
 line = f' {sp:<15}'
 for tag in ('A', 'B', 'S0'):
 r = comp[(comp.Species == sp) & (comp.Set == tag)].iloc[0]
 line += (f' | {tag}: N={r.N_genes:>3} GC3={r.Mean_GC3:.4f} '
 f'ENC={r.Mean_ENC} opt={r.N_optimal}')
 print(line)
 for tag in ('A', 'B', 'S0'):
 print(f' {tag:>2} : {", ".join(sorted(OPT[sp][tag]))}')

shared = {t: set.intersection(*[OPT[sp][t] for sp in SPECIES_ORDER])
 for t in ('A', 'B', 'S0')}
core = sorted(set.intersection(*shared.values()))
print()
for t in ('A', 'B', 'S0'):
 print(f' {t:>2}: {len(shared[t])} {sorted(shared[t])}')
print(f' (): {len(core)} {core}')

print()
print(' (Jaccard):')
jac_rows = []
for sp in SPECIES_ORDER:
 for t2 in ('B', 'S0'):
 a, b = OPT[sp]['A'], OPT[sp][t2]
 j = len(a & b)/len(a | b) if (a or b) else 1.0
 jac_rows.append({'Species': sp, 'Comparison': f'A vs {t2}',
```

```
 'N_A': len(a), f'N_{t2}': len(b),
 'N_common': len(a & b), 'Jaccard': round(j, 3)})
 print(f' {sp:<15} A({len(a)}) vs {t2}({len(b)}): {len(a & b)},'
 f'Jaccard={j:.2f}')
jac_df = pd.DataFrame(jac_rows)

exc = []
for sp in SPECIES_ORDER:
 for g, s in SETS[sp]['S0'].items():
 if g in SETS[sp]['A']:
 continue
 gc = calc_gc123(s)
 exc.append({'Species': sp, 'Gene': g, 'Length_bp': len(s),
 'Codons': len(s)//3, 'Start_codon': s[:3],
 'In_set_B': 'Yes' if g in SETS[sp]['B'] else 'No',
 'GC3': round(gc[2], 4) if gc else None,
 'ENC': ENC[sp].get(g)})
exc_df = pd.DataFrame(exc)
ret = [(len(s), calc_gc123(s)) for sp in SPECIES_ORDER for s in SETS[sp]['A'].values()]
ret_len = [n for n, _ in ret]
ret_gc3 = [gc[2] for _, gc in ret if gc]
print()
print(f' A ( S0 A): {len(exc_df)} ')
print(f' {exc_df.Length_bp.median():.0f} bp({exc_df.Codons.median():.0f} )'
 f' vs {np.median(ret_len):.0f} bp')
print(f' GC3 {exc_df.GC3.mean():.4f} vs {np.mean(ret_gc3):.4f}'
 f'( {abs(exc_df.GC3.mean()-np.mean(ret_gc3)):.4f})')
print(f' ATG {int((exc_df.Start_codon != "ATG").sum())} ,'
 f' GC3 {exc_df[exc_df.Start_codon != "ATG"].GC3.mean():.4f}')
print(f' 300 bp {int((exc_df.Length_bp < 300).sum())} ,'
 f' GC3 {exc_df[exc_df.Length_bp < 300].GC3.mean():.4f}')

# ======================================================================
print(); print(SEP); print('2. Plot'); print(SEP)
SET_COLOR = {'A': '#08519C', 'B': '#4DAF4A', 'S0': '#B2182B'}
SET_LBL = {'A': 'A: manuscript filters\n(ATG, >=300 bp)',
 'B': 'B: relaxed start codon\n(ATG/GTG/TTG, >=300 bp)',
 'S0': 'S0: all annotated CDSs\n(no start/length filter)'}
fig, axes = plt.subplots(2, 2, figsize=(14, 10))
x = np.arange(6); w = 0.26
xtl = [s.replace(' ', '\n') for s in SPECIES_ORDER]

ax = axes[0, 0]
for k, t in enumerate(('A', 'B', 'S0')):
 ys = [len(SETS[sp][t]) for sp in SPECIES_ORDER]
 ax.bar(x + (k-1)*w, ys, w, color=SET_COLOR[t], label=SET_LBL[t])
 for xi, v in zip(x + (k-1)*w, ys):
 ax.text(xi, v + 1.2, str(v), ha='center', fontsize=8.5)
ax.set_xticks(x); ax.set_xticklabels(xtl, fontsize=9)
ax.set_ylabel('Number of genes (observations)', fontsize=11)
ax.set_title('(a) Observations retained under the three filtering levels',
 fontsize=12, fontweight='bold')
ax.legend(fontsize=8.4, loc='upper left')
ax.grid(axis='y', alpha=0.28, lw=0.7); ax.set_axisbelow(True)

ax = axes[0, 1]
for k, t in enumerate(('A', 'B', 'S0')):
 ys = [comp[(comp.Species == sp) & (comp.Set == t)].Mean_GC3.iloc[0]
 for sp in SPECIES_ORDER]
```

```
 ax.bar(x + (k-1)*w, ys, w, color=SET_COLOR[t], label=SET_LBL[t])
ax.set_xticks(x); ax.set_xticklabels(xtl, fontsize=9)
ax.set_ylabel('Mean GC3', fontsize=11)
ax.set_ylim(0.24, 0.345)
ax.set_title('(b) Mean GC3 shifts upward as the filters are relaxed',
 fontsize=12, fontweight='bold')
ax.legend(fontsize=8.4)
ax.grid(axis='y', alpha=0.28, lw=0.7); ax.set_axisbelow(True)

ax = axes[1, 0]
for k, t in enumerate(('A', 'B', 'S0')):
 ys = [len(OPT[sp][t]) for sp in SPECIES_ORDER]
 ax.plot(x + (k-1)*w, ys, 'o-', color=SET_COLOR[t], lw=1.9, ms=6, label=SET_LBL[t])
ax.set_xticks(x); ax.set_xticklabels(xtl, fontsize=9)
ax.set_ylabel('Number of optimal codons', fontsize=11)
ax.set_title('(c) Optimal codons per species under the three levels',
 fontsize=12, fontweight='bold')
ax.legend(fontsize=8.4)
ax.grid(alpha=0.28, lw=0.7); ax.set_axisbelow(True)

ax = axes[1, 1]
bins = np.arange(0, 7200, 150)
ax.hist([len(s) for sp in SPECIES_ORDER for s in SETS[sp]['A'].values()],
 bins=bins, color=SET_COLOR['A'], alpha=0.82, label='Retained in set A')
ax.hist(exc_df.Length_bp.dropna(), bins=bins, color=SET_COLOR['S0'], alpha=0.62,
 label='Excluded by the manuscript filters')
ax.axvline(300, color='black', ls='--', lw=1.3)
ax.text(360, ax.get_ylim()[1]*0.93, '300 bp filter', fontsize=9)
ax.set_xlabel('CDS length (bp)', fontsize=11)
ax.set_ylabel('Number of genes', fontsize=11)
ax.set_title('(d) Length distribution: retained vs excluded CDSs',
 fontsize=12, fontweight='bold')
ax.legend(fontsize=9.5)
ax.grid(alpha=0.28, lw=0.7); ax.set_axisbelow(True)

fig.suptitle('Sensitivity of codon-usage results to the CDS filtering criteria '
 '(Reviewer 2, Comment 8)', fontsize=14, fontweight='bold', y=0.99)
fig.tight_layout(rect=[0, 0, 1, 0.962])
f1 = os.path.join(OUT_DIR, 'Fig_R5_filter_sensitivity.pdf')
fig.savefig(f1, bbox_inches='tight')
fig.savefig(os.path.join(OUT_DIR, 'Fig_R5_filter_sensitivity.png'), dpi=300,
 bbox_inches='tight')
plt.close(fig)
print(f' 1 : {os.path.basename(f1)}')

fig2, ax2 = plt.subplots(figsize=(8.4, 5.2))
tags = ['A', 'B', 'S0']
vals = [len(shared[t]) for t in tags]
bars = ax2.bar(tags, vals, color=[SET_COLOR[t] for t in tags], width=0.5)
for b, v, t in zip(bars, vals, tags):
 ax2.text(b.get_x()+b.get_width()/2, v+0.3, str(v), ha='center',
 fontsize=14, fontweight='bold')
ax2.set_xticklabels([SET_LBL[t] for t in tags], fontsize=9.5)
ax2.set_ylabel('Number of codons shared by all six species', fontsize=11)
ax2.set_title('Shared optimal codons under progressively relaxed filters\n'
 f'Codons shared at every level (robust core): {", ".join(core)}',
 fontsize=12.5, fontweight='bold', pad=12)
ax2.set_ylim(0, max(vals)+4.5)
ax2.text(0.5, 0.86, f'Robust core across all three levels:\n{", ".join(core)}',
```

```
 transform=ax2.transAxes, ha='center', fontsize=11,
 bbox=dict(boxstyle='round, pad=0.55', fc='#FFF3D6', ec='#B2182B', lw=1.3))
ax2.grid(axis='y', alpha=0.28, lw=0.7); ax2.set_axisbelow(True)
fig2.tight_layout()
f2 = os.path.join(OUT_DIR, 'Fig_R6_shared_codons_vs_filters.pdf')
fig2.savefig(f2, bbox_inches='tight')
fig2.savefig(os.path.join(OUT_DIR, 'Fig_R6_shared_codons_vs_filters.png'), dpi=300,
 bbox_inches='tight')
plt.close(fig2)
print(f' 2 : {os.path.basename(f2)}')

out_xlsx = os.path.join(OUT_DIR, 'filter_sensitivity_results_v2.xlsx')
with pd.ExcelWriter(out_xlsx, engine='openpyxl') as wr:
 comp.to_excel(wr, sheet_name='1_three_level_comparison', index=False)
 pd.DataFrame([{'Set': t, 'Definition': SET_LBL[t].replace('\n', ' '),
 'N_shared': len(shared[t]),
 'Shared_codons': ', '.join(sorted(shared[t]))}
 for t in tags]).to_excel(wr, sheet_name='2_shared_codons', index=False)
 pd.DataFrame([{'Level': 'Robust core (all three)', 'N': len(core),
 'Codons': ', '.join(core)}]).to_excel(
 wr, sheet_name='2b_robust_core', index=False)
 jac_df.to_excel(wr, sheet_name='3_per_species_overlap', index=False)
 exc_df.to_excel(wr, sheet_name='4_genes_excluded_from_A', index=False)
 pd.DataFrame([{'Species': sp, 'Raw_CDS_records': len(RAW[sp]),
 'A_failed_start': STATS[sp]['A']['start'],
 'A_failed_length': STATS[sp]['A']['len'],
 'A_failed_duplicate': STATS[sp]['A'].get('dup', 0),
 'A_kept': STATS[sp]['A']['kept'],
 'B_failed_start': STATS[sp]['B']['start'],
 'B_failed_length': STATS[sp]['B']['len'],
 'B_failed_duplicate': STATS[sp]['B'].get('dup', 0),
 'B_kept': STATS[sp]['B']['kept'],
 'S0_kept': len(SETS[sp]['S0'])} for sp in SPECIES_ORDER]
 ).to_excel(wr, sheet_name='5_filter_stats', index=False)
 pd.DataFrame([{'Species': sp, 'Gene': g, 'Length_bp': n,
 'Reason': 'too short for CodonW ENC; excluded from ENC ranking'}
 for sp in SPECIES_ORDER for g, n in sorted({(g, n) for _, g, n in NOENC[sp]})]
 ).to_excel(wr, sheet_name='6_no_ENC_genes', index=False)
print(f' Results table saved: {os.path.basename(out_xlsx)}')

print(); print(SEP); print('3. Summary'); print(SEP)
print(f' (1) A and 242 (and).')
print(f' (2) A B S0;.')
print(f' (3) :A={min(len(SETS[s]["A"]) for s in SPECIES_ORDER)}-'
 f'{max(len(SETS[s]["A"]) for s in SPECIES_ORDER)},'
 f'B={min(len(SETS[s]["B"]) for s in SPECIES_ORDER)}-'
 f'{max(len(SETS[s]["B"]) for s in SPECIES_ORDER)},'
 f'S0={len(SETS[SPECIES_ORDER[0]]["S0"])}.')
gA = comp[comp.Set == 'A'].Mean_GC3.mean(); gS = comp[comp.Set == 'S0'].Mean_GC3.mean()
print(f' (4) GC3:A={gA:.4f} -> B={comp[comp.Set=="B"].Mean_GC3.mean():.4f} -> '
 f'S0={gS:.4f}( GC3 {(gS-gA)/gA*100:.1f}%).')
print(f' (5) :A={len(shared["A"])},B={len(shared["B"])},'
 f'S0={len(shared["S0"])}; {core}.')
print(f' (6) ( {exc_df.Length_bp.median():.0f} bp / '
 f'{exc_df.Codons.median():.0f} vs {np.median(ret_len):.0f} bp),'
 f' GC3 ({exc_df.GC3.mean():.4f} vs {np.mean(ret_gc3):.4f}).')
print(f' -> :,'
 f'"".')
print(SEP); print(f'Output directory: {OUT_DIR}'); print(SEP)
```

Step 15. 2D Filter Sweep (R2 #8 supp.)

2x5 factorial: start-codon rule x length threshold (150-500bp).

Input: raw.fasta + filtered + ENC.xlsx

Output: Fig_R7 sweep_2D.pdf, results.xlsx

Method: 2x5 factorial; CodonW batch

Source Code (step15_filter_sweep_2D.py):

```
# -*- coding: utf-8 -*-
"""
Two-dimensional filter sweep (Comment 8 supplementary)
Two-dimensional filter sweep: start-codon rule × length threshold

Purpose: disentangle"start-codon filter"and"length filter",
 "use all valid annotated CDS for sensitivity analysis",
 >=300 bp threshold is arbitrary or justified.

Design:
 start rules 2 :ATG-only()|ATG/GTG/TTG()
 length thresholds 6 :0(),150,200,250,300(),400 bp
 -> 12 × 6 ,:,GC3,ENC,
 ,, and(8)

:
 - (ATG + >=300bp) ENC 5_ENC_plot/ENC.xlsx,;
 - CodonW 1.4.2 (, 80 CDS);
 - filter_cds.py(->->);
 - ().
"""

import os
import subprocess
import tempfile
from collections import defaultdict
import numpy as np
import pandas as pd
import matplotlib
matplotlib.use('Agg')
import matplotlib.pyplot as plt
from Bio import SeqIO
# ---------------------------------------------------------------------------
# Repository configuration (ADDED for public release - see CHANGES.md)
# Only the hard-coded local paths were parameterised; no algorithm, threshold,
# formula, sort order or output content was modified.
# CUB_DATA_DIR : directory holding the input data (default:../data)
# CUB_OUT_DIR : directory for outputs (default:../output)
# ---------------------------------------------------------------------------
import os as _os
_HERE = _os.path.dirname(_os.path.abspath(__file__))
DATA_DIR = _os.environ.get("CUB_DATA_DIR", _os.path.normpath(_os.path.join(_HERE,
_os.pardir, "data")))
OUT_DIR = _os.environ.get("CUB_OUT_DIR", _os.path.normpath(_os.path.join(_HERE,
_os.pardir, "output")))
_os.makedirs(OUT_DIR, exist_ok=True)
# ---------------------------------------------------------------------------

matplotlib.rcParams['pdf.fonttype'] = 42
```

```
matplotlib.rcParams['ps.fonttype'] = 42
matplotlib.rcParams['font.family'] = 'Times New Roman'
matplotlib.rcParams['mathtext.fontset'] = 'stix'

WS = os.path.normpath(os.path.join(os.path.dirname(os.path.abspath(__file__)), os.pardir))
RAW_DIR = os.path.join(WS, "data")
DATA = os.path.join(WS, "data")
ENC_XLSX = os.path.join(WS, "data", "ENC.xlsx")
OUT_DIR = os.environ.get("CUB_OUT_DIR", os.path.join(WS, "output"))
CODONW = os.environ.get("CUB_CODONW_EXE", os.path.join(WS, "external", "CodonW.exe"))

SPECIES_ORDER = ["C. lanatus", "C. melo", "C. sativus",
 "C. moschata", "B. hispida", "M. charantia"]
RAW_FASTA = {
 "C. lanatus": "watermelon_CDS.fasta", "C. melo": "melon_CDS.fasta",
 "C. sativus": "cucumber_CDS.fasta", "C. moschata": "pumpkin_CDS.fasta",
 "B. hispida": "winter melon_CDS.fasta", "M. charantia": "bitter melon_CDS.fasta",
}
FILTERED_FASTA = {
 "C. lanatus": "watermelon_CDS_filtered.fasta", "C. melo": "melon_CDS_filtered.fasta",
 "C. sativus": "cucumber_CDS_filtered.fasta", "C. moschata": "pumpkin_CDS_filtered.fasta",
 "B. hispida": "winter melon_CDS_filtered.fasta",
 "M. charantia": "bitter melon_CDS_filtered.fasta",
}
START_RULES = {'ATG-only (manuscript)': {'ATG'},
 'ATG/GTG/TTG (relaxed)': {'ATG', 'GTG', 'TTG'}}
LENGTHS = [0, 150, 200, 250, 300, 400]
SHARED8 = {'ACT', 'ATT', 'CAA', 'CGT', 'GAC', 'GCT', 'GGT', 'TTC'}

CODON_TABLE = {
 'TTT':'F','TTC':'F','TTA':'L','TTG':'L','TCT':'S','TCC':'S','TCA':'S','TCG':'S',
 'TAT':'Y','TAC':'Y','TAA':'*','TAG':'*','TGT':'C','TGC':'C','TGA':'*','TGG':'W',
 'CTT':'L','CTC':'L','CTA':'L','CTG':'L','CCT':'P','CCC':'P','CCA':'P','CCG':'P',
 'CAT':'H','CAC':'H','CAA':'Q','CAG':'Q','CGT':'R','CGC':'R','CGA':'R','CGG':'R',
 'ATT':'I','ATC':'I','ATA':'I','ATG':'M','ACT':'T','ACC':'T','ACA':'T','ACG':'T',
 'AAT':'N','AAC':'N','AAA':'K','AAG':'K','AGT':'S','AGC':'S','AGA':'R','AGG':'R',
 'GTT':'V','GTC':'V','GTA':'V','GTG':'V','GCT':'A','GCC':'A','GCA':'A','GCG':'A',
 'GAT':'D','GAC':'D','GAA':'E','GAG':'E','GGT':'G','GGC':'G','GGA':'G','GGG':'G',
}
SEP = '=' * 78


def calc_gc3(seq):
 s = str(seq).upper()
 cs = [s[i:i+3] for i in range(0, len(s)-2, 3) if len(s[i:i+3]) == 3]
 if len(cs) < 5:
 return None
 return sum(1 for c in cs if c[2] in 'GC') / len(cs)


def calc_rscu(seq):
 s = str(seq).upper()
 cs = [s[i:i+3] for i in range(0, len(s)-2, 3)]
 v = [c for c in cs if c in CODON_TABLE and CODON_TABLE[c] != '*']
 if len(v) < 10:
 return None
 ac = defaultdict(lambda: defaultdict(int)); at = defaultdict(int)
 for c in v:
 a = CODON_TABLE[c]; ac[a][c] += 1; at[a] += 1
 r = {}
```

```
 for a in at:
 n = len(ac[a])
 if n <= 1:
 continue
 for c, k in ac[a].items():
 r[c] = (k/at[a])*n
 return r


def apply_filter(records, starts, min_len):
 """: -> ->. {gene: seq}."""
 kept, seen = {}, set()
 for g, s in records:
 if s[:3] not in starts:
 continue
 if len(s) < min_len:
 continue
 if g in seen:
 continue
 seen.add(g)
 kept[g] = s
 return kept


def codonw_enc(seqs, tag):
 if not seqs:
 return {}
 work = os.path.join(tempfile.gettempdir(), 'cw_sweep', tag)
 os.makedirs(work, exist_ok=True)
 out_t = os.path.join(work, 'out.txt')
 with open(os.path.join(work, 'in.fasta'), 'w') as f:
 for g, s in seqs.items():
 f.write(f'>{g}\n{s}\n')
 for p in (out_t, os.path.join(work, 'blk.txt')):
 if os.path.exists(p):
 os.remove(p)
 subprocess.run([CODONW, 'in.fasta', 'out.txt', 'blk.txt', '-nomenu',
 '-silent', '-enc'], cwd=work, capture_output=True,
 text=True, check=True)
 lines = [l for l in open(out_t).read().splitlines() if l.strip()]
 hdr = [h.strip() for h in lines[0].split('\t')]
 iN = hdr.index('Nc')
 res = {}
 for l in lines[1:]:
 parts = l.rstrip('\t').split('\t')
 if len(parts) > iN:
 try:
 res[parts[0].strip()] = float(parts[iN])
 except ValueError:
 pass
 return res


def optimal(gene_enc, gene_rscu, pct=0.10, rscu_cut=1.0, d_cut=0.08):
 common = [g for g in gene_rscu if gene_enc.get(g) is not None]
 if len(common) < 10:
 return set(), 0, len(common)
 o = sorted(((g, gene_enc[g]) for g in common), key=lambda kv: (kv[1], kv[0]))
 ng = max(1, int(len(o)*pct))
 high = [g for g, _ in o[:ng]]; low = [g for g, _ in o[-ng:]]
```

```
 hr, lr = defaultdict(list), defaultdict(list)
 for g in high:
 for c, v in gene_rscu[g].items(): hr[c].append(v)
 for g in low:
 for c, v in gene_rscu[g].items(): lr[c].append(v)
 ha = {c: np.mean(v) for c, v in hr.items()}
 la = {c: np.mean(v) for c, v in lr.items()}
 return ({c for c in set(ha)|set(la)
 if ha.get(c, 0) > rscu_cut and (ha.get(c, 0)-la.get(c, 0)) >= d_cut},
 ng, len(common))


print(SEP); print('0. and'); print(SEP)
enc_df = pd.read_excel(ENC_XLSX)
ref_enc = {(r.Species, str(r.Gene)): float(r.ENC) for r in enc_df.itertuples()}

RECS, MS_FASTA, ENC = {}, {}, {}
for sp in SPECIES_ORDER:
 RECS[sp] = [(r.id, str(r.seq).upper())
 for r in SeqIO.parse(os.path.join(RAW_DIR, RAW_FASTA[sp]), 'fasta')]
 MS_FASTA[sp] = {r.id: str(r.seq).upper() for r in
 SeqIO.parse(os.path.join(DATA, FILTERED_FASTA[sp]), 'fasta')}
 A = apply_filter(RECS[sp], {'ATG'}, 300)
 assert set(A) == set(MS_FASTA[sp]), f'{sp}: A≠FASTA'
 assert all(A[g] == MS_FASTA[sp][g] for g in A), f'{sp}: A≠FASTA'
 ENC[sp] = {g: ref_enc[(sp, g)] for g in MS_FASTA[sp]}
 print(f' {sp:<15} CDS={len(RECS[sp]):>3} A={len(A):>3} '
 f'(+)')

print()
print(' ENC(CodonW 1.4.2,)...')
S0 = {}
for sp in SPECIES_ORDER:
 d = {}
 for g, s in RECS[sp]:
 if g not in d:
 d[g] = s
 for g in d:
 if g in MS_FASTA[sp]:
 d[g] = MS_FASTA[sp][g]
 S0[sp] = d
 new = {g: s for g, s in d.items() if g not in ENC[sp]}
 got = codonw_enc(new, sp.replace(' ', '_').replace('.', ''))
 ENC[sp].update(got)
 noenc = sorted(set(new) - set(got))
 print(f' {sp:<15} CDS()={len(d):>3} ={len(got):>3} '
 f'CodonW={noenc if noenc else "None"}')

print(); print(SEP); print('1. : × length thresholds'); print(SEP)
rows = []
for sname, starts in START_RULES.items():
 for L in LENGTHS:
 per_sp, shared_sets = {}, {}
 for sp in SPECIES_ORDER:
 gs = apply_filter(RECS[sp], starts, L)
 for g in gs:
 if g in MS_FASTA[sp]:
 gs[g] = MS_FASTA[sp][g]
 rscu = {}
```

```
 gc3s, encs = [], []
 for g, s in gs.items():
 r = calc_rscu(s)
 if r:
 rscu[g] = r
 v = calc_gc3(s)
 if v is not None:
 gc3s.append(v)
 e = ENC[sp].get(g)
 if e is not None:
 encs.append(e)
 opt, ng, nusable = optimal(ENC[sp], rscu)
 per_sp[sp] = opt
 shared_sets[sp] = opt
 rows.append({
 'Start_rule': sname, 'Min_length_bp': L, 'Species': sp,
 'N_genes': len(gs), 'N_genes_usable': nusable,
 'N_per_extreme_group': ng,
 'Mean_GC3': round(float(np.mean(gc3s)), 4) if gc3s else None,
 'Mean_ENC': round(float(np.mean(encs)), 2) if encs else None,
 'N_optimal': len(opt), 'Optimal_codons': ', '.join(sorted(opt)),
 'N_overlap_with_manuscript8': len(opt & SHARED8),
 'Jaccard_with_manuscript8': round(
 len(opt & SHARED8)/len(opt | SHARED8), 3) if (opt or SHARED8) else 1.0,
 })
 sh = set.intersection(*shared_sets.values())
 is_ms = (sname.startswith('ATG-only') and L == 300)
 for sp in SPECIES_ORDER:
 rows[-(6 - SPECIES_ORDER.index(sp))]['SHARED_all_six'] = ', '.join(sorted(sh))
 rows[-(6 - SPECIES_ORDER.index(sp))]['N_shared'] = len(sh)
 rows[-(6 - SPECIES_ORDER.index(sp))]['N_shared_in_MS8'] = len(sh & SHARED8)
 flag = ' ← ' if is_ms else ''
 print(f' {sname:<24} >={L:>3}bp ={len(sh):>2} '
 f'{"8" if sh <= SHARED8 else ""} '
 f'| ={[len(shared_sets[s]) for s in SPECIES_ORDER]}{flag}')
 print(f' {"":<24} : {", ".join(sorted(sh))}')

sweep = pd.DataFrame(rows)

print(); print(SEP); print('2. '); print(SEP)
pv_n = sweep.pivot_table(index='Min_length_bp', columns='Start_rule',
 values='N_genes', aggfunc='mean').round(1)
pv_sh = sweep.drop_duplicates(['Start_rule', 'Min_length_bp']).set_index(
 ['Start_rule', 'Min_length_bp'])['N_shared'].unstack(0)
pv_gc3 = sweep.pivot_table(index='Min_length_bp', columns='Start_rule',
 values='Mean_GC3', aggfunc='mean').round(4)
pv_grp = sweep.pivot_table(index='Min_length_bp', columns='Start_rule',
 values='N_per_extreme_group', aggfunc='mean').round(1)
print('\n ():'); print(pv_n.to_string())
print('\n :'); print(pv_sh.to_string())
print('\n GC3:'); print(pv_gc3.to_string())
print('\n (deltaRSCU):'); print(pv_grp.to_string())

print('\n 8 ( ∩ 8):')
keep = sweep.drop_duplicates(['Start_rule', 'Min_length_bp']).set_index(
 ['Start_rule', 'Min_length_bp'])['N_shared_in_MS8'].unstack(0)
print(keep.to_string())

# ===== Plot =====
```

```
print(); print(SEP); print('3. Plot'); print(SEP)
fig, axes = plt.subplots(2, 2, figsize=(14, 10))
SC = {'ATG-only (manuscript)': '#08519C', 'ATG/GTG/TTG (relaxed)': '#B2182B'}
mk = {'ATG-only (manuscript)': 'o', 'ATG/GTG/TTG (relaxed)': 's'}

ax = axes[0, 0]
for sn in START_RULES:
 sub = sweep[sweep.Start_rule == sn].groupby('Min_length_bp').N_genes.mean()
 ax.plot(sub.index, sub.values, mk[sn]+'-', color=SC[sn], lw=2.1, ms=7, label=sn)
ax.axvline(300, color='grey', ls='--', lw=1.2)
ax.text(306, ax.get_ylim()[1]*0.95, 'manuscript\n300 bp', fontsize=8.6, color='grey')
ax.set_xlabel('Minimum CDS length (bp)', fontsize=11)
ax.set_ylabel('Mean number of genes per species', fontsize=11)
ax.set_title('(a) Observations retained vs length threshold', fontsize=12,
fontweight='bold')
ax.legend(fontsize=9.5); ax.grid(alpha=0.28, lw=0.7); ax.set_axisbelow(True)

ax = axes[0, 1]
for sn in START_RULES:
 sub = sweep[sweep.Start_rule == sn].drop_duplicates('Min_length_bp').set_index(
 'Min_length_bp')['N_shared']
 ax.plot(sub.index, sub.values, mk[sn]+'-', color=SC[sn], lw=2.1, ms=7, label=sn)
 for xi, yi in zip(sub.index, sub.values):
 ax.annotate(str(int(yi)), (xi, yi), textcoords='offset points',
 xytext=(0, 8), ha='center', fontsize=9, color=SC[sn])
ax.axhline(8, color='#08519C', ls=':', lw=1.3)
ax.axvline(300, color='grey', ls='--', lw=1.2)
ax.set_xlabel('Minimum CDS length (bp)', fontsize=11)
ax.set_ylabel('Codons shared by all six species', fontsize=11)
ax.set_title('(b) Shared optimal codons vs length threshold', fontsize=12,
fontweight='bold')
ax.set_ylim(0, 11.5); ax.legend(fontsize=9.5, loc='lower left')
ax.grid(alpha=0.28, lw=0.7); ax.set_axisbelow(True)

ax = axes[1, 0]
for sn in START_RULES:
 sub = sweep[sweep.Start_rule == sn].groupby('Min_length_bp').Mean_GC3.mean()
 ax.plot(sub.index, sub.values, mk[sn]+'-', color=SC[sn], lw=2.1, ms=7, label=sn)
ax.axvline(300, color='grey', ls='--', lw=1.2)
ax.set_xlabel('Minimum CDS length (bp)', fontsize=11)
ax.set_ylabel('Mean GC3', fontsize=11)
ax.set_title('(c) Mean GC3 rises as short, GC3-rich CDSs are admitted',
 fontsize=12, fontweight='bold')
ax.legend(fontsize=9.5); ax.grid(alpha=0.28, lw=0.7); ax.set_axisbelow(True)

ax = axes[1, 1]
for sn in START_RULES:
 sub = sweep[sweep.Start_rule == sn].groupby('Min_length_bp').N_per_extreme_group.mean()
 ax.plot(sub.index, sub.values, mk[sn]+'-', color=SC[sn], lw=2.1, ms=7, label=sn)
ax.axvline(300, color='grey', ls='--', lw=1.2)
ax.set_xlabel('Minimum CDS length (bp)', fontsize=11)
ax.set_ylabel('Mean genes per extreme (10%) group', fontsize=11)
ax.set_title('(d) Group size available for deltaRSCU estimation',
 fontsize=12, fontweight='bold')
ax.legend(fontsize=9.5); ax.grid(alpha=0.28, lw=0.7); ax.set_axisbelow(True)

fig.suptitle('Two-dimensional filter sweep: start-codon rule × length threshold '
 '(Reviewer 2, Comment 8)', fontsize=14, fontweight='bold', y=0.99)
fig.tight_layout(rect=[0, 0, 1, 0.962])
```

```
f1 = os.path.join(OUT_DIR, 'Fig_R7_filter_sweep_2D.pdf')
fig.savefig(f1, bbox_inches='tight')
fig.savefig(os.path.join(OUT_DIR, 'Fig_R7_filter_sweep_2D.png'), dpi=300,
 bbox_inches='tight')
plt.close(fig)
print(f' : {os.path.basename(f1)}')

out_xlsx = os.path.join(OUT_DIR, 'filter_sweep_2D_results.xlsx')
with pd.ExcelWriter(out_xlsx, engine='openpyxl') as wr:
 sweep.to_excel(wr, sheet_name='1_full_sweep', index=False)
 pv_n.to_excel(wr, sheet_name='2_mean_N_genes')
 pv_sh.to_excel(wr, sheet_name='3_N_shared_codons')
 pv_gc3.to_excel(wr, sheet_name='4_mean_GC3')
 pv_grp.to_excel(wr, sheet_name='5_group_size')
 keep.to_excel(wr, sheet_name='6_MS8_retained')
print(f' Results table saved: {os.path.basename(out_xlsx)}')

print(); print(SEP); print('4. '); print(SEP)
ms = sweep[(sweep.Start_rule.str.startswith('ATG-only')) & (sweep.Min_length_bp == 300)]
print(f' (1) (ATG-only + >=300bp) = '
 f'{sorted(set.intersection(*[set(r.Optimal_codons.split(", ")) for r in
ms.itertuples()]))}'
 if False else
 f' (1) (ATG-only + >=300bp) = {ms.SHARED_all_six.iloc[0]}')
print(f' (2) (ATG/GTG/TTG + >=300bp): = '
 f'{sweep[(sweep.Start_rule.str.startswith("ATG/GTG")) &
(sweep.Min_length_bp==300)].SHARED_all_six.iloc[0]}')
print(f' (3) ( CDS) (b).')
print(SEP); print(f'Output directory: {OUT_DIR}'); print(SEP)
```

5. How to Run

pip install -r requirements.txt

python run_all.py # all steps + verify

python run_all.py --verify-only # verify only

python run_all.py --steps 1, 2, 8 # selected steps

Author Contribution to this Supplementary Material

All custom Python scripts presented in this document, including CDS extraction and filtering, RSCU/ENC calculation, optimal codon identification, plotting routines, and the complete parameterized workflow, were independently designed, written, tested, and verified by the first author, Yongjie Xia. Zhuoran Huang provided overall guidance and manuscript review. All scripts are reproducible from the provided source data.
