# Supplementary material for: Codon Usage Bias Analysis of Chloroplast Genomes in Six Cucurbitaceae Species
Source: Genes (Basel). 2026 Sep 11;17(9):1095. doi: 10.3390/genes17091095 (PMC13606370; doi:10.3390/genes17091095)
Supplement: Supplementary file 1 [file genes-17-01095-s001.zip › Supplementary_Materials.pdf]

Codon Usage Bias Analysis of Chloroplast Genomes in Six Cucurbitaceae Species

Supplementary Figures and Sensitivity Analyses for Codon Usage Bias of Chloroplast Genomes in Six Cucurbitaceae Species

Manuscript: genes-4534662 | Journal: Genes (MDPI)

Yongjie Xia [1] and **Zhuoran Huang** [1], [2], *

[1] College of Life Sciences, Huaibei Normal University, Huaibei 235000, China; 18805601148@163.com (Y.X.)
[2] Anhui Province Key Laboratory of Pollutant Sensitive Materials and Environmental Remediation, Huaibei Normal University, Huaibei 235000, China
* Correspondence: huangzr@chnu.edu.cn

This document contains two parts:

Part I. High-resolution versions of main-text figures (**Figures S1–S2**)

Part II. Sensitivity analyses and robustness checks (**Figures S3–S9**)

All supplementary tables (**Tables S1–S10**) are provided in the accompanying Excel file (**Supplementary_Tables.xlsx**).

Part I. High-Resolution Versions of Main-Text Figures

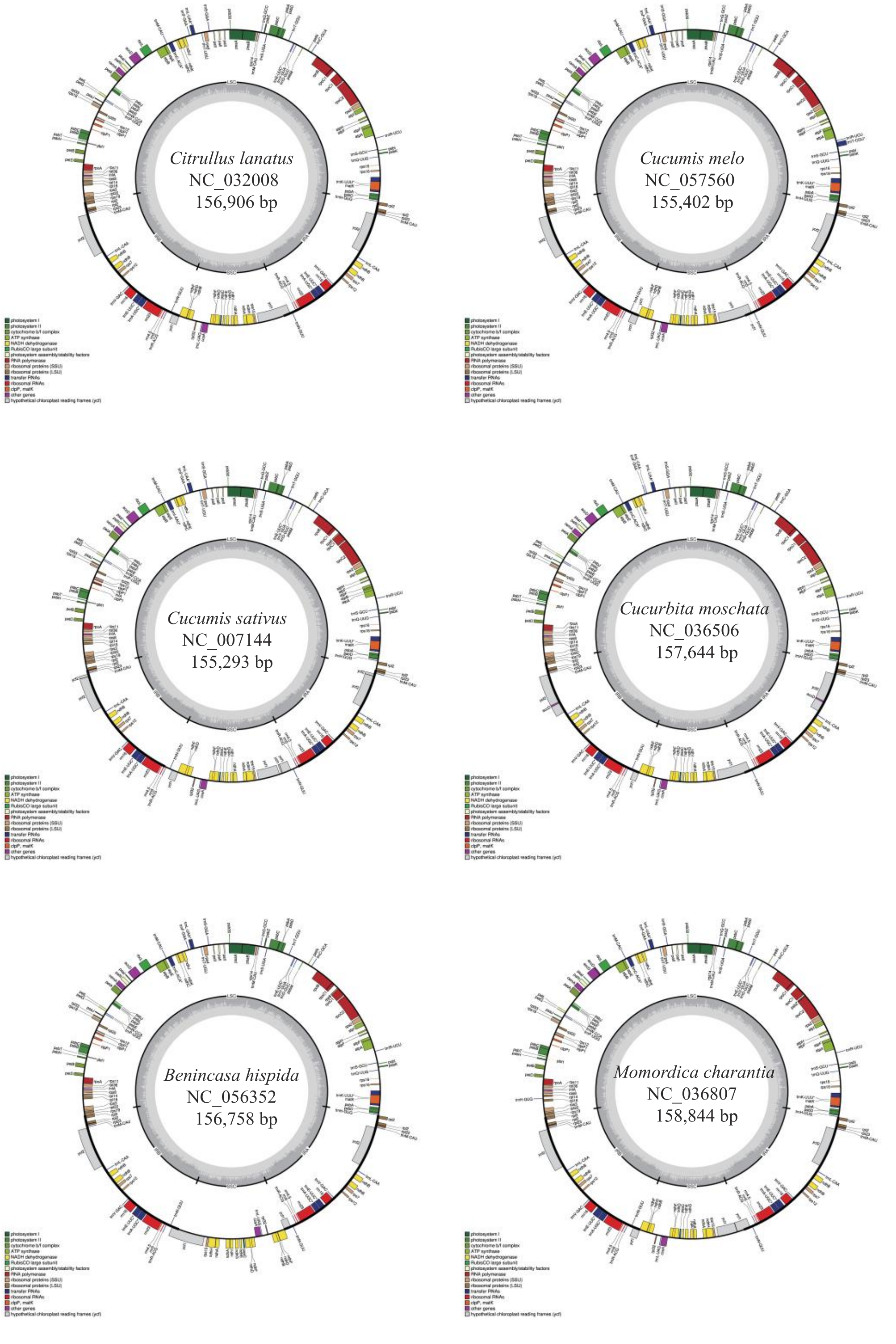

Figure S1. Circular genome maps of the six chloroplast genomes. Genes are annotated on the outer circle; genes outside the circle are transcribed clockwise, and those inside are transcribed counterclockwise. The inner circles indicate GC content and GC skew.

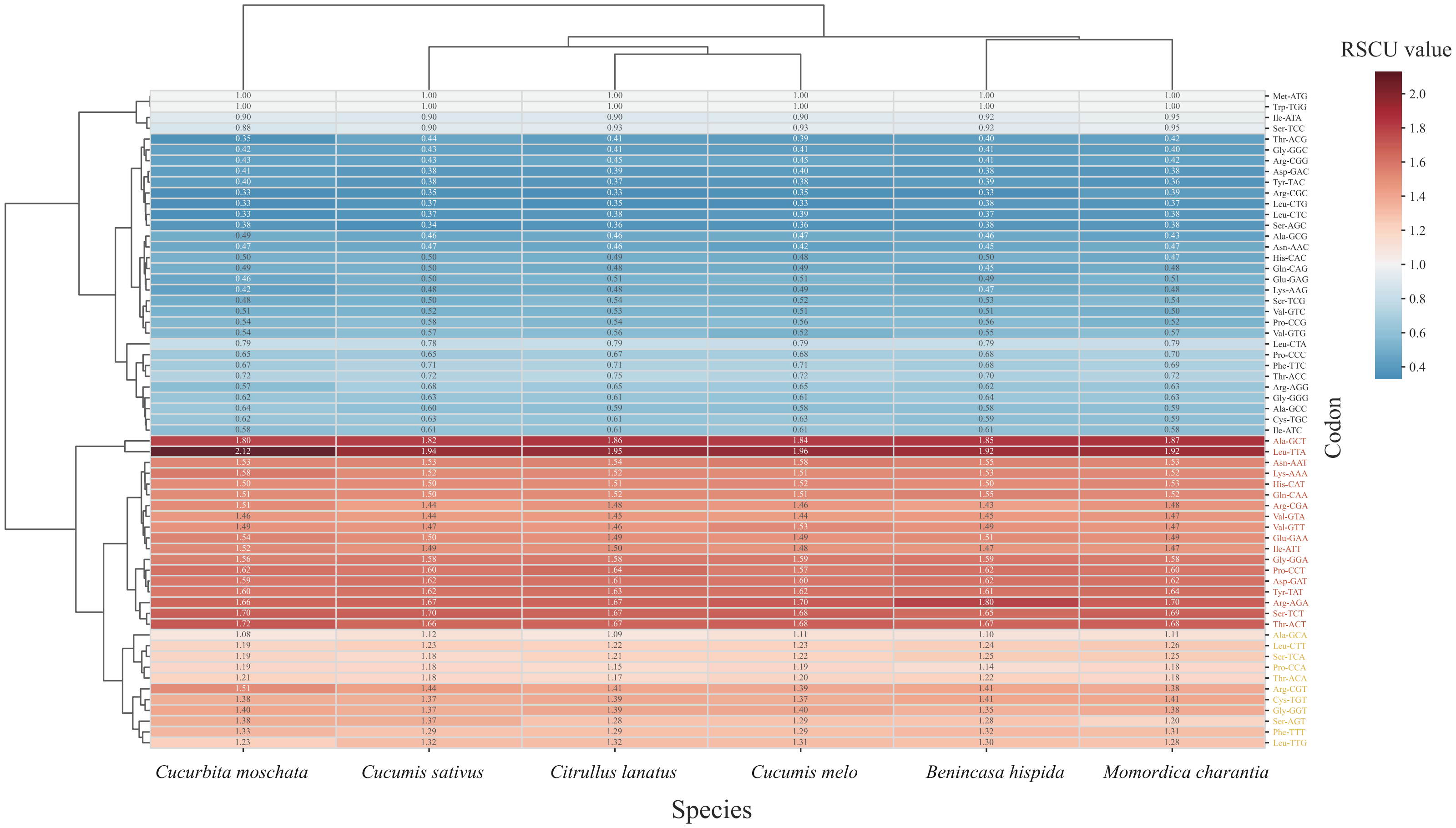

Figure S2. RSCU heatmap of codon usage across six Cucurbitaceae species (high-resolution version). Rows represent codons (labeled as amino acid–codon) and columns represent the six species; hierarchical clustering dendrograms are shown for both axes. RSCU values ≤ 1 are shown in dark tones; values > 1 are shown in an orange-to-red gradient, with red indicating the strongest usage bias.

Part II. Sensitivity Analyses and Robustness Checks

This part contains three sets of sensitivity analyses conducted to assess the robustness of the main findings regarding:

(1) correlation structure among codon usage parameters,

(2) optimal codon identification, and

(3) CDS filtering criteria. All analyses were performed using Python with the same filtered CDS datasets used in the main text. Full numerical results are provided in the supplementary Excel workbook.

1. Per-Species and Gene-Level Correlation Analysis

In response to the concern that pooling homologous genes from six species violates observation independence, correlations among ENC, GC1, GC2, and GC3 were re-analysed at three levels: (i) per-species (n = 39–41 genes); (ii) gene-level (each of 41 genes represented by its cross-species mean); and (iii) pooled (n = 242, presented descriptively). Benjamini–Hochberg FDR correction was applied within each level and globally.

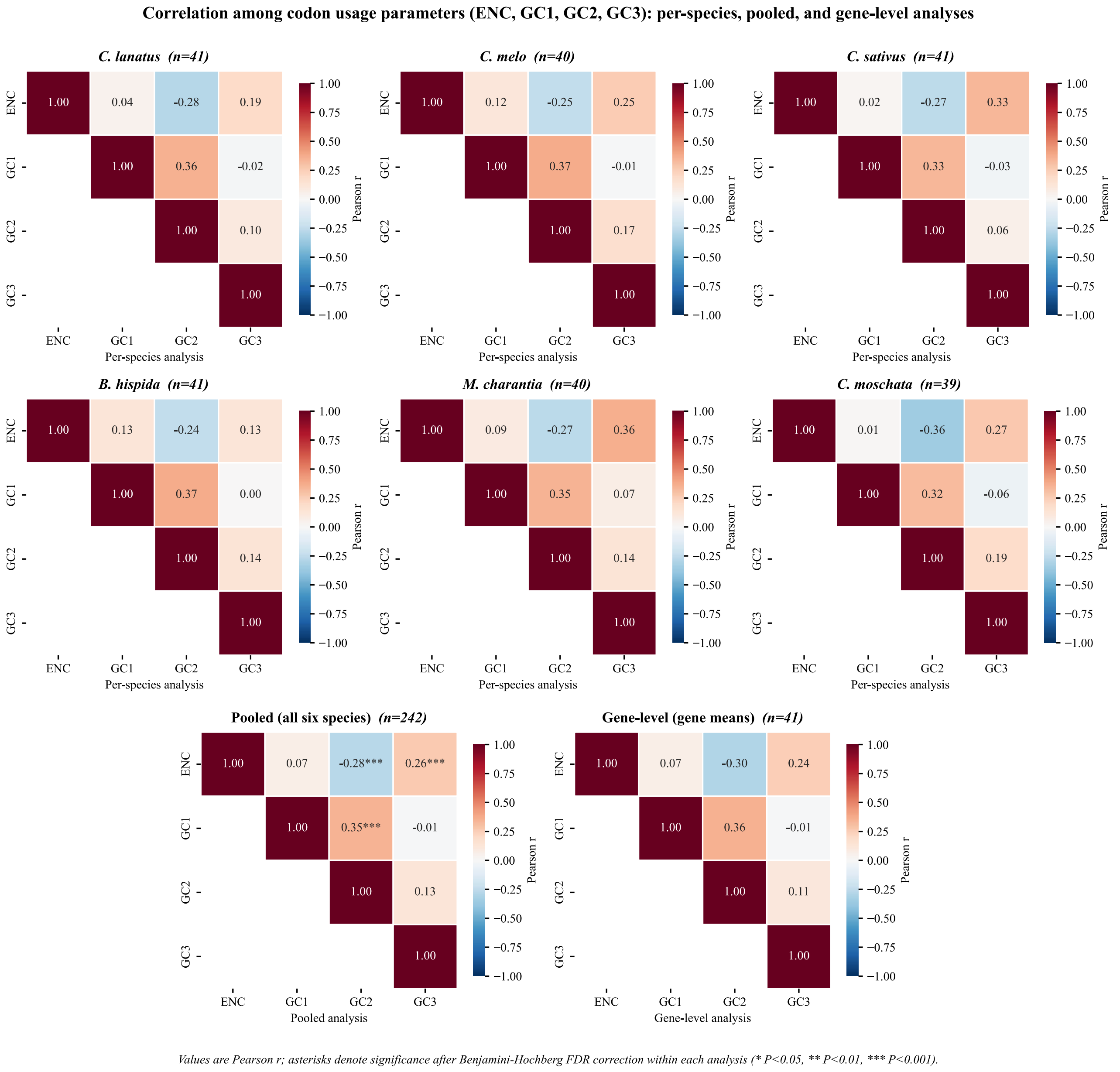

Figure S3. Per-species and gene-level correlation heatmaps.

Figure S3 shows Pearson (lower triangle) and Spearman (upper triangle) correlation coefficients for each species, the pooled dataset, and the gene-level dataset. Effect sizes are highly consistent across levels.

The effect-size consistency across the pooled, per-species, and gene-level analyses is summarized below (full data in **Table S7** of the supplementary Excel workbook):

Pair	Pooled r	Per-sp. mean r	SD	Gene-level r	Same dir.
ENC–GC1	0.069	0.069	0.050	0.069	6/6
ENC–GC2	−0.277	−0.279	0.042	−0.305	6/6
ENC–GC3	0.258	0.256	0.085	0.244	6/6
GC1–GC2	0.349	0.349	0.022	0.355	6/6
GC1–GC3	−0.010	−0.009	0.045	−0.013	4/6
GC2–GC3	0.131	0.131	0.045	0.114	6/6

For all six parameter pairs, the pooled r differs from the mean per-species r by less than 0.10, and five of six pairs show the same direction in all six species. Per-species tests frequently lost significance after FDR correction (0 of 36 tests significant; see Table S7, Part C), consistent with limited statistical power at n = 39–41 (minimum detectable |r| ≈ 0.44 at 80% power; see Table S7, Part B).

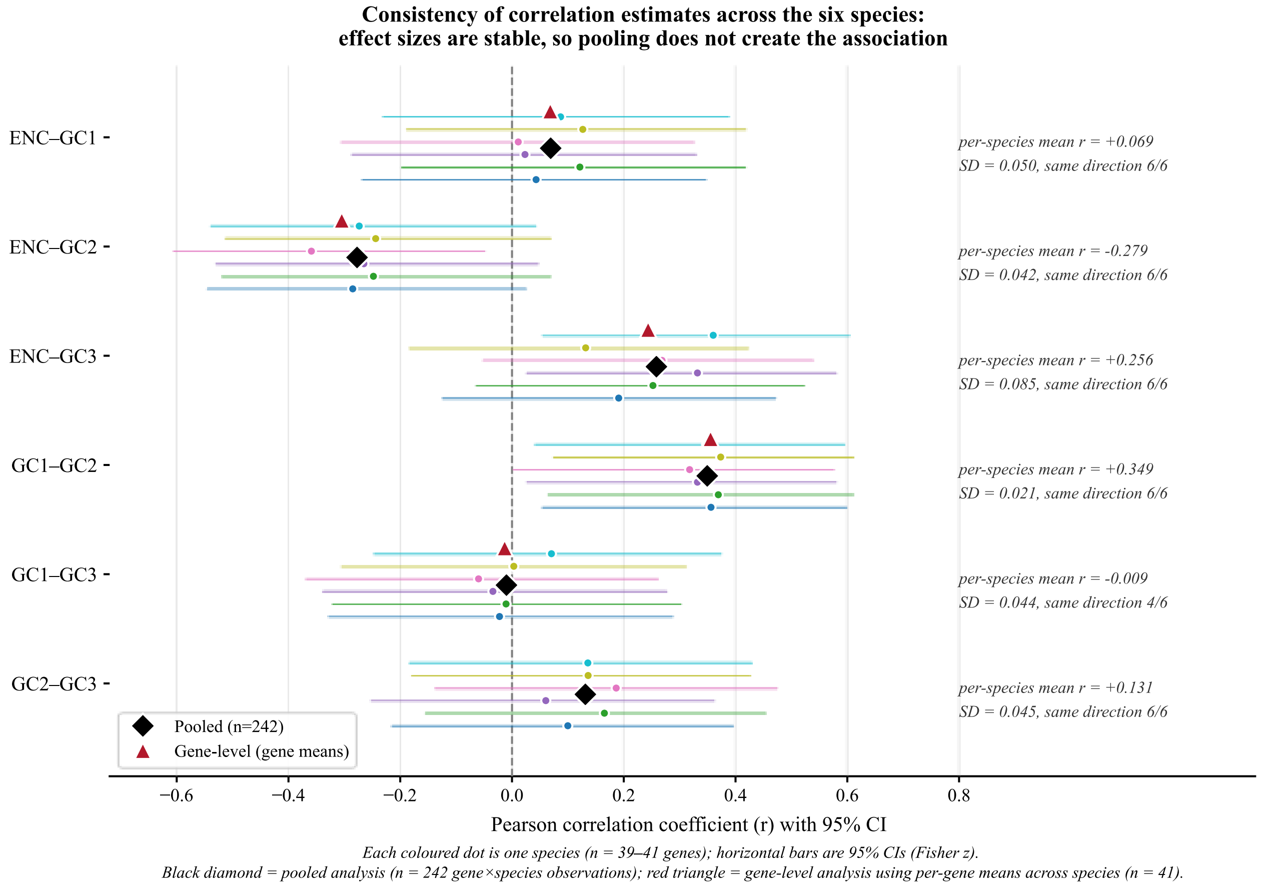

Figure S4. Cross-species consistency of effect sizes (forest plot).

Figure S4 compares the pooled correlation coefficient (diamond) with the 95% confidence intervals of each per-species coefficient for the six parameter pairs. The pooled point estimate falls within or near the per-species confidence intervals for every pair, confirming effect-size consistency. The ENC–GC2 pair (pooled r = −0.277) and GC1–GC2 pair (pooled r = +0.349) show the strongest and most consistent associations across species.

2. Optimal Codon Stability Analysis

With ~40 genes per species, the high- and low-bias groups contain only 3–4 genes each, making ΔRSCU sensitive to genes near the cutoff. Four sensitivity tests were performed: (i) group-size sweep (5%–25%); (ii) ΔRSCU threshold sweep (0.04–0.20); (iii) bootstrap resampling (B = 1000 per species); and (iv) leave-one-out analysis.

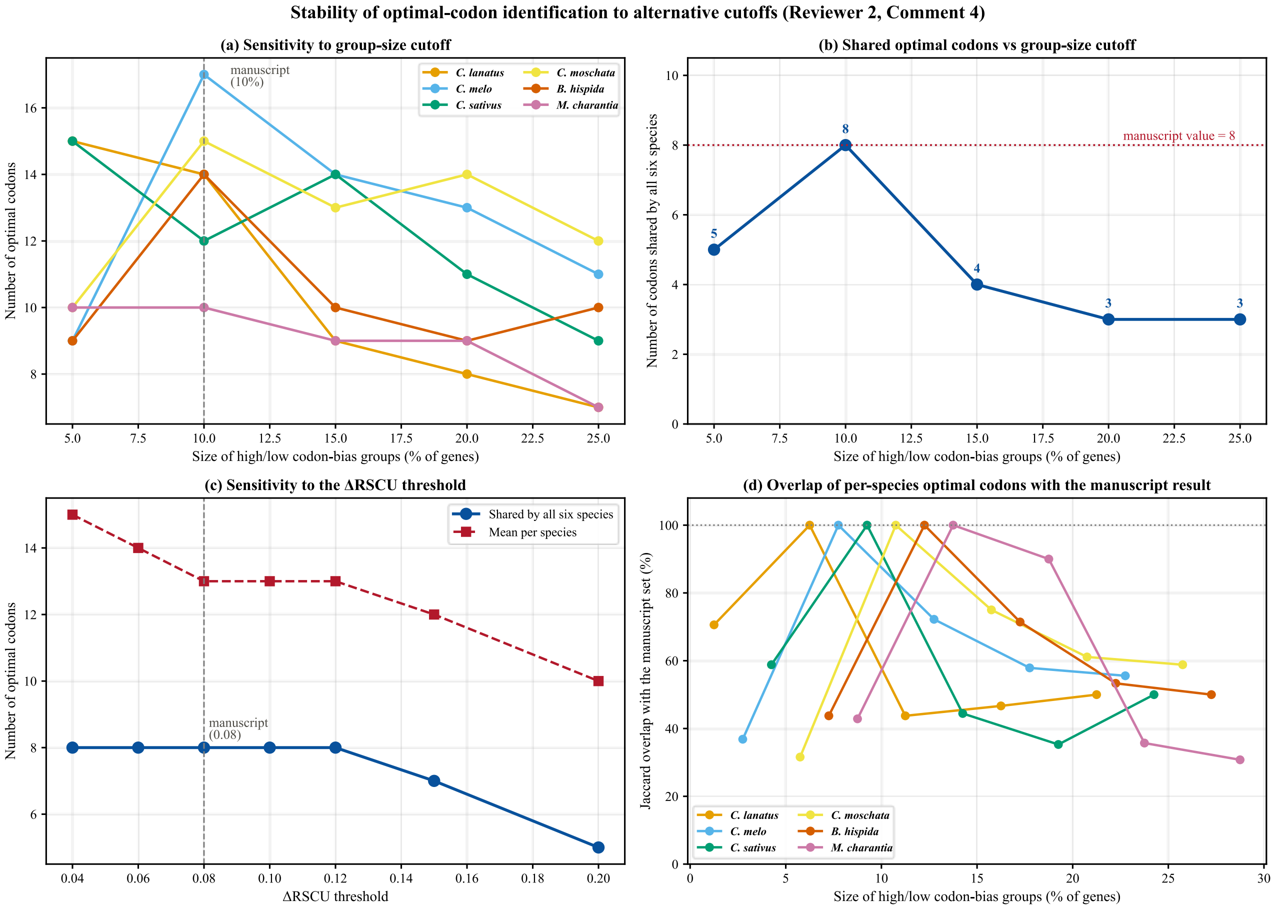

Figure S5. Optimal codon cutoff sensitivity (group size and ΔRSCU).

Figure S5 shows the number of codons shared by all six species as a function of group size (left) and ΔRSCU threshold (right). The eight shared codons identified in the main text are stable across ΔRSCU thresholds of 0.04–0.12. Group size has a larger effect: the shared set varies between 3 and 8 codons across 5%–25%, indicating genuine sensitivity to this parameter. The manuscript's 10% cutoff lies within the range that recovers the full eight-codon set.

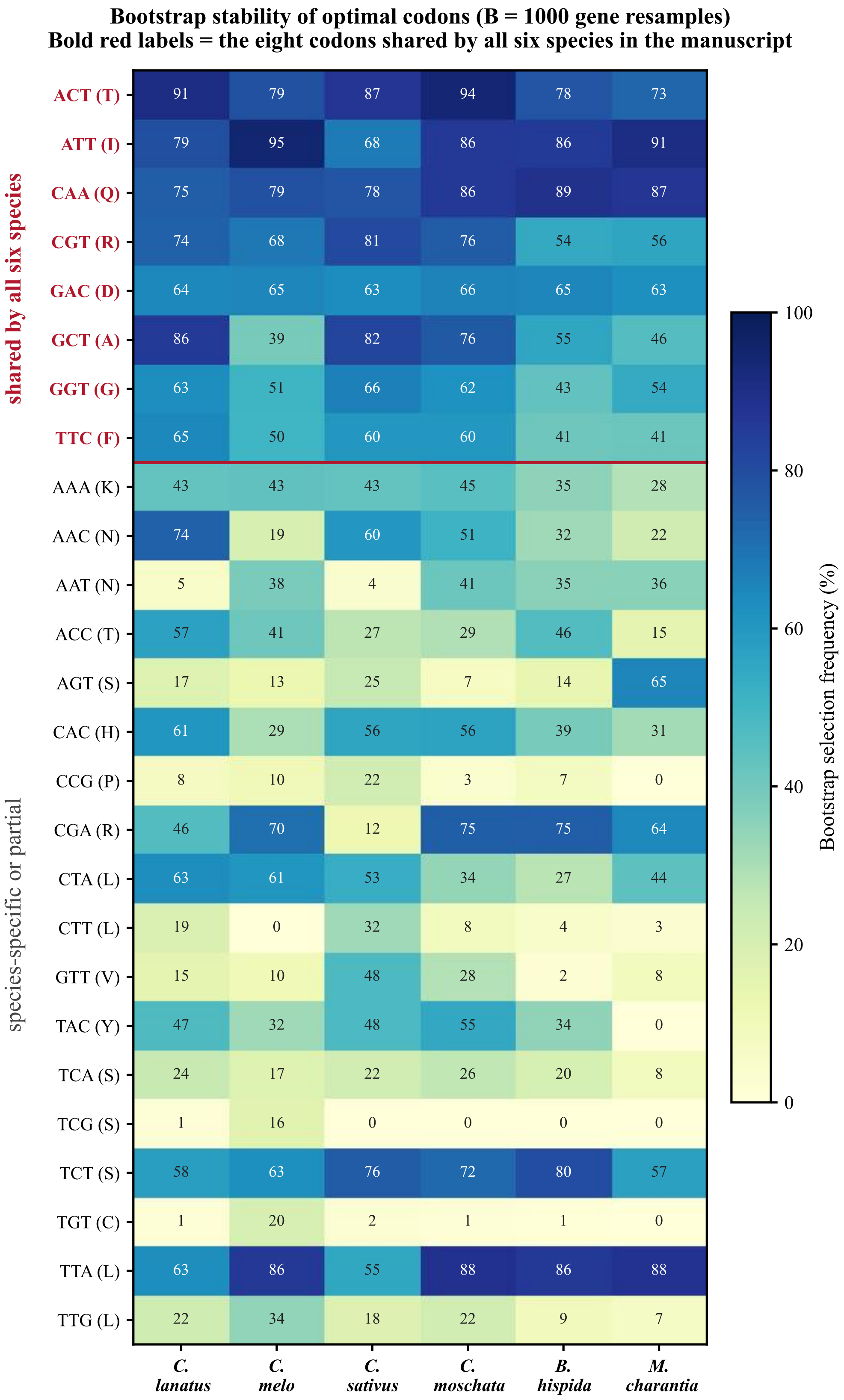

Figure S6. Bootstrap stability of shared optimal codons (B=1000).

Figure S6 shows bootstrap recovery frequencies (B = 1000) for the eight shared codons in each species.

Bootstrap recovery frequencies of the eight shared codons are summarized below (full data in **Table S8**):

Codon	AA	*C.lan.*	*C.melo*	*C.sat.*	*C.mosc.*	*B.his.*	*M.char.*	Core
ACT	Thr	91.0	78.7	86.9	93.9	77.7	72.7	Yes
ATT	Ile	79.2	94.6	67.7	85.9	85.5	90.7	Yes
CAA	Gln	75.0	78.9	77.7	86.1	88.8	86.9	Yes
GAC	Asp	64.5	65.0	63.4	65.8	65.0	62.8	Yes
CGT	Arg	74.1	68.4	80.8	75.7	54.0	55.7	No
GCT	Ala	85.5	38.9	82.3	76.3	55.3	46.0	No
GGT	Gly	63.0	50.7	66.5	61.8	43.3	53.8	No
TTC	Phe	64.7	50.3	60.3	60.4	40.7	41.4	No

Four codons, ACT, ATT, CAA, and GAC, are recovered in ≥62% of replicates in every species and constitute a robust core. The remaining four codons (CGT, GCT, GGT, TTC) show more variable recovery (39%–86%), representing the sensitive periphery. Leave-one-out analysis confirms that removing a single group member primarily exchanges marginal codons; the high- and low-codon-bias group compositions are biologically coherent and conserved across species (e.g., psbA and rps8 in the high-bias group, rpl2 in the low-bias group in all six genomes; see Table S5, Part A).

3. CDS Filter Sensitivity Analysis

Three nested filtering sets were compared: (A) manuscript filters (ATG initiation, ≥300 bp; 39–41 genes per species); (B) relaxed initiation (ATG/GTG/TTG, ≥300 bp; 41–42 genes); and (S0) all annotated CDSs (no filter; ~80 genes). A two-dimensional sweep of start-codon rule × length threshold (0–400 bp) was also performed.

Summary of the three nested filtering sets is shown below (full data in **Table S9**):

Set	Definition	Genes/sp.	Mean GC3	Mean ENC	Shared codons
A	ATG, ≥300 bp	39–41	0.281	47.30	8
B	ATG/GTG/TTG, ≥300 bp	41–42	0.284	47.27	8
S0	No filter	80	0.310	47.62	4

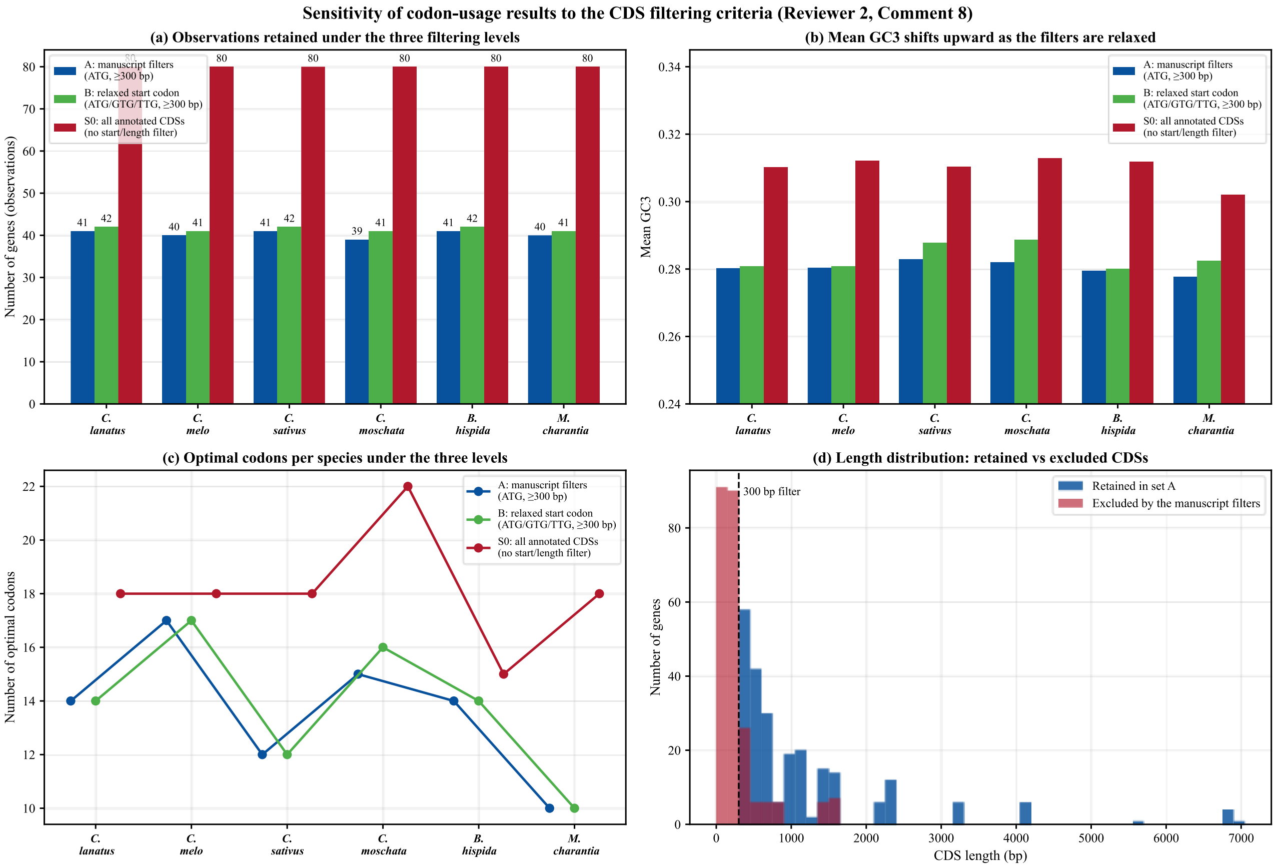

Figure S7. Filter sensitivity: three nested sets (A, B, S0).

Figure S7 compares ENC distributions, GC3 content, and optimal codon counts across the three filtering sets. Relaxing the start-codon criterion (A vs. B) adds only 1–2 genes per species and changes neither the per-species optimal codons nor the eight shared codons. Removing all filters (S0) admits very short CDSs (e.g., psb30, psbT, rpl36; 75–114 bp) for which per-gene RSCU cannot be estimated reliably and CodonW returns no ENC value.

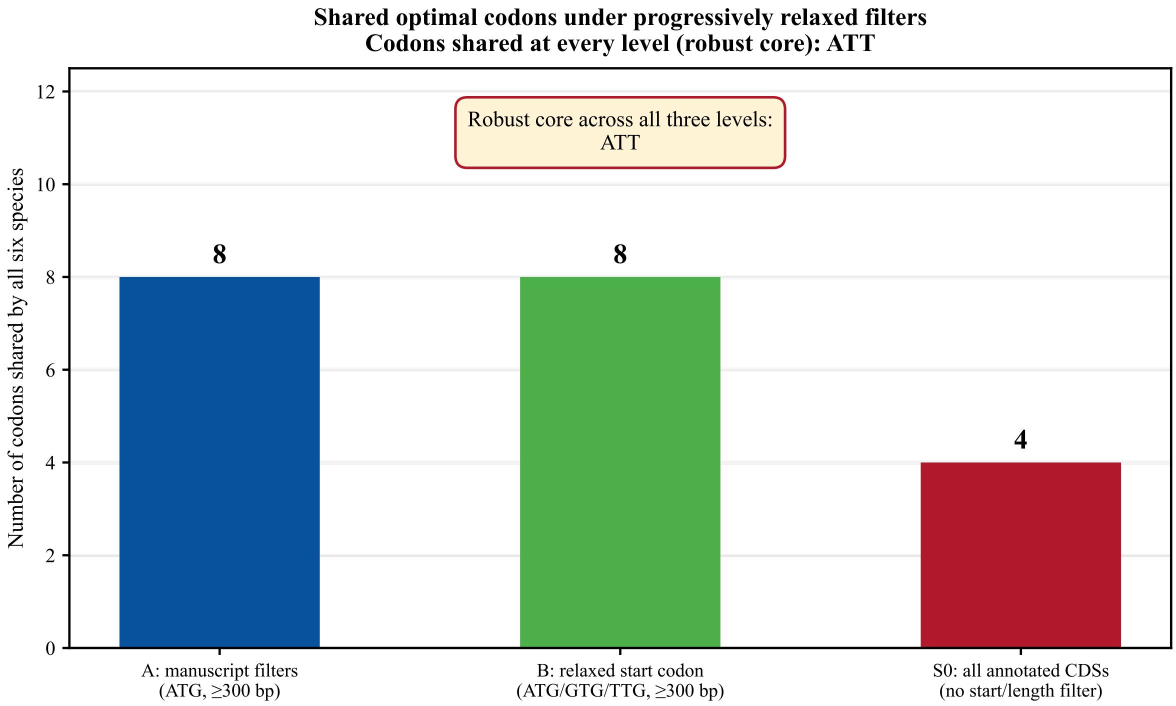

Figure S8. Shared codons across six species under different filters.

Figure S8 shows the number of codons shared by all six species under each filtering set. Sets A and B both yield 8 shared codons (ACT, ATT, CAA, CGT, GAC, GCT, GGT, TTC), identical to the main text. Set S0 (no filter) collapses to only 4 shared codons, confirming that the length filter is essential for stable results. The 300-bp threshold lies within a broad plateau (200–300 bp) over which all eight shared codons are retained under both start-codon rules (at 150 bp, the relaxed rule retains seven of the eight).

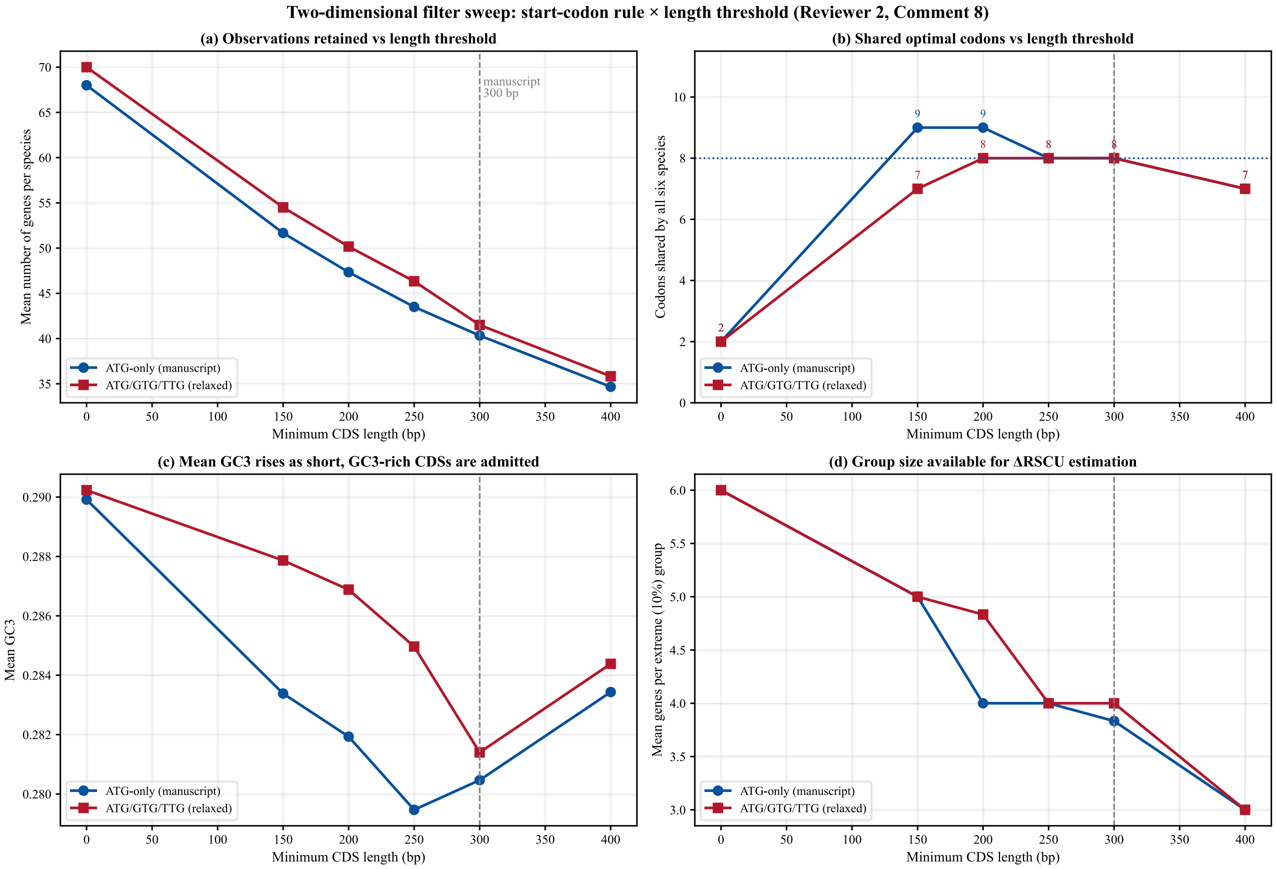

Figure S9. Two-dimensional filter sweep (start-codon rule × length threshold).

Figure S9 shows the two-dimensional sweep of start-codon rule (ATG-only vs. ATG/GTG/TTG) × minimum length (0–400 bp). The number of shared codons is stable at 8–9 across the 200–300 bp plateau for both start-codon rules (at 150 bp, the relaxed rule yields 7 shared codons while ATG-only yields 9). At 0 bp (no length filter), the shared set collapses to 2 codons regardless of start-codon rule. At 400 bp, 7 of 8 shared codons are retained. These results justify the manuscript's 300-bp criterion as a principled choice within a stable range, rather than an arbitrary threshold.

Author Contribution to this Supplementary Material

All sensitivity analyses presented in this document, including per-species and gene-level correlation reanalysis, optimal-codon cutoff and bootstrap stability tests, and CDS filter sensitivity sweeps, were independently designed, executed, and interpreted by the first author, Yongjie Xia. Zhuoran Huang provided overall guidance and manuscript review. All numerical results are reproducible from the provided scripts and source data.
